# Robust Virus-Specific Adaptive Immunity in COVID-19 Patients with SARS-CoV-2 Δ382 Variant Infection

**DOI:** 10.1007/s10875-021-01142-z

**Published:** 2021-10-30

**Authors:** Siew-Wai Fong, Nicholas Kim-Wah Yeo, Yi-Hao Chan, Yun Shan Goh, Siti Naqiah Amrun, Nicholas Ang, Menaka Priyadharsani Rajapakse, Josephine Lum, Shihui Foo, Cheryl Yi-Pin Lee, Guillaume Carissimo, Rhonda Sin-Ling Chee, Anthony Torres-Ruesta, Matthew Zirui Tay, Zi Wei Chang, Chek Meng Poh, Barnaby Edward Young, Paul A. Tambyah, Shirin Kalimuddin, Yee-Sin Leo, David C. Lye, Bernett Lee, Subhra Biswas, Shanshan Wu Howland, Laurent Renia, Lisa F. P. Ng

**Affiliations:** 1 A*STAR Infectious Diseases Labs (A*STAR ID Labs), Agency for Science, Technology and Research (A*STAR), Singapore City, Singapore; 2 Singapore Immunology Network, Agency for Science, Technology and Research (A*STAR), Singapore City, Singapore; 3 Department of Biochemistry, Yong Loo Lin School of Medicine, National University of Singapore, Singapore City, Singapore; 4 National Centre for Infectious Diseases, Singapore City, Singapore; 5 Department of Infectious Diseases, Tan Tock Seng Hospital, Singapore City, Singapore; 6 Lee Kong Chian School of Medicine, Nanyang Technological University, Singapore City, Singapore; 7 Department of Medicine, National University Hospital, Singapore City, Singapore; 8 Infectious Diseases Translational Research Programme, Department of Medicine, Yong Loo Lin School of Medicine, National University of Singapore, Singapore City, Singapore; 9 Department of Infectious Diseases, Singapore General Hospital, Singapore City, Singapore; 10 Programme in Emerging Infectious Diseases, Duke-NUS Medical School, Singapore City, Singapore; 11 Yong Loo Lin School of Medicine, National University of Singapore and National University Health System, Singapore City, Singapore; 12 NIHR Health Protection Research Unit in Emerging and Zoonotic Infections, University of Liverpool, Liverpool, UK; 13 Institute of Infection, Veterinary and Ecological Sciences, University of Liverpool, Liverpool, UK

**Keywords:** COVID-19, SARS-CoV-2, Transcriptome, ORF8, Adaptive immune response, CD4^+^ T cell response, CD8^+^ T cell response, Antibody response

## Abstract

The severe acute respiratory syndrome coronavirus 2 (SARS-CoV-2) variants of concern (VOCs) that have become dominant as the pandemic progresses bear the ORF8 mutation together with multiple spike mutations. A 382-nucleotide deletion (Δ382) in the ORF7b and ORF8 regions has been associated with milder disease phenotype and less systemic inflammation in COVID-19 patients. However, its impact on host immunity against SARS-CoV-2 remains undefined. Here, RNA-sequencing was performed to elucidate whole blood transcriptomic profiles and identify contrasting immune signatures between patients infected with either wildtype or Δ382 SARS-CoV-2 variant. Interestingly, the immune landscape of Δ382 SARS-CoV-2 infected patients featured an increased adaptive immune response, evidenced by enrichment of genes related to T cell functionality, a more robust SARS-CoV-2-specific T cell immunity, as well as a more rapid antibody response. At the molecular level, eukaryotic initiation factor 2 signaling was found to be upregulated in patients bearing Δ382, and its associated genes were correlated with systemic levels of T cell-associated and pro-inflammatory cytokines. This study provides more in-depth insight into the host–pathogen interactions of ORF8 with great promise as a therapeutic target to combat SARS-CoV-2 infection.

**Supplementary Information:**

The online version contains supplementary material available at 10.1007/s10875-021-01142-z.

## Introduction

Genetic mutation events might enable viruses to cross the species barrier to infect a new host and subsequently allow virus adaptation to the host [1–3]. Severe acute respiratory syndrome coronavirus 2 (SARS-CoV-2) has mutated throughout the pandemic, and multiple variants of concern (VOCs) continue to spread globally. The B.1.1.7 (alpha) VOC has become one of the SARS-CoV-2 strains in the United Kingdom since its emergence in September 2020 due to its increased transmissibility [4]. The P.1 (gamma) and B.1.351 (beta) variants are listed as VOCs as they are associated with enhanced transmissibility and decreased effectiveness of available therapeutics against coronavirus disease 2019 (COVID-19) [5]. The B.1.617.2 (delta) variant discovered initially in India has become the most dominant strain that largely drives the second or third wave of infections in multiple countries [6, 7]. It is thus important to assess the possible effects of various mutations present in these VOCs on the host immune landscape. Intriguingly, VOCs Alpha, Gamma and Delta variants have a mutation that potentially truncates the ORF8 protein or renders it inactive.

Associated with host adaptation and viral replication [8–10], the open reading frame (ORF) 8 region was identified as a mutation hotspot in SARS-CoV during the 2002–2003 outbreak [9]. Deletion events in the SARS-CoV-2 ORF8 region have been reported in several countries, including Singapore and Taiwan (382-nucleotide (nt) deletion, termed Δ382 SARS-CoV-2), Bangladesh (345-nt deletion), Australia (138-nt deletion), and Spain (52-nt deletion) [11, 12]. Several mutations in SARS-CoV-2 ORF8 protein have also been observed in multiple virus strains [13–15] and these variants account for around 5% of infections worldwide [16]. Although truncated ORF8 variants contribute to milder infections [17], they were the major variants in Asia and North America during the early pandemic [18]. Understanding the natural biology of host immune response following infection with a SARS-CoV-2 variant that causes a milder disease phenotype will provide important insights into preventive and therapeutic strategies for patient management and improve COVID-19 prognosis. Moreover, the findings could provide clues on the high transmissibility of VOCs that are currently in circulation.

While in vitro studies have suggested that SARS-CoV-2 ORF8 downregulates major histocompatibility complex (MHC)-I molecules and inhibits type I interferon signaling pathway [19, 20], the functional impacts of ORF8 deletion on the cellular host immune response against SARS-CoV-2 is unknown [17]. To decipher the underlying molecular mechanisms of this natural genetic deletion, a comprehensive characterization of the whole blood transcriptomic profiles between coronavirus disease 2019 (COVID-19) patients infected with wildtype (WT) and Δ382 SARS-CoV-2 was performed in this study. High-density RNA-sequencing (RNA-seq) revealed upregulated eIF2 signaling and cellular stress responses, with an under-expression of neutrophil activation-associated signature in Δ382 SARS-CoV-2 infected patients. More robust T and B cell responses were observed, evidenced by enrichment of effector cytotoxic genes and upregulation of SARS-CoV-2 specific T cell immunity and antibody responses.

## Methods

### COVID-19 Patients

A total of 66 patients (WT, *n* = 36 and Δ382, *n* = 30) who tested PCR-positive for SARS-CoV-2 in nasopharyngeal swab samples was recruited into the study from February to April 2020 (Supplemental Table 1). Demographic data, clinical manifestations, and gene deletion status were obtained from patient records throughout hospitalization (Supplemental Table 1). Blood was collected in Cell Preparation Tubes (CPT; BD) from COVID-19 patients at acute and convalescent timepoints. Plasma fraction was extracted from CPT tubes for serology and multiplex microbead-based immunoassay while isolated peripheral blood mononuclear cells (PBMCs) were then used for T cell restimulation analysis. Whole blood samples of COVID-19 patients and healthy controls (Supplemental Table 2) were also collected into Tempus™ Blood RNA Tubes (Applied Biosystems) and stored at − 80 °C for transcriptomic profiling.

### Detection of 382-nt Deletion in SARS-CoV-2 ORF8

Detection of the 382-nt deletion in COVID-19 patients was performed as previously described [11]. Briefly, confirmation of 382-nt deletion in SARS-CoV-2 ORF8 was performed by designing two specific PCR primers flanking the deleted region (F1 primer: 5′-TGTTAGAGGTACAACAGTACTTT-3′, and R1 primer: 5′-GGTAGTAGAAATACCATCTTGGA-3′). A hemi-nested PCR was performed with a different forward primer (F2 primer: 5′-TGTTTATAACACTTTGCTTCACA-3′) and R1 primer for samples with low cycle threshold (Ct) values. PCR products were visualized by gel electrophoresis, and 382-nt deletions were verified by Sanger sequencing.

### RNA Extraction

Whole blood samples of 25 COVID-19 patients and six healthy controls were collected into Tempus™ Blood RNA Tubes (Applied Biosystems) and stored at − 80 °C. Patient samples at acute (SARS-CoV-2 PCR-positive) and recovered (SARS-CoV-2 PCR-negative) stages were selected for RNA extraction. Tempus™ Blood RNA Tubes were heat-inactivated at 60 °C for 30 min according to regulatory requirements, followed by RNA extraction using MagMAX™ for Stabilized Blood Tubes RNA Isolation Kit (Invitrogen) as per manufacturer’s instructions.

### RNA-Sequencing (RNA-Seq)

Purified RNA was analyzed on Bioanalyser (Agilent) for quality assessment. RNA samples with RNA Integrity Number (RIN) of more than 6 were selected for the study (RIN ranging from 6.3 to 9.1 and with a median of 7.5) (Supplemental Table 1). cDNA libraries were prepared by Smart-Seq v2 [21], using a modification of the GlobinLock (GL) method [22] to block transcription of globin mRNA. Human “DNA 3 long A” and “DNA 3 long B” oligonucleotides (0.6 pmol each) were added to 2 ng of total blood RNA in 2.3 μL, denatured at 95 °C for 30 s, incubated at 60 °C for 10 min for GL oligo hybridization, and held at 42 °C for the loading of the reverse transcriptase (RT) mixture. RT and subsequent steps were according to Smart-Seq v2 with the following modifications: (1) addition of 20 μM template switching oligos (TSO) and (2) use of 200 pg cDNA with 1/5 reaction of Nextera XT Kit (Illumina). The length distribution of the cDNA libraries was monitored using a DNA High Sensitivity Reagent Kit on the LabChip (Perkin Elmer). All samples were subjected to an indexed paired-end sequencing run of 2 × 151 cycles on a HiSeq 4000 system (19 samples/lane; Illumina).

### Bioinformatics and Differential Gene Expression Analysis

STAR aligner [23] was used to map paired-end raw reads to human genome build GRCh38 and counted for genes using featureCounts [24] based on GENCODE v31 gene annotation [25]. Log_2_ transformed counts per million mapped reads (log_2_CPM), and log_2_ transformed reads per kilobase per million mapped reads (log_2_RPKM) were computed using the edgeR Bioconductor package [26]. Sequencing coverage statistics are listed in Supplemental Table 3. Data are accessible at NCBI’s Gene Expression Omnibus (GEO) database (GSE155454). Genes with log_2_CPM inter-quartile range (IQR) of less than 0.5 across all samples were filtered out from subsequent differential expression gene (DEG) analysis. Respective DEG analyses for PCR (positive vs. negative), mutation (wildtype (WT) vs. Δ382), and disease severity profiles were done using edgeR [26]. DEG comparison analysis between PCR profiles was done using sample blocking model design by comparing paired samples from the same individual. Multiple testing correction was performed by using a false-discovery rate approach with the Benjamini–Hochberg method [27]. Principal Component Analysis (PCA) was performed on log_2_RPKM values using R function “prcomp.” All DEG analyses and PCA were done in the R statistical language (version 3.3.3) [28].

### Integrative Pathway and Network Analysis

Biological processes, canonical pathways and upstream regulators were predicted from the DEGs with Ingenuity Pathway Analysis (IPA; Qiagen). Gene Ontology (GO) enrichment analysis (including biological processes, cellular component and molecular function categories) for DEGs was performed using Enrichr functional annotation tool [29], with the Fisher’s exact *p*-value set to < 0.01. The smallest *p*-value indicates the highest degree of enrichment. ClueGO (version 2.5.7), a plug-in app of Cytoscape (version 3.8.0; NIGMS; http://www.cytoscape.org/), was used to visualize and explore enriched pathways and biological terms related to DEGs. Heatmaps of log_2_RPKM values for DEGs were generated using ClustVis [30] and the rows are clustered using correlation distance and average linkage.

### Multiplex Microbead-Based Immunoassay

Patient plasma samples were inactivated with Triton™ X-100 (Thermo Fisher Scientific) to a final concentration of 1% for 2 h in the dark. Measurement of immune mediators was done using the Cytokine/Chemokine/Growth Factor 45-plex Human ProcartaPlex™ (Thermo Fisher Scientific) with the Luminex™ assay [17]. Briefly, standards and plasma from COVID-19 patients and healthy controls were incubated with fluorescent-coded magnetic beads pre-coated with respective antibodies in a black 96-well clear-bottom plate overnight at 4 °C. After incubation, plates were washed five times with wash buffer (PBS with 1% BSA (Capricorn Scientific) and 0.01% Tween (Promega)). Sample-antibody-bead complexes were incubated with biotinylated detection antibodies for one hour and washed five times with wash buffer. Subsequently, streptavidin-PE was added and incubated for another 30 min. Plates were washed five times before sample-antibody-bead complexes were re-suspended in sheath fluid for acquisition on the FLEXMAP® 3D (Luminex) using xPONENT® 4.0 (Luminex) software. Data analysis was done on Bio-Plex Manager™ 6.1.1 (Bio-Rad). Standard curves were generated with a 5-PL (5-parameter logistic) algorithm; reporting mean fluorescence intensity (MFI) and concentration data values. The concentrations were logarithmically transformed to ensure normality. The logarithmically transformed value of Limit of Quantification (LOQ) was assigned to samples with concentrations out of the measurement range.

### SARS-CoV-2-Specific T cells by Intracellular Cytokine Staining (ICS)

To profile the SARS-CoV-2 specific T effector subsets in COVID-19 patients, frozen PBMCs from the first convalescent timepoint (median 19.5 days PIO, IQR 16–26) were thawed and rested overnight at 37 °C in RPMI 1640 (Hyclone) supplemented with 5% human serum (Innovative Research), followed by stimulation with phorbol 12-myristate 13-acetate (PMA 100 ng/mL, Sigma Aldrich) and ionomycin (1 µg/mL, Sigma Aldrich), or pooled SARS-CoV-2 PepTivator S, S1, M and N peptides (0.6 nmol/mL each) (Miltenyi Biotec) for 6 h. Brefeldin A and Monesin (1 × , ThermoFisher Scientific) were added at 2 h post-stimulation. Cells were stained for surface markers in the dark at room temperature for 30 min (Supplemental Table 4), followed by fixation and permeabilization for 30 min with Foxp3/Transcription Factor Staining Buffer Set (ThermoFisher Scientific). Permeabilized cells were then stained for intracellular cytokines in the dark at room temperature for 30 min (Supplemental Table 4). Cells were then washed with PBS and centrifuged at 800 × g for 5 min before transferring to respective polystyrene FACS tubes containing 5 μL (5.4 × 10^3^ beads) of CountBright Absolute Counting Beads (Invitrogen). Cells were acquired with the Cytek Aurora cytometer (Cytek Biosciences) and analyzed using FlowJo (Tree Star).

### Serology Profiling

Antibody response against the full-length SARS-CoV-2 spike protein was examined using an S protein flow-based (SFB) assay [31]. Cells expressing full-length SARS-CoV-2 spike protein were seeded at 1.5 × 10^5^ cells per well in 96 well plates (Thermo Fisher Scientific). The cells were first incubated with plasma samples from COVID-19 patients and healthy controls (1:100 dilution in 10% fetal bovine serum, FBS), followed by secondary incubation with a double stain, consisting of Alexa Fluor 647-conjugated anti-human IgM or IgG (Thermo Fisher Scientific; 1:500 dilution in 10% FBS) and propidium iodide (Sigma-Aldrich; 1:2500 dilution). Cells were acquired on LSRII 4 Laser flow cytometer (BD Biosciences) and analyzed using FlowJo (Tree Star). A positive antibody response cut-off is defined as the healthy controls’ mean + 3 standard deviations (SD).

To further define the antibody response against the SARS-CoV-2 spike protein, a peptide-based ELISA was conducted against two epitopes, S14P5 and S21P2, to determine patients’ IgG antibody response against SARS-CoV-2 [32, 33]. Briefly, 50 μL of 0.5 μg/mL of NeutrAvidin protein (Thermo Fisher Scientific) was coated on Nunc Maxisorp flat-bottom 96-well plates (Thermo Fisher Scientific) overnight at 4 °C. Blocking was done with 0.01% polyvinyl alcohol (PVA; Sigma-Aldrich) in PBS containing 0.1% Tween-20 (0.1% PBST; Sigma-Aldrich). Biotinylated peptides (GenScript; 1:2000 dilution in 0.1% PBST) were added to wells, followed by 1% Triton X-100-inactivated plasma samples from COVID-19 patients and healthy controls (1:1000 dilution in 0.1% PBST), and horseradish peroxidase (HRP)-conjugated goat anti-human IgG (H + L) (Jackson ImmunoResearch) in blocking buffer. For color development, 3,3′,5,5′-tetramethylbenzidine (TMB; Sigma-Aldrich) was added for 5 min, and the reaction stopped with 0.16 M sulfuric acid (Merck). Absorbance was measured on an Infinite M200 plate reader (Tecan) at 450 nm and a reference wavelength of 690 nm. Before subtracting background signals, raw optical density (OD) values were normalized to a positive control to account for plate-to-plate variations. Incubation was done at 37 °C for one hour in between steps.

### Data and Statistical Analyses

Data analyses were done using GraphPad Prism (GraphPad Software, version 8.4.3). Mann–Whitney *U* tests were conducted on the logarithmically transformed concentration of immune mediators. Comparison of SARS-CoV-2 specific T cell responses and serological profiles between WT- and Δ382-infected patients were analyzed by Mann–Whitney *U* tests. A cut-off value of mean + 3SD of healthy controls was used as a baseline to classify the serological profile of COVID-19 patients as positive or negative [32, 33].

## Results

### Wildtype and Δ382 SARS-CoV-2 Infections Activate TLR and PRR Pathways and Antiviral Interferon Responses in COVID-19 Patients

We herein studied 30 Δ382 SARS-CoV-2 infected patients and compared their transcriptomic signatures, systemic soluble immune mediator levels, and adaptive immune responses against 36 WT infected patients (Supplemental Table 1). To uncover the molecular mechanisms underlying the milder disease phenotype in Δ382 SARS-CoV-2 infections [17], RNA-seq of whole blood from 25 COVID-19 patients was performed (WT, *n* = 14 and Δ382, *n* = 11) in this study (Supplemental Table 1).

**Fig. 1:**
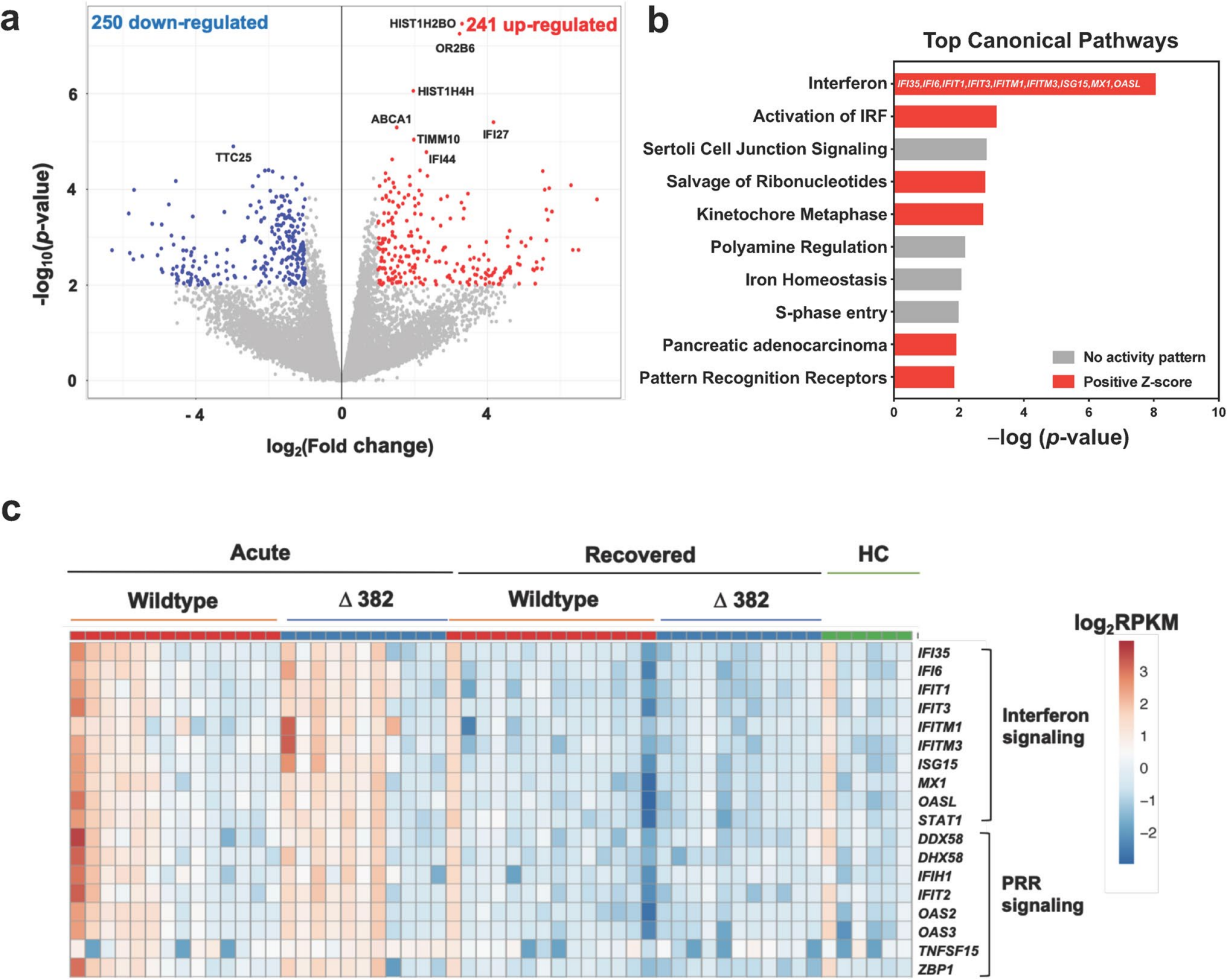
Whole blood transcriptome analysis in COVID-19 patients. RNA-seq of whole blood from COVID-19 patients (*n* = 25) at acute (SARS-CoV-2 PCR-positive, median 8 days PIO) and recovered (SARS-CoV-2 PCR-negative, median 21 days PIO) phases and healthy controls (*n* = 6) was performed. **a** Volcano plot indicating DEGs between blood samples collected at acute and recovered phases in patients with WT SARS-CoV-2 infection (*n* = 13), with thresholds of *p*-value < 0.01 and |FC|> 2. Numbers of over-expressed and under-expressed genes are indicated. **b** Top IPA canonical pathways showing differential expression of genes related to IFN and PRR signaling in WT SARS-CoV-2 infection. Pathways are ranked by − log(*p*-value), and the color scheme is based on predicted activation Z-scores, with activation in red and undetermined directionality in gray. DEGs related to the IFN pathway are indicated on the bar graph. **c** Heatmap of DEGs related to IFN and PRR signaling between COVID-19 patients infected with WT (*n* = 14) and Δ382 SARS-CoV-2 (*n* = 11) at acute and recovered phases and healthy controls. Heatmap is scaled based on log_2_RPKM values, with blue and red indicating low and high expressions, respectively. WT, wildtype; DEGs, differentially expressed genes; FC, fold change; FDR, false discovery rate; PCR, polymerase chain reaction; PIO, post-illness onset; HC, healthy controls; RPKM, reads per kilobase per million reads mapped; IFN, interferon; PRR, pattern recognition receptor

Firstly, a comparison of transcriptomic profiles during the acute (SARS-CoV-2 PCR-positive, median 8 days post-illness onset [PIO]) and recovered (SARS-CoV-2 PCR-negative, median 21 days PIO) phases of disease was performed on 13 WT SARS-CoV-2 infected patients with paired samples to identify transcriptomic changes specific to virus infection. Following correction for multiple testing (*q*-value ≤ 0.05 and |FC| > 2), only eight genes remained significant, as highlighted in the volcano plot (Fig. 1a). To gain an understanding of the underlying mechanisms, less stringent criteria of *p* < 0.01 and |FC|> 2 was applied to pathway and Gene Ontology (GO) analyses [34]. This yielded 491 significant transcripts, in which 241 differentially expressed genes (DEGs) were found to be enriched, whereas 250 were downregulated (Fig. 1a, Supplemental Table 5) during the acute phase of infection.

To elucidate the biological processes and pathways of the involved DEGs, further analyses were performed using GO enrichment and Ingenuity Pathway Analysis (IPA) on the dataset of 491 DEGs. Gene functional enrichment analysis revealed that robust type I IFN, classical complement, and humoral and cellular immune responses were induced, whereas biological processes such as “target of rapamycin (TOR) signaling,” “protein catabolic process,” and “positive regulation of autophagy” were shown to be downregulated in the acute phase of WT SARS-CoV-2 infection (Supplemental Fig. 1). IPA analysis revealed IFN signaling, IFN regulatory factor (IRF) activation, pattern recognition receptors (PRR)-induced signaling and salvage pathway of ribonucleotides as the top canonical pathways induced upon SARS-CoV-2 infection (Fig. 1b). Overall, the results showed that antiviral IFN responses and virus sensing PRR signaling pathways were activated following WT SARS-CoV-2 infection (Fig. 1c). A differential response observed among patients in both WT and Δ382 SARS-CoV-2 groups might be explained by the heterogeneity of disease progression in these patients, whereby those with a more severe disease outcome demonstrated more robust type I interferon responses [35, 36].

### Δ382 SARS-CoV-2 Infection Upregulates eIF2 Signaling and Cellular Stress Responses in COVID-19 Patients

**Fig. 2:**
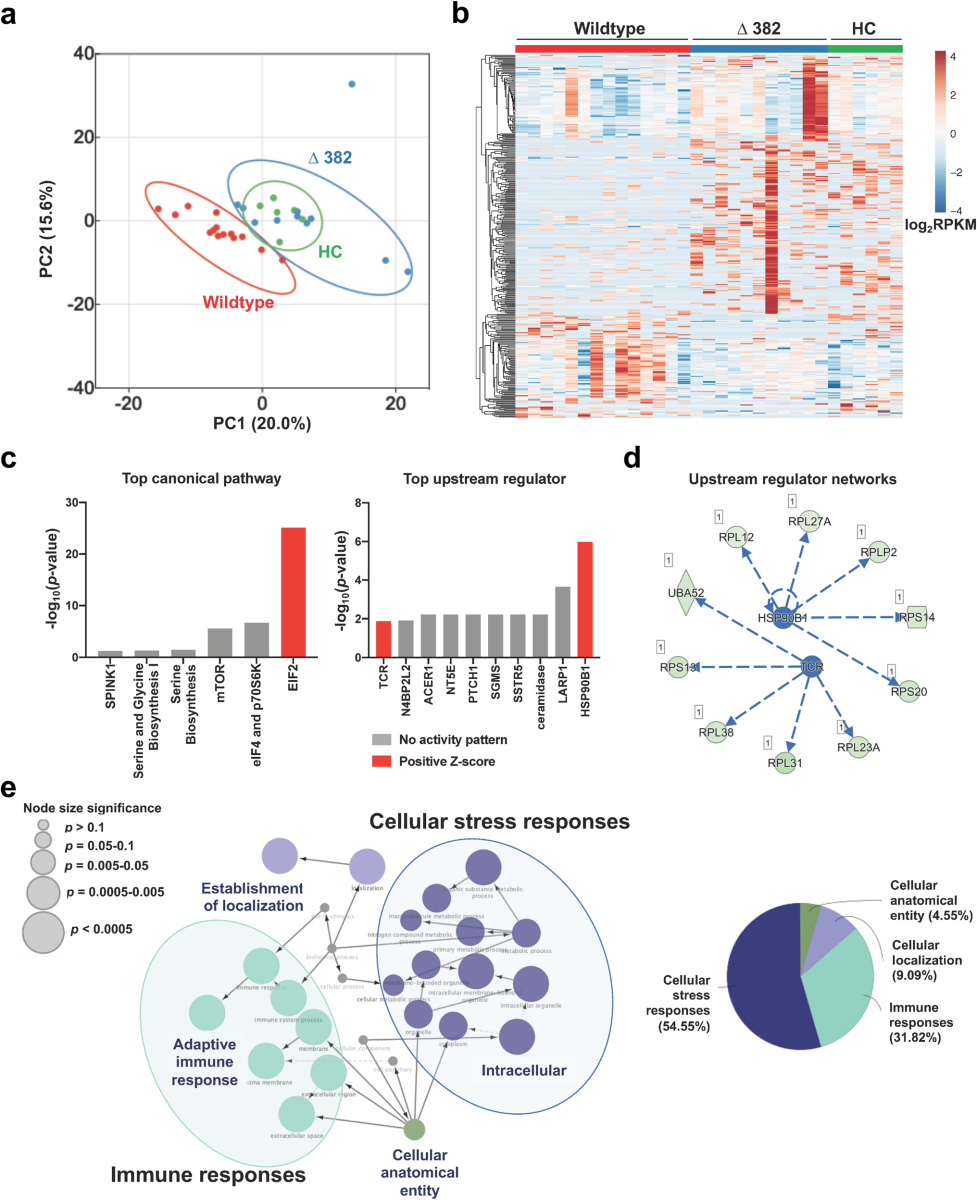
Effects of 382-nt deletion in SARS-CoV-2 ORF8 genome (Δ382) on whole blood transcriptome of COVID-19 patients. RNA-seq of whole blood from COVID-19 patients infected with WT (*n* = 14) and Δ382 SARS-CoV-2 (*n* = 11) at the acute phase of infection (SARS-CoV-2 PCR-positive; median 8 days PIO) was performed. Only samples with RNA integrity number > 6 were sent for sequencing and included in the analysis **a** PCA of COVID-19 patients and healthy controls based on DEGs, with *p*-value < 0.01 and |FC|> 2. **b** Heatmap of 358 DEGs, scaled based on log_2_RPKM values, with blue and red colors indicating low and high expressions, respectively. **c** Top canonical pathways and upstream regulators identified by IPA based on the DEGs. Bar graphs are ranked by significance, with red indicating positive predicted activation Z-scores and gray indicating undetermined directionality. **d** An integrated network of HSP90B1 and TCR and their targeted genes. Stimulation of HSP90B1 and TCR leads to overexpression of the downstream genes. **e** GO pathway term enrichment networks of DEGs using Cytoscape add-on ClueGO. Each of the GO terms is statistically significant (Benjamini–Hochberg correction < 0.05). The filled colored circles (nodes) represent a statistically significant enriched parent GO term. The lines (edges) between nodes show overlapping genes within terms, with node size representing the term enrichment significance. The overview chart shows the distribution of the functionally grouped GO terms. The cut-off for terms in the functionally grouped networks was set at *p*-value < 0.05. WT, wildtype; PCA, principal component analysis; DEGs, differentially expressed genes; FC, fold change; RPKM, reads per kilobase per million reads mapped; PCR, polymerase chain reaction; IPA, Ingenuity Pathway Analysis; HSP90B1, heat shock protein 90 kDa beta member 1; TCR, T cell receptor; GO, gene ontology

A comparative analysis was next carried out to identify specific differences in the whole blood transcriptomes of patients during the acute phase of WT (median 9.5 days PIO, *n* = 14) and Δ382 SARS-CoV-2 infection (median 6 days PIO, *n* = 11), as well as healthy controls (*n* = 6; Supplemental Table 2). With the cut-off criteria of *p*-value < 0.01 and |FC|> 2 [34], 358 DEGs were identified, in which 259 were over-expressed, and 99 were under-expressed in Δ382 SARS-CoV-2 infected patients (Supplemental Table 6). Principal component analysis (PCA) showed a clear distinction between the two groups of patients, while Δ382 SARS-CoV-2 infected patients overlapped with healthy controls (Fig. 2a). Notably, gene expression signatures comprising significant DEGs illustrated contrasting whole blood transcriptomic responses between WT and Δ382 SARS-CoV-2 infections, with the profile of Δ382 SARS-CoV-2 being closer to that of the healthy uninfected controls (Fig. 2b). Further analysis of the 358 DEGs revealed eight significant canonical signaling pathways, with the eukaryotic initiation factor 2 (eIF2) signaling pathway being the most significantly enriched in Δ382 SARS-CoV-2 infection (Fig. 2c).

In addition, Heat Shock Protein 90 Beta Family Member 1 (HSP90B1) and T Cell Receptor (TCR) were predicted to be highly stimulated in Δ382 SARS-CoV-2 infected patients compared to WT patients (Fig. 2c). Stimulation of both HSP90B1 and TCR formed a regulatory network involving ribosomal stress-induced related genes (Fig. 2d). Moreover, a network‑based analysis of GO terms (biological, cellular, and molecular processes) using ClueGO (plugged into Cytoscape) showed that cellular stress and immune responses (54.55% and 31.82%, respectively) were significantly enriched in the transcriptomic profiles of Δ382 SARS-CoV-2 infected patients, as compared to WT patients (Fig. 2e).

### Infection with Δ382 SARS-CoV-2 Is Associated with Lower Activation of Neutrophils and More Robust T cell Immunity

**Fig. 3:**
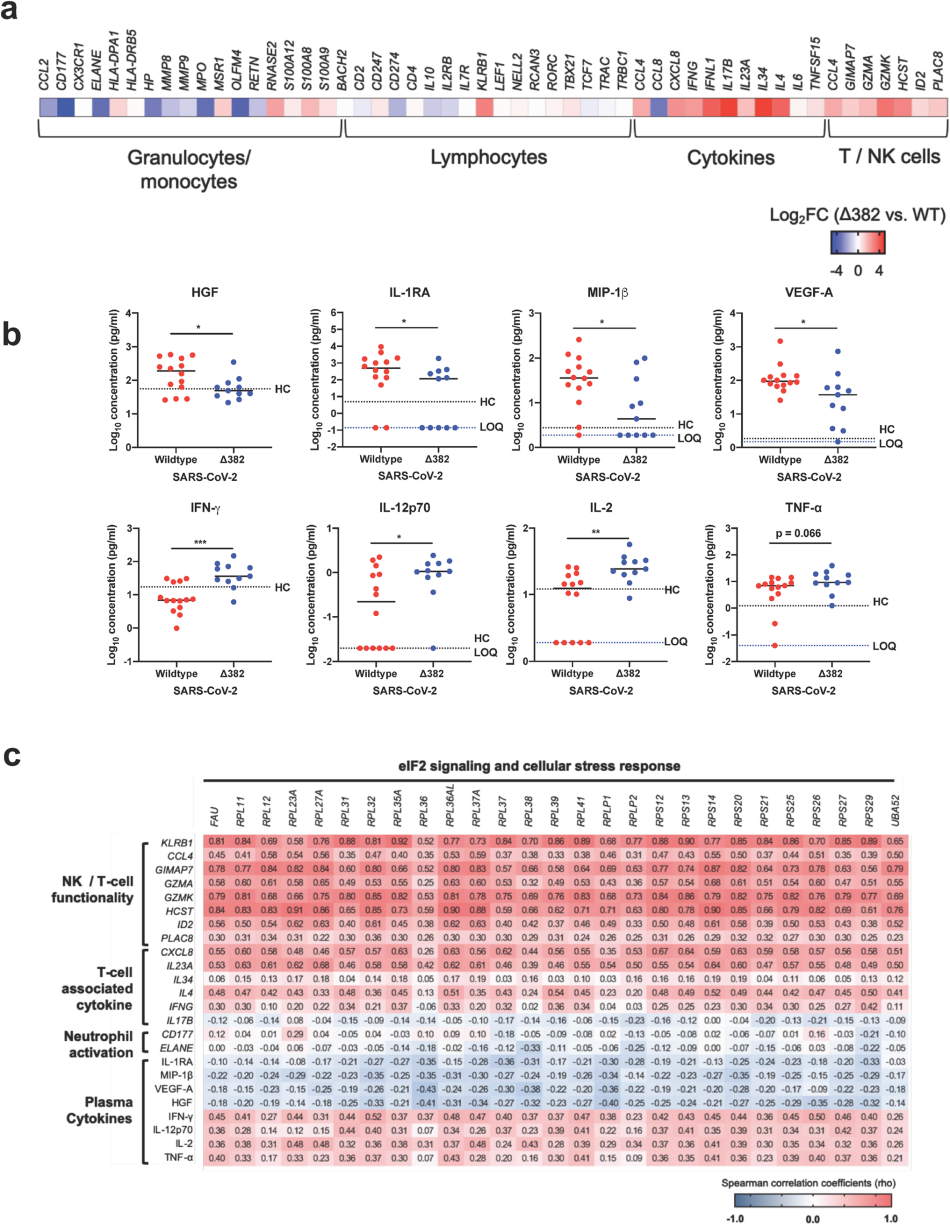
Effects of 382-nt deletion in SARS-CoV-2 ORF8 genome on immune responses in COVID-19 patients. Transcriptomic and cytokine profiles of COVID-19 patients infected with WT (*n* = 14) or Δ382 SARS-CoV-2 (*n* = 11) at the acute phase of infection (SARS-CoV-2 PCR-positive; median 8 days PIO) were analyzed. **a** Expressions of genes associated with granulocytes, monocytes, lymphocytes, cytokines and T/NK cell functionality were compared between COVID-19 patients infected with WT or Δ382 SARS-CoV-2. Heatmaps of the DEGs, scaled based on log_2_FC values, with blue and red colors indicating low and high expressions, respectively. **b** Plasma immune mediator levels of COVID-19 patients infected with WT (*n* = 14) or Δ382 SARS-CoV-2 (*n* = 11) at the acute phase of infection (SARS-CoV-2 PCR-positive; median 8 days PIO) and profiles of significant immune mediators are illustrated as scatter plots and shown as mean. Mann–Whitney *U* tests were conducted on the logarithmically transformed concentration values (*p < 0.05; **p < 0.01; ***p < 0.001). Respective mean concentrations of immune mediators from healthy controls (HC; *n* = 23) are indicated as black dotted lines. Patient samples with concentrations out of measurement range are presented as the logarithmically transformed value of LOQ and indicated as blue dotted lines. **c** Association between elF2 signaling and immune signatures in Δ382 SARS-CoV-2 infection. Spearman’s correlation matrix for the genes associated with eIF2 signaling, T cell functionality, neutrophil activation and plasma cytokines. Colors represent the Spearman correlation coefficients (rho) between the expression of genes related to eIF2 signaling and genes or immune mediators associated with different immune phenotypes. FC, fold change, WT, wildtype; PCR, polymerase chain reaction; PIO, post-illness onset; DEGs, differentially expressed genes; LOQ, limit of quantification

Next, previously reported DEGs in the whole blood transcriptomes of COVID-19 patients [37, 38] were compared against the WT (*n* = 14) and Δ382 SARS-CoV-2 (*n* = 11) infected patients in our study. Increased gene expression was found for granulocyte- and monocyte-associated molecules (eosinophil-derived neurotoxin; *RNASE2*) and lymphocyte-associated molecules (natural killer cell surface protein P1A; *KLRB1*) in Δ382 SARS-CoV-2 infected patients (Fig. 3a). Notably, lower expressions of neutrophil activation-associated CD177 and neutrophil elastase (*ELANE*) were observed in Δ382 SARS-CoV-2 infected patients (Fig. 3a). Moreover, a higher expression of T cell cytokine genes (*CCL4, CXCL8, IFNG, IL17B, IL23A, IL34*) and genes associated with T/NK cell functionality (*CCL4, CIMAP7, GZMA, GZMK, HCST, ID2, PLAC8*) was also observed in Δ382 SARS-CoV-2 infected patients (Fig. 3a).

In addition to the increased expression of T cell activation associated genes, quantified acute plasma immune mediator levels in patients also showed higher levels of T cell-associated cytokines (IFN-γ, IL-12p70, IL-2, and TNF-α), with lower levels of chemokines (MIP-1β) and disease severity associated cytokines (HGF, IL-1RA, and VEGF-A) [39] (Fig. 3b and Supplemental Table 7). Interestingly, correlation analysis also showed that the levels of genes related to T cell functionality and neutrophil activation, together with T cell-associated and pro-inflammatory immune mediators, are associated with expression levels of genes related to cellular stress responses and eIF2 signaling (Fig. 3c). Increased activation of eIF2 signaling is associated with enhanced T cell function and reduced inflammatory response, and neutrophil activation.

### Δ382 SARS-CoV-2 Infected Patients Have more Robust Virus-Specific Adaptive Immune Responses

**Fig. 4:**
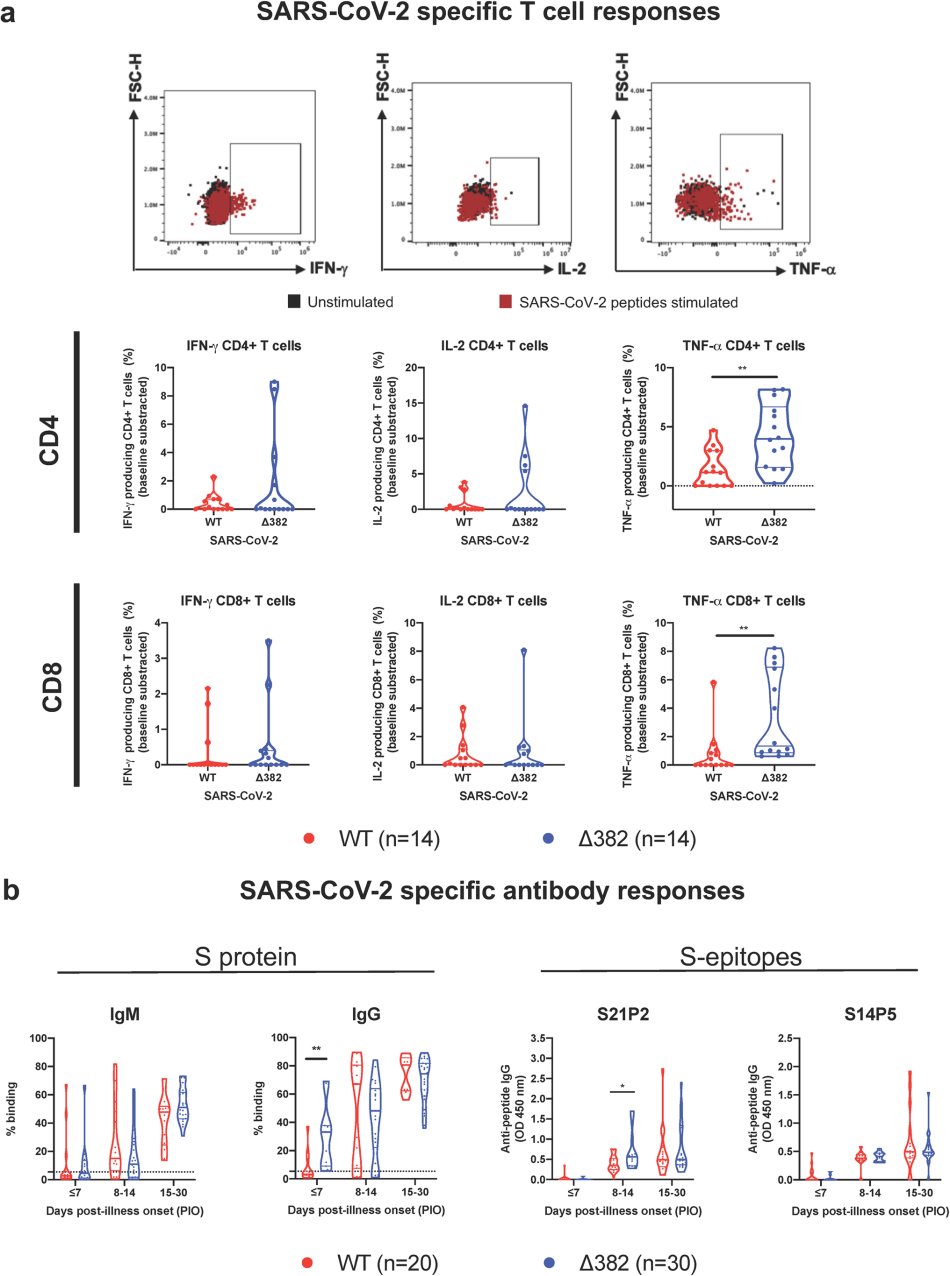
Effects of 382-nt deletion in SARS-CoV-2 ORF8 genome on adaptive immune responses to COVID-19. **a** SARS-CoV-2 specific CD4^+^ and CD8^+^ non-T follicular helper (TFH) cells were characterized with flow cytometry-based on the expression of IFN-γ, IL-2, and TNF-⍺ upon SARS-CoV-2 peptide stimulation in WT (*n* = 14) or Δ382 SARS-CoV-2 (*n* = 14) infected patients. Statistical analyses were performed with the Mann–Whitney *U* test (**p < 0.01). **b** Antibody responses in Δ382 SARS-CoV-2 infected patients. Spike protein-specific antibody response was characterized using an S-flow assay. Plasma samples of COVID-19 patients infected with either WT (*n* = 20) or Δ382 SARS-CoV-2 (*n* = 30) were screened at 1:100 dilution against cells expressing the full-length SARS-CoV-2 spike protein, with healthy donors (*n* = 22) screened in parallel. IgM and IgG profiles of COVID-19 patients at timepoints ≤ 7, 8 to 14, and 15 to 30 days PIO are illustrated as violin plots. The dotted line indicates the mean + 3SD of healthy donors. Data are shown as mean ± SD of two independent experiments. For determination of anti-peptide IgG responses, plasma samples o COVID-19 patients infected with either WT (*n* = 20) or Δ382 SARS-CoV-2 (*n* = 30) were tested at 1:1000 dilution on an IgG ELISA against SARS-CoV-2 spike epitopes S21P2 and S14P5. Healthy controls (*n* = 22) were screened in parallel. Antibody profiles of COVID-19 patients at timepoints ≤ 7, 8 to 14, 15 to 30 days PIO are illustrated as violin plots. The dotted line indicates the mean + 3SD of healthy controls. Data are shown as mean ± SD of two independent experiments. Statistical analysis was carried out using Mann–Whitney *U* tests (*p < 0.05). WT, wildtype

To further assess the difference in T cell responses, PBMCs of 28 patients collected shortly after acute infection (median 19.5 days PIO, IQR 16–26) were stimulated with pooled pan-SARS-CoV-2 peptides or with PMA/ionomycin [40]. Intracellular cytokine staining demonstrated virus-specific CD4^+^ and CD8^+^ T cells responses in these patients. Δ382 SARS-CoV-2 infected patients had higher CD4^+^ and CD8^+^ T cells expressing TNF-α than WT infected patients following peptide stimulation (Fig. 4a, Supplemental Fig. 2), indicating a more robust SARS-CoV-2 specific T cell response in patients with mutant virus infection. Although not significant, they also had higher IL-2 and IFN-γ expressing virus-specific CD4^+^ and CD8^+^ T cells. Intriguingly, the general T_h__1_ responses following PMA/ionomycin stimulation were higher in WT infection compared to Δ382 SARS-CoV-2 infection (Supplemental Fig. 3). WT infected patients also showed higher IL-17^+^ CD4^+^ T cells but with lower IL-4^+^/IL-6^+^ CD4^+^ T cells compared to patients with mutant virus infection.

In concordance with robust SARS-CoV-2 specific T cell response, further characterization of humoral response with the S-flow assay [41] revealed a higher level of detectable IgG response against the SARS-CoV-2 spike protein in Δ382 SARS-CoV-2 patients (*n* = 30) compared to WT infected patients (*n* = 20) at the acute phase of infection (< 7 days PIO). However, this phenomenon was not observed at later time points (Fig. 4b). In addition, a peptide-based ELISA against two previously identified immunodominant B-cell linear epitopes, S21P2 and S14P5 on the spike glycoprotein [32, 33], revealed that the IgG levels against S21P2 were significantly higher in Δ382 SARS-CoV-2 infected patients at the later PIO) (Fig. 4b).

## Discussion

Understanding the impact of virus mutation on the pathophysiology of COVID-19 is imperative to finding new treatments and providing essential clues on its potential impact on transmission rates and disease severity. Here, we analyzed in detail the peripheral blood transcriptomes and adaptive immune responses of COVID-19 patients with Δ382 SARS-CoV-2 infection and elucidated several key immune responses potentially associated with the deletion of SARS-CoV-2 ORF8.

**Fig. 5:**
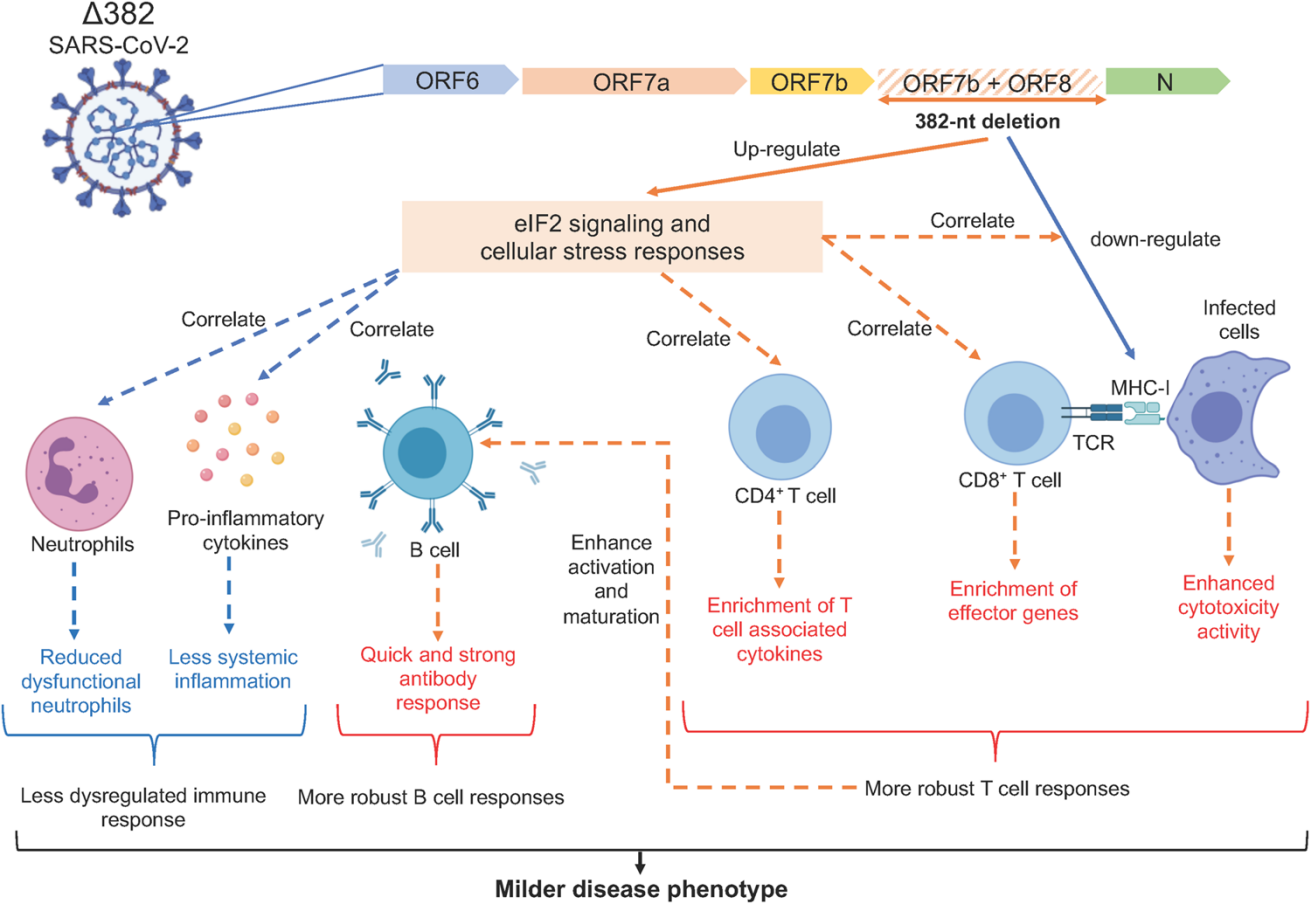
Molecular mechanisms underlying the milder disease phenotype in Δ382 SARS-CoV-2 infections. A SARS-CoV-2 variant with a 382-nucleotide deletion (Δ382) truncates ORF7b and removes the ORF8 transcription-regulatory sequence, eliminating ORF8 transcription. The ORF8 382-nt deletion has recently been associated with a milder disease phenotype. The attenuation of SARS-CoV-2 ORF8 upregulates eIF2 signaling and cellular stress responses at the acute phase of infection, potentially interrupting the downregulation of MHC-I molecules by ORF8 and also enhances the activation of both CD4^+^ and CD8^+^ T cells, evidenced by enrichment of effector cytotoxic genes and upregulation of SARS-CoV-2 specific T cell responses in Δ382 SARS-CoV-2 infected patients. Enhanced T cell responses may in turn mediate rapid and effective antibody responses in Δ382 SARS-CoV-2 infection. More pronounced cellular stress responses may further reduce systemic inflammation and dysfunctional neutrophils in Δ382 SARS-CoV-2 infected patients. Overall, the attenuation of SARS-CoV-2 ORF8 produced a molecular phenotype characterized by more pronounced cellular stress responses and a less dysregulated immune phenotype with more robust T and B cell responses. ORF, open reading frame; eIF2, eukaryotic initiation factor 2; MHC-I, major histocompatibility complex 1; CD, cluster of differentiation

This study reveals distinctly different transcriptomic profiles between WT and Δ382 SARS-CoV-2 infections, with the Δ382 eliciting a more active cellular stress response and an upregulated eIF2 signaling in the infected patients. Studies have demonstrated that coronaviruses induce cellular stress responses following infection [42–44] by targeting unfolded protein response (UPR) pathways to cause an imbalance in cellular homeostasis, which subsequently induces endoplasmic reticulum (ER) stress that favors viral replication [45]. While SARS-CoV ORF8 protein has been previously reported to induce ER stress by specifically targeting activating transcription factor 6 (ATF6) [46], our results suggest that the SARS-CoV-2 ORF8 protein potentially triggers protein kinase RNA-like ER kinase (PERK) and eIF2 signaling mechanisms, further strengthening an earlier report showing interactions between SARS-CoV-2 ORF8 protein and human proteins involved in ER quality control [47]. ER stress is known to propagate inflammatory responses [48, 49], which is consistent with our findings showing reduced systemic inflammation and ER stress levels in Δ382 SARS-CoV-2 infected patients (Fig. 5). Notably, there is still controversy amongst the literature about the susceptibility of human PBMCs to SARS-CoV-2 [50, 51]. Although SARS-CoV-2 was detected in immune cells of COVID-19 patients [51–53], it remains to be determined whether enhanced activation of the eIF2 pathway in Δ382 SARS-CoV-2 infected patients is directly caused by a loss of ORF8’s impact on ER quality control or due to secondary peripheral immune responses to the mutant virus infection, mediated by soluble factors.

SARS-CoV-2 ORF8 has also been reported to interact with MHC-I molecules [20] and subsequently downregulate cytotoxic functions of T lymphocytes. Cytotoxic effector genes (*GZMA*, *GZMB*, *ID2*, and *PLAC8*) were enriched in Δ382 SARS-CoV-2 infected patients, coupled with high plasma levels of IFN-γ, TNF-α, and IL-2 during the acute phase of virus infection. In addition to robust cytotoxic T cell functions, enrichment of *KLRB1*, *GZMA*, and *GZMK* transcripts may indicate enhanced NK cell cytotoxic activity in Δ382 SARS-CoV-2 infected patients, in which the function is impaired in severe COVID-19 patients [54]. Our findings are in agreement with other single-cell studies reporting an enrichment of effector populations with a cytotoxic phenotype (effector CD8^+^, MAIT and NK T cells) in COVID-19 individuals with milder disease phenotype [55, 56] and further highlight the impact of SARS-CoV-2 ORF8 on cytotoxic cellular responses in COVID-19 (Fig. 5). A higher magnitude of virus-specific T cells was induced following Δ382 infection, and this is in line with other studies in which highly functional virus-specific cellular immune response resulted in better disease outcomes in COVID-19 [57, 58]. It is important to note that genetic variability of the cohort could also explain some of the differences in the T cell response since a few human leukocyte antigen (HLA) alleles, which are predominant in Asia, are associated with COVID-19 severity [59, 60]. Additionally, enhanced effector functions of virus-specific T cells may in turn mediate rapid and protective antibody responses against SARS-CoV-2 infection [61]. Concordantly, higher IgG responses during the early phase of disease were observed in Δ382 SARS-CoV-2 infected patients, which could indicate a more robust CD4^+^ T cell response driving B cell activation and maturation in these patients [62, 63] (Fig. 5). Thus, deletion of ORF8 could result in increased immunogenicity against SARS-CoV-2. Intriguingly, while IgG levels at the later phase of infection have been associated with severe COVID-19 [33, 64], Δ382-infected patients with a milder disease phenotype in this report had higher IgG levels at the early acute phase of infection. Our observations are consistent with the findings, which found that S-specific antibody responses were elevated early in COVID-19 individuals who recovered from the disease compared to deceased patients [65]. Further work to fully define the exact roles of IgG in SARS-CoV-2 infection will bring additional insights into this phenomenon.

The increased effectiveness of the virus-specific adaptive T and B cell responses may explain the reduced need for sustained, pathogenic pro-inflammatory responses. Δ382 SARS-CoV-2 infected patients had lower pro-inflammatory cytokines, chemokines and growth factors strongly associated with severe COVID-19 [66, 67]. The N protein of SARS-CoV-2 was reported to promote inflammation by increasing IL-6 levels following virus infection [68]. Meanwhile, we did not observe any significant difference in the IL-6 levels between the WT and Δ382 infected patients [17], suggesting differential roles of ORF8 in inducing hyperinflammation in COVID-19. More interestingly, general pro-inflammatory T_h__1_ responses were more robust in WT infected patients. The non-specific and uncontrolled activation of CD4^+^ T cells maybe the cause and effect of heightened inflammation observed in WT infection. Lymphopenia and dysregulated myelopoiesis with immature and dysfunctional neutrophils have also been associated with COVID-19 severity [66, 69–72]. While lymphocyte and neutrophil counts of Δ382 SARS-CoV-2 infected patients did not significantly differ from WT infected patients [17] (Supplemental Table 1), Δ382 infected patients demonstrated molecular signatures characterized by reduced dysfunctional neutrophils and more robust T cell responses (Fig. 5).

Similar to other coronaviruses [73, 74], SARS-CoV-2 activates cellular viral sensors, host defenses and IFN responses during an active virus infection. Despite previous reports showing SARS-CoV-2 ORF8 as a potent IFN antagonist [75], there were no significant differences in the host innate antiviral responses between WT and Δ382 groups in this study (Fig. 1c). This indicates potential functional compensation for the loss of ORF8 by other potent interferon antagonists such ORF3b and ORF6 [75] in the Δ382 SARS-CoV-2 infections.

While mutations on spike protein in SARS-CoV-2 resulted in more efficient virus transmission and severe disease outcomes [76–78], the ORF8 deletion does not appear to increase viral load in patients [11] and is associated with milder disease [17]. While in vitro infection of Δ382 in human nasal epithelial cells induced similar inflammatory signatures [79], it was not clear how the immune profiles in patients would be affected with the absence of ORF8. Nonetheless, putting all the data together, it is clear that the disease phenotypic difference is likely due to the functional implication of ORF8 on the host immune system. Δ382 SARS-CoV-2 viruses likely stimulate the activation of common host response signaling mechanisms similar to WT viruses, but with different degrees of magnitude. Our key findings of the functional implication of ORF8 on host immune surveillance further define the relevance of inhibiting ORF8 function as a possible target for therapeutic intervention in COVID-19. Antiviral drugs can be developed against the SARS-CoV-2 ORF8 protein. However, the hypervariable nature of the ORF8 gene and the rapid evolution it undergoes can compromise the suitability of the ORF8 protein as an antiviral target. Alternatively, host-directed strategies [47] can be developed to target the host factors to which ORF8 establishes critical interactions. In this case, a drug that can modulate ER stress responses may benefit the outcome of COVID-19 [80]. Interestingly, the VOCs that have become dominant as the pandemic progresses bear the ORF8 mutation together with multiple spike mutations. The asymptomatic or mild disease phenotype attributed to the absence of ORF8 and the enhanced infectivity caused by the spike mutations may explain the high transmissibility of these VOCs. Monitoring ORF8 mutation throughout the progression of the COVID-19 pandemic is therefore important, particularly when associated with relevant spike mutations.

Our study has caveats that should be noted. Due to Singapore’s strict and efficient public health measures during the pandemic in 2020, the cases of Δ382 SARS-CoV-2 infections were kept to a low number. As such, we only had a limited number of patients with the mutant virus infection in this study. In addition, host genetic background and differences in variables such as sex and comorbidities between the WT and Δ382 groups, although not significant (Supplemental Table 1), may be confounding in this study. Nevertheless, our observations provide exciting insights into the potential impacts of ORF8 deletion on human immune responses during SARS-CoV-2 infection, and this well-characterized dataset would value-add to the current COVID-19 resource. Future studies with engineered viruses and animal models could further address the interactions and mechanisms between ORF8 and ER stress or T cell responses during COVID-19.

## Supplementary Information

Below is the link to the electronic supplementary material.

Supplementary file1 Supplemental Fig. 1 GO-term enrichment of DEGs in whole blood of COVID-19 patients infected with WT SARS-CoV-2 during the acute phase of infection (SARS-CoV-2 PCR-positive; median 8 days PIO, n=13). GO-term functional enrichments for biological process, molecular function, and cellular component were performed for both up-regulated and down-regulated genes. Pathways are ranked by –log10(*p*-value). GO, gene ontology; DEGs, differentially expressed genes; WT, wildtype; PCR, polymerase chain reaction; PIO, post-illness onset. Supplemental Fig. 2 Representative gating strategy for the characterization of IFN-γ, IL-2, TNF-⍺ expression of CD4^+^ and CD8^+^ T cells in isolated PBMCs of COVID-19 patients by flow cytometry upon peptide stimulation. Supplemental Fig. 3 WT SARS-CoV-2 infected patients exhibit a higher systemic non-specific T_h1_ response, compared to Δ382 SARS-CoV-2 infected patients. CD4^+ ^non T follicular helper (TFH) cells were characterized based on the expression of IFN-γ, IL-2, TNF-⍺, IL-17A, IL-4, IL-6 and IL-10 upon PMA/ Ionomycin stimulation. Statistical analyses were performed with unpaired test (*p < 0.05; **p < 0.01; ***p < 0.001).WT, wildtype. (PDF 2108 KB)

## Data Availability

RNA-seq data are accessible at NCBI’s Gene Expression Omnibus (GEO) database (GSE155454). All data are available upon request: Lisa F.P. Ng, lisa_ng@IDLabs.a-star.edu.sg.
