# Supplementary material for: Robust Virus-Specific Adaptive Immunity in COVID-19 Patients with SARS-CoV-2 Δ382 Variant Infection
Source: J Clin Immunol. 2021 Oct 30;42(2):214–29. doi: 10.1007/s10875-021-01142-z (PMC8556776; doi:10.1007/s10875-021-01142-z)
Supplement: Supplementary file 1 — Supplementary file1 Supplemental Fig. 1 GO-term enrichment of DEGs in whole blood of COVID-19 patients infected with WT SARS-CoV-2 during the acute phase of infection (SARS-CoV-2 PCR-positive; median 8 days PIO, n=13). GO-term functional enrichments for biological process, molecular function, and cellular component were performed for both up-regulated and down-regulated genes. Pathways are ranked by –log10(p-value). GO, gene ontology; DEGs, differentially expressed genes; WT, wildtype; PCR, polymerase chain reaction; PIO, post-illness onset. Supplemental Fig. 2 Representative gating strategy for the characterization of IFN-γ, IL-2, TNF-⍺ expression of CD4+ and CD8+ T cells in isolated PBMCs of COVID-19 patients by flow cytometry upon peptide stimulation. Supplemental Fig. 3 WT SARS-CoV-2 infected patients exhibit a higher systemic non-specific Th1 response, compared to Δ382 SARS-CoV-2 infected patients. CD4+ non T follicular helper (TFH) cells were characterized based on the expression of IFN-γ, IL-2, TNF-⍺, IL-17A, IL-4, IL-6 and IL-10 upon PMA/ Ionomycin stimulation. Statistical analyses were performed with unpaired test (*p < 0.05; **p < 0.01; ***p < 0.001).WT, wildtype. (PDF 2108 KB) [file 10875_2021_1142_MOESM1_ESM.pdf]

Supplemental Table 1 Demographic and clinical data of the COVID-19 patients in multiple analyses.

	**All patients**			**Transcriptomic and Systemic cytokine profiling**			**T cell restimulation analysis**			**Antibody profiling (S-flow and peptide ELISA)**		
**N**	**66**			**25**			**28**			**50**		
	**WT (n=36)**	**Δ382 (n=30)**	***p* value**	**WT (n=14)**	**Δ382 (n=11)**	***p* value**	**WT (n=14)**	**Δ382(n=14)**	***p* value**	**WT (n=20)**	**Δ382 (n=30)**	***p* value**
**Demographics**												
Mean age, years	45 (13)	40 (12)	0.0925	48 (16)	41 (11)	0.2207	42 (12)	43 (13)	0.8834	42 (11)	41 (12)	0.7885
Sex, male	19 (42.9%)	18 (72.7%)	0.6233	6 (42.9%)	8 (72.7%)	0.2270	9 (64.3%)	9 (64.3%)	> 0.999	10 (50.0%)	19 (63.3%)	0.3927
**Ethnicity**												
Chinese	31 (86.1%)	23 (76.7%)	0.355	13 (92.9%)	10 (90.9%)	> 0.999	11 (78.6%)	12 (85.7%)	> 0.999	18 (90.0%)	23 (76.7%)	0.2847
Malay	1 (2.8%)	0 (0.0%)		0 (0.0%)	0 (0.0%)		1 (7.1%)	0 (0.0%)		0 (0.0%)	0 (0.0%)	
Indian	0 (0.0%)	1 (3.3%)		0 (0.0%)	0 (0.0%)		0 (0.0%)	0 (0.0%)		0 (0.0%)	1 (0.0%)	
Others	4 (11.1%)	6 (20.0%)		1 (7.1%)	1 (9.1%)		2 (14.3%)	2 (14.3%)		2 (10.0%)	6 (20.0%)	
**Comorbidities**												
Diabetes	4 (11.1%)	0 (0.0%)	0.1196	3 (21.4%)	0 (0.0%)	0.2300	0 (0.0%)	0 (0.0%)	> 0.999	1 (5.0%)	0 (0.0%)	0.4118
Hypertension	8 (22.2%)	1 (3.3%)	0.0331	6 (42.9%)	1 (9.1%)	0.0900	0 (0.0%)	2 (14.3%)	0.4815	2 (10.0%)	2 (6.7%)	> 0.999
**Laboratory findings**												
Platelets, x 10^9/L	183.7 (59.9)	212.1 (56.4)	0.0688	–	–	–	–	–	–	–	–	–
White blood cells, x 10^9/L	4.8 (1.9)	4.7 (1.6)	0.8467	–	–	–	–	–	–	–	–	–
Lymphocytes, x 10^9/L	1.2 (0.6)	1.4 (0.6)	0.2256	–	–	–	–	–	–	–	–	–
Neutrophils, x 10^9/L	3.0 (1.5)	2.7 (1.6)	0.4870	–	–	–	–	–	–	–	–	–
Monocytes, x 10^9/L	0.5 (0.3)	0.5 (0.2)	0.3675	–	–	–	–	–	–	–	–	–
**Clinical manifestation**												
Pneumonia	20 (55.6%)	15 (50.0%)	0.6232	10 (71.4%)	6 (54.6%)	0.4341	6 (42.9%)	8 (57.1%)	0.7064	9 (45.0%)	15 (50.0%)	0.779
Hypoxia	6 (16.7%)	1 (3.3%)	0.1158	6 (42.9%)	1 (9.1%)	0.0900	0 (0.0%)	0 (0.0%)	> 0.999	0 (0.0%)	0 (0.0%)	> 0.999
**Clinical severity**												
No pneumonia (mild)	16 (44.4%)	15 (50.0%)		–	–	–	–	–	–	–	–	–
Pneumonia, without hypoxia (moderate)	14 (38.9%)	14 (46.7%)	0.214	–	–	–	–	–	–	–	–	–
Pneumonia, with hypoxia (severe)	6 (16.7%)	1 (3.3%)		–	–	–	–	–	–	–	–	–
**Length of hospitalization (days)**	13.0 (6.1)	13.8 (5.6)	0.571	–	–	–	–	–	–	–	–	–
**Interval between symptom onset and sample collection, median days (IQR)**	–	–	–	9.5 (6.5-12.3)	6.0 (4.0-9.0)	0.2090	18.5 (16.8-27.8)	19.5 (15.0-25.0)	0.3595	–	–	–
**RNA integrity value of acute sample**	–	–	–	7.6 (0.8)	7.7 (0.7)	0.6490	–	–	–	–	–	–

Data represented as Mean (S.D), Median (IQR) or n (%). COVID-19, Coronavirus Disease-19; WT, wildtype; SARS-CoV-2, Severe Acute Respiratory Syndrome Coronavirus 2. *Continuous variables were compared using unpaired t test and categorical variables were compared using Fisher's exact test. Patients in this cohort were classified into three groups based on clinical severity: mild (no pneumonia on chest radiographs [CXR] at baseline and during hospital admission), moderate (pneumonia on CXR without hypoxia), and severe (pneumonia on CXR with hypoxia [desaturation to ≤94%]).

Supplmental Table 2 Demographics of healthy controls.

Variable	Transcriptomic profiling (n=6)	Systemic cytokine profiling (n=23)	S-flow and peptide ELISA (n=22)
**Demographics**			
Mean age, years	39 (11)	51 (17)	45 (13)
Sex			
Male	4 (66.7%)	9 (39.1%)	9 (40.9%)
Female	2 (33.3%)	14 (60.9%)	12 (54.5%)

*Values reported as number (percentage) for categorical variables and mean (SD) for continuous variables.

Supplemental Table 3 Summary statistics of RNA-seq data and mapping results

Sample Name	Total Sequences (millions)	% Assigned reads	Assigned reads (millions)	% Uniquely mapped reads	Uniquely mapped reads (millions)	Average % GC Content
**R10830**	22.2	50.30%	27.5	85.80%	19	48%
**R10831_R**	17	53.70%	22.2	86.10%	14.7	49%
**R10832**	20	49.30%	25.8	86.00%	17.2	49%
**R10833**	19.8	48.90%	26.5	84.70%	16.8	49%
**R10834**	20.1	48.20%	23.8	85.90%	17.3	48%
**R10835_R**	16.7	55.50%	22.7	85.50%	14.3	49%
**R10836**	22.6	40.10%	27	81.60%	18.4	50%
**R10837**	20.1	45.20%	23.7	84.10%	16.9	49%
**R10838**	23.9	51.20%	30.3	86.80%	20.7	48%
**R10839_R**	18.6	47.60%	20.9	85.90%	16	48%
**R10840_R**	20.6	58.20%	28.2	86.90%	17.9	49%
**R10841**	22.5	37.60%	28.1	77.30%	17.4	51%
**R10842**	21.9	37.80%	27.1	76.60%	16.7	51%
**R10843**	20.2	40.10%	24.9	77.30%	15.6	51%
**R10844**	21.8	48.20%	27.8	84.70%	18.5	50%
**R10845**	22.3	49.90%	29	84.70%	18.8	50%
**R10846**	22.2	56.00%	30.8	86.30%	19.2	49%
**R10847**	20.6	43.80%	24.4	82.90%	17.1	49%
**R10848**	20.1	55.20%	26.4	87.20%	17.5	48%
**R10849**	21.7	50.50%	23.8	88.30%	19.2	48%
**R10852_R**	21.9	24.20%	10.3	87.00%	19.1	44%
**R10853_R**	21.3	53.90%	28.6	85.10%	18.1	49%
**R10854_R**	20	50.20%	26.4	84.10%	16.8	47%
**R10855**	20.5	34.10%	23.2	74.10%	15.2	51%
**R10856**	21.5	58.40%	29.4	87.10%	18.7	49%
**R10857**	22.1	48.30%	27.3	84.90%	18.8	50%
**R10858**	17.3	35.50%	21.3	76.70%	13.2	51%
**R10859**	20.5	47.80%	26	84.90%	17.4	49%
**R10860**	19.5	56.90%	27	86.60%	16.9	50%
**R10861**	20.4	46.00%	26.5	83.20%	17	50%
**R10862**	21.3	59.20%	27.9	88.90%	18.9	47%
**R10863_R**	22.8	59.50%	31.2	88.00%	20.1	49%
**R10864_R**	21.6	62.90%	30.9	87.00%	18.8	51%
**R10865**	23.7	44.80%	30.4	81.50%	19.3	51%
**R10866_R**	24.2	59.60%	31.2	88.60%	21.4	49%
**R10867**	21.1	41.60%	21.2	84.00%	17.7	48%
**R10868**	21.1	52.00%	27.7	85.00%	17.9	50%
**R10869**	24.9	35.70%	26.5	76.10%	19	51%
**R10870**	18.8	56.80%	25.3	87.10%	16.3	49%
**R10871_R**	14.4	41.60%	18.1	77.70%	11.2	51%
**R10872_R**	14	48.20%	16.9	84.30%	11.8	49%
**R10873**	21.4	41.80%	24.6	80.10%	17.2	50%
**R10874**	18.4	54.40%	24	85.80%	15.8	48%
**R10875**	17.6	34.90%	17.9	74.60%	13.1	50%
**R10876_2**	17.6	35.80%	19.9	73.50%	12.9	53%
**R10877_2**	21.2	43.30%	25.1	81.30%	17.2	51%
**R10878**	23.6	50.20%	29.1	86.90%	20.5	47%
**R10879**	23.5	51.20%	28.5	86.90%	20.4	47%
**R10880**	24	47.90%	29	85.50%	20.5	48%
**R10881_R**	24.7	52.70%	31	87.10%	21.5	48%
**R10882**	21	43.00%	27.2	77.20%	16.2	47%
**R10883**	24.1	50.00%	31.1	85.20%	20.5	49%
**R10884_R**	19.6	56.50%	27.4	86.10%	16.9	49%
**R10885**	26.6	44.70%	30.5	83.40%	22.2	49%
**R10886_2**	22.9	40.20%	28.3	74.00%	16.9	53%
**R10887**	22.3	42.20%	28	79.00%	17.6	50%

Supplemental Table 4 Flow cytometry antibodies for T cell Intracellular Panel (1 million PBMCs)

No.	Marker	Colour	Volume (µL)	Clone	Cat. No.	Vendor
1	CD66B	BV421	2	G10F5	562940	BD Biosciences
2	CD45RA	SB436	2	H100	62-0458-42	Thermo Fisher
3	CD27	PB	1	O323	302822	Biolegend
4	NKG2C	BV480	2	134591	748168	BD Biosciences
5	CD8	BV605	1	SK1	564116	BD Biosciences
6	CD19	BV605	1	HIB19	740394	BD Biosciences
7	VD2	BV711	1	B6	331412	Biolegend
8	CD107A	BV785	2	H4A3	563869	BD Biosciences
9	NKP46	BB515	2	9-e2	564536	BD Biosciences
10	CD3	SB550	1	SK7	344852	Biolegend
11	CD169	PERCP5.5	5	7-239	346020	Biolegend
12	HLADR	APCR700	2	L243	307626	Biolegend
13	CD4	SN685	1	SK3	344658	Biolegend
14	CD14	APC CY7	1	M0P9	557831	BD Biosciences
15	VD1	APC Vio770	1	REA173	130-120-578	Miltenyi Biotec
16	L/D	ZOMBIE NIR	0.5		423105	Biolegend
17	CXCR5	PE VIO615	1	J252D4	356928	Biolegend
18	CD154	PE CY5	5	TRAP-1	555701	BD Biosciences
19	CD56	PE CY5.5	2	NCAM16.2	35-0567-42	Thermo Fisher
20	CD16	PEAF700	1	3G8	MHCD1624	Thermo Fisher
21	CD45	BUV805	2	H130	612891	BD Biosciences
22	Granzyme B	BV510	2.5	GB11	563388	BD Biosciences
23	IFN-y	BV570	2.5	4S.B3	502534	Biolegend
24	IL-2	BV650	5	MQ1-17H12	564166	BD Biosciences
25	TNF-a	BV750	2.5	MAB11	566359	BD Biosciences
26	IL-6	FITC	2.5	MQ2-13A5	11-7069-82	Thermo Fisher
27	IL-17a	AF647	10	N49-653	560490	BD Biosciences
28	IL-10	PE	2.5	JES-9D7	501404	Biolegend
29	IL-4	PECY7	2.5	8D4-8	560672	BD Biosciences

Supplemental Table 5 List of DEGs between blood samples collected at acute and recovered phases of WT SARS-CoV-2 infected patients, with thresholds of *p*-value < 0.01 and |FC| > 2.

Gene ID	Length (bp)	Gene Name	Gene Type	logFC	logCPM	F	*p*-value	FDR	Remarks
ENSG00000267218.2	939	AC005336.2	processed_pseudogene	-6.298339339	-0.605767348	18.63446825	0.001879262	0.151667494	Over-expressed in SARS-CoV-2 infection
ENSG00000154227.13	5130	CERS3	protein_coding	-5.839249686	-0.785108944	20.13948473	0.000318621	0.080341116	
ENSG00000181109.3	963	OR52P1P	unprocessed_pseudogene	-5.807800445	-0.593578552	12.77214025	0.002150191	0.156703233	
ENSG00000233327.10	6442	USP32P2	transcribed_unprocessed_pseudogene	-5.709319401	-1.739738352	16.21260744	0.002903465	0.177137514	
ENSG00000187244.11	5009	BCAM	protein_coding	-5.692264175	0.828586243	22.71843737	0.00010267	0.065041535	
ENSG00000174358.16	5270	SLC6A19	protein_coding	-5.466825449	3.141339	12.10100015	0.00249448	0.166708468	
ENSG00000254231.3	5462	AC103760.1	lncRNA	-5.196804951	0.00285794	18.10041512	0.000526864	0.0890476	
ENSG00000117425.14	5334	PTCH2	protein_coding	-5.048643609	-0.593168919	12.0602009	0.002384337	0.16215461	
ENSG00000286245.1	3913	AC125437.3	lncRNA	-4.94428407	-1.299840112	13.49408262	0.00172154	0.14926841	
ENSG00000236756.4	3260	DNAJC9-AS1	lncRNA	-4.94049194	-1.356387984	10.75583413	0.004676787	0.218094049	
ENSG00000278991.1	1514	AC090181.3	TEC	-4.932380358	-0.560787812	18.46982394	0.000544109	0.090274686	
ENSG00000112530.11	2612	PACRG	protein_coding	-4.908301271	-1.366799744	13.18603105	0.005347447	0.22866216	
ENSG00000229832.1	496	AL360091.1	lncRNA	-4.740799797	-0.965175268	20.01024699	0.000206857	0.068924679	
ENSG00000228352.2	635	AL354989.1	lncRNA	-4.667213777	-1.128277893	15.05944958	0.000920848	0.120216809	
ENSG00000279981.1	2291	AC018445.4	TEC	-4.650527331	-1.171687949	9.705168899	0.005937816	0.234994602	
ENSG00000140057.9	4984	AK7	protein_coding	-4.620005094	0.073203848	11.62795058	0.004592916	0.215832975	
ENSG00000230450.1	1329	NEK2P4	processed_pseudogene	-4.607017459	-1.021468555	14.75828077	0.001421949	0.138767901	
ENSG00000163827.13	5634	LRRC2	protein_coding	-4.546433832	2.666374499	25.0242428	6.71239E-05	0.065041535	
ENSG00000167880.7	7510	EVPL	protein_coding	-4.532024866	-0.527250904	9.862836991	0.00592107	0.234994602	
ENSG00000174370.10	3784	C11orf45	protein_coding	-4.520935553	-0.352507354	8.838323703	0.00941098	0.281412434	
ENSG00000138623.10	3800	SEMA7A	protein_coding	-4.518382265	0.274585418	10.93402308	0.004135932	0.209254815	
ENSG00000248885.1	1271	AC118465.1	processed_pseudogene	-4.512596193	-2.315319775	11.65261016	0.007547811	0.261971937	
ENSG00000188451.8	1979	SRP72P2	processed_pseudogene	-4.496074788	-0.518930929	9.027015122	0.006721603	0.247341607	
ENSG00000236885.2	1821	AC018731.1	lncRNA	-4.475861218	-2.241439929	11.4535093	0.0079093	0.267357252	
ENSG00000227962.1	282	AL391994.1	processed_pseudogene	-4.373941077	-2.032428876	10.15912348	0.005352836	0.22866216	
ENSG00000229740.3	1716	U91324.1	lncRNA	-4.369283585	-1.843858303	10.56388977	0.004976311	0.22287172	
ENSG00000260194.1	739	AC007496.1	lncRNA	-4.350625169	-0.732864242	12.95493737	0.001895085	0.152361738	
ENSG00000226026.6	4074	AC092802.1	lncRNA	-4.350202178	0.994802839	10.43425397	0.004171417	0.210138528	
ENSG00000212993.5	4964	POU5F1B	protein_coding	-4.34247381	-0.017657446	14.95610229	0.001026718	0.122806049	
ENSG00000186453.13	2281	FAM228A	protein_coding	-4.336166652	-2.178451765	11.75690677	0.004024893	0.207233104	
ENSG00000271414.1	445	AC093107.2	processed_pseudogene	-4.330666419	-1.772661999	10.82613654	0.009196102	0.279647818	
ENSG00000251614.1	1517	HSPA8P19	processed_pseudogene	-4.330027521	-1.477382313	12.31500222	0.002876178	0.177002596	
ENSG00000287164.1	597	AC112254.1	lncRNA	-4.25730887	-2.159888226	14.04453695	0.001914834	0.152361738	
ENSG00000173261.9	1937	PLAC8L1	protein_coding	-4.22377309	-1.891680582	8.981213352	0.008476756	0.27270601	
ENSG00000230470.1	423	AL078645.1	lncRNA	-4.220881133	-2.057471241	10.35899895	0.005689677	0.232183786	
ENSG00000177736.4	547	FXNP2	processed_pseudogene	-4.183625014	-0.584750198	9.593449059	0.005649056	0.231803695	
ENSG00000278709.2	2625	NKILA	lncRNA	-4.178603847	-1.506359612	8.984817135	0.008466102	0.27270601	
ENSG00000204528.3	593	PSORS1C3	lncRNA	-4.159685868	-1.072487653	8.550337624	0.009867796	0.285908666	
ENSG00000227658.1	381	AC005160.1	lncRNA	-4.140350185	-2.72404242	11.04318535	0.008723701	0.274220475	
ENSG00000166444.19	14262	ST5	protein_coding	-4.131981941	0.017787778	8.758437217	0.008011152	0.268851943	
ENSG00000234134.1	300	AL158835.2	lncRNA	-4.129635809	-2.321122428	12.94328568	0.002384627	0.16215461	
ENSG00000270911.1	1251	AC114878.2	processed_pseudogene	-4.122557571	-1.358523858	9.264437065	0.007288839	0.257611314	
ENSG00000278022.1	459	AC118658.1	lncRNA	-4.121603477	-2.186207332	9.972082699	0.006046032	0.237181767	
ENSG00000232557.3	210	AL590609.3	lncRNA	-4.116021821	-2.017506385	12.91099085	0.002059251	0.153627079	
ENSG00000279020.1	2534	C18orf15	TEC	-4.082507058	0.299318535	18.24009956	0.000368851	0.081592857	
ENSG00000224885.1	718	EIPR1-IT1	lncRNA	-4.081421946	-2.064733008	11.18631839	0.004076451	0.208000207	
ENSG00000145949.10	6059	MYLK4	protein_coding	-4.04634939	0.21153915	11.22110687	0.003168747	0.182931193	
ENSG00000233668.1	645	AL353662.2	processed_pseudogene	-4.045531103	-0.411623488	13.2893205	0.001705587	0.14926841	
ENSG00000204385.12	3372	SLC44A4	protein_coding	-3.996846761	-1.776464398	10.04900829	0.006749396	0.247341607	
ENSG00000103742.12	7619	IGDCC4	protein_coding	-3.946715528	-1.861033852	9.760799202	0.007978731	0.268642794	
ENSG00000224019.1	477	RPL21P32	processed_pseudogene	-3.933221262	-1.685197722	8.286931055	0.009943297	0.286671761	
ENSG00000218676.1	1522	BRD7P4	processed_pseudogene	-3.902886131	-0.444562009	10.47531709	0.004546804	0.215773794	
ENSG00000259116.2	1234	AL049869.3	lncRNA	-3.845070134	-2.152639678	8.765246706	0.008706603	0.274052374	
ENSG00000257624.1	247	AC004024.1	processed_pseudogene	-3.827085908	-1.950468647	8.264147781	0.009656622	0.283700719	
ENSG00000259225.7	2687	LINC02345	lncRNA	-3.767859999	-0.947553797	8.944547555	0.007474934	0.261779838	
ENSG00000176349.11	4764	AC104129.1	lncRNA	-3.730841027	-0.32119919	12.4340248	0.002567769	0.167668059	
ENSG00000278376.1	1884	AP004609.3	lncRNA	-3.72176106	-2.199018857	11.25883799	0.003299234	0.186043106	
ENSG00000229014.3	348	RPL30P13	processed_pseudogene	-3.694885866	-2.326316827	9.411622393	0.008274988	0.271700571	
ENSG00000163106.10	2111	HPGDS	protein_coding	-3.667205574	-2.310084913	8.886918236	0.009255509	0.280387279	
ENSG00000235763.1	220	SNRPGP5	processed_pseudogene	-3.519503016	-2.532386218	9.537603571	0.006300189	0.241675201	
ENSG00000229106.1	1267	BTBD6P1	processed_pseudogene	-3.496584909	-0.196227757	9.366417678	0.006141962	0.238361261	
ENSG00000087085.15	4074	ACHE	protein_coding	-3.432343356	2.614455962	12.1030155	0.002225719	0.157713304	
ENSG00000238835.1	134	SCARNA18	snoRNA	-3.398741132	-2.26259297	10.6413814	0.004073407	0.208000207	
ENSG00000185662.9	1647	SMIM23	protein_coding	-3.377274707	-2.460074912	10.45537345	0.005946187	0.234994602	
ENSG00000286151.1	1095	AL732406.1	unprocessed_pseudogene	-3.322263271	-0.259124516	8.565341701	0.009359818	0.28078551	
ENSG00000118777.12	5602	ABCG2	protein_coding	-3.217430555	3.064744619	18.6814648	0.000297115	0.080341116	
ENSG00000145423.5	1996	SFRP2	protein_coding	-3.168906089	2.674698249	9.790219771	0.005047338	0.223309078	
ENSG00000207092.1	102	RF00019	misc_RNA	-3.158678405	-0.413196891	10.8723611	0.003411128	0.190337677	
ENSG00000011028.14	6591	AC080038.1	protein_coding	-3.137758094	4.146503896	12.669939	0.001839573	0.15097794	
ENSG00000273348.1	407	AC027449.1	lncRNA	-3.13055769	-2.856214549	9.523336448	0.007468406	0.261779838	
ENSG00000105366.15	3171	SIGLEC8	protein_coding	-2.972497337	3.404602724	10.36933672	0.004084381	0.208000207	
ENSG00000204815.10	3370	TTC25	protein_coding	-2.969013813	3.35497538	32.0173117	1.26094E-05	0.042014513	
ENSG00000055118.14	5877	KCNH2	protein_coding	-2.939636453	1.859940173	9.146917849	0.006422036	0.243557005	
ENSG00000186642.16	7182	PDE2A	protein_coding	-2.916221878	1.530015203	8.93769634	0.006954823	0.250446853	
ENSG00000180537.13	4098	RNF182	protein_coding	-2.730172327	4.253433139	13.93971655	0.001216177	0.130531104	
ENSG00000243064.8	10722	ABCC13	transcribed_unprocessed_pseudogene	-2.667797978	4.232004003	14.62558359	0.000979401	0.122419737	
ENSG00000170180.22	4251	GYPA	protein_coding	-2.667294154	3.456862672	14.12455065	0.001146707	0.129462221	
ENSG00000265093.2	296	RN7SL246P	misc_RNA	-2.644401305	0.022663582	8.932153646	0.006969588	0.250446853	
ENSG00000166947.14	3039	EPB42	protein_coding	-2.570706168	8.314974887	17.73601177	0.000387547	0.081592857	
ENSG00000143416.21	3476	SELENBP1	protein_coding	-2.498464036	8.949814154	19.84727694	0.000216102	0.070145587	
ENSG00000238243.3	945	OR2W3	protein_coding	-2.440731657	5.428717454	23.43724317	8.59142E-05	0.065041535	
ENSG00000182685.7	2083	BRICD5	protein_coding	-2.415894722	1.856331588	10.07694546	0.004542417	0.215773794	
ENSG00000103534.17	6206	TMC5	protein_coding	-2.37950741	2.150417652	11.04165287	0.003212907	0.183646793	
ENSG00000167768.4	2488	KRT1	protein_coding	-2.366400899	6.177462498	10.26929451	0.004235085	0.210788263	
ENSG00000204010.3	1972	IFIT1B	protein_coding	-2.353539721	7.467607657	14.23106462	0.001108665	0.1267574	
ENSG00000133742.13	4068	CA1	protein_coding	-2.336607322	10.42501399	18.6672813	0.000298287	0.080341116	
ENSG00000008441.16	6302	NFIX	protein_coding	-2.322864895	4.677325442	14.54781148	0.001003508	0.122419737	
ENSG00000238105.7	10957	GOLGA2P5	transcribed_unprocessed_pseudogene	-2.283829817	2.910242507	25.4837271	5.25797E-05	0.063559168	
ENSG00000146215.13	2453	CRIP3	protein_coding	-2.265987484	0.692604123	8.043686868	0.009855199	0.285898826	
ENSG00000079308.19	16281	TNS1	protein_coding	-2.261779883	7.606195821	20.54219655	0.000179576	0.065041535	
ENSG00000155962.13	2840	CLIC2	protein_coding	-2.239371094	4.148332303	13.88460257	0.001237778	0.130531104	
ENSG00000105610.5	1611	KLF1	protein_coding	-2.210579635	5.585915267	8.405291447	0.008545535	0.273703784	
ENSG00000213934.8	1659	HBG1	protein_coding	-2.210120048	3.083672764	17.06489221	0.000470021	0.08432891	
ENSG00000197993.9	3660	KEL	protein_coding	-2.209637467	4.511246465	13.76106571	0.001287749	0.130531104	
ENSG00000100012.11	3663	SEC14L3	protein_coding	-2.207840795	3.031294492	10.07941391	0.00453832	0.215773794	
ENSG00000206172.8	835	HBA1	protein_coding	-2.206430613	10.78512269	19.44321775	0.000241042	0.073013806	
ENSG00000213139.8	2080	CRYGS	protein_coding	-2.202909118	1.169732624	17.29062918	0.000440305	0.083493325	
ENSG00000130300.9	2295	PLVAP	protein_coding	-2.140490696	4.712349303	10.35417583	0.004106833	0.208234296	
ENSG00000080823.23	10986	MOK	protein_coding	-2.133025824	0.803228219	9.466146338	0.005694097	0.232183786	
ENSG00000188536.13	827	HBA2	protein_coding	-2.120397515	13.79958074	17.65075466	0.000397083	0.081592857	
ENSG00000162366.8	1430	PDZK1IP1	protein_coding	-2.103684194	6.284177916	26.6285298	4.03465E-05	0.063559168	
ENSG00000004939.15	5125	SLC4A1	protein_coding	-2.101108195	7.574118859	11.71760545	0.002539023	0.167668059	
ENSG00000250361.8	1872	GYPB	protein_coding	-2.091208191	2.83278523	9.962961379	0.004736127	0.218311104	
ENSG00000213088.12	2106	ACKR1	protein_coding	-2.08304391	3.429323016	10.845931	0.003443276	0.190917048	
ENSG00000278897.1	2271	AC020951.1	TEC	-2.075895639	2.189287147	9.094214639	0.006551849	0.244504525	
ENSG00000204613.11	3825	TRIM10	protein_coding	-2.039390933	4.066874359	10.72103591	0.003599787	0.197556298	
ENSG00000184792.16	6632	OSBP2	protein_coding	-2.004069196	7.934397545	26.72821616	3.94393E-05	0.063559168	
ENSG00000197256.10	7638	KANK2	protein_coding	-1.983573384	3.808067336	8.323213376	0.008824914	0.274577975	
ENSG00000070182.21	14796	SPTB	protein_coding	-1.958331227	6.943093208	22.54241817	0.0001073	0.065041535	
ENSG00000213205.3	1281	STRADBP1	processed_pseudogene	-1.928319257	1.717828647	9.966684919	0.004729656	0.218311104	
ENSG00000082146.13	3177	STRADB	protein_coding	-1.928133542	10.19628785	12.886267	0.001712221	0.14926841	
ENSG00000145335.15	4014	SNCA	protein_coding	-1.919301047	9.641378126	20.51852127	0.000180702	0.065041535	
ENSG00000013306.16	3526	SLC25A39	protein_coding	-1.91161816	13.36704743	16.07817246	0.000628487	0.095187285	
ENSG00000168477.19	15909	TNXB	protein_coding	-1.911518657	2.258874345	11.36612871	0.002867563	0.177002596	
ENSG00000244734.4	1304	HBB	protein_coding	-1.909114923	13.99645604	15.45150523	0.000759223	0.10732189	
ENSG00000226380.9	6619	AC016831.1	lncRNA	-1.891200421	2.929223018	26.44282445	4.20982E-05	0.063559168	

ENSG00000141469.18	7182	SLC14A1	protein_coding	-1.874849754	7.083279227	12.62994067	0.001864245	0.15097794
ENSG00000167992.13	4766	VWCE	protein_coding	-1.851026914	4.311493751	12.17139267	0.00217472	0.156703233
ENSG00000100225.18	8521	FBXO7	protein_coding	-1.848290768	12.20228857	20.52248416	0.000180513	0.065041535
ENSG00000149260.18	6137	CAPN5	protein_coding	-1.833801546	3.771092869	15.03229003	0.000863276	0.115718712
ENSG00000164849.10	3190	GPR146	protein_coding	-1.807498303	3.733834889	12.78639175	0.001769768	0.150649855
ENSG00000171552.13	3314	BCL2L1	protein_coding	-1.791357241	9.092592377	21.4133575	0.00014304	0.065041535
ENSG00000107262.22	7073	BAG1	protein_coding	-1.785270946	8.02000383	16.50631547	0.000553476	0.090274686
ENSG00000066926.11	8772	FECH	protein_coding	-1.770066832	8.326228783	17.3887184	0.000428042	0.083197062
ENSG00000145721.12	3996	LIX1	protein_coding	-1.76713857	1.686994271	9.015994582	0.006749905	0.247341607
ENSG00000168785.8	7412	TSPAN5	protein_coding	-1.765031954	7.226393668	13.81539186	0.001265506	0.130531104
ENSG00000130821.16	5666	SLC6A8	protein_coding	-1.739048237	6.086770812	16.50830729	0.000553151	0.090274686
ENSG00000124098.10	3518	FAM210B	protein_coding	-1.733225273	9.197373473	15.70768842	0.000702475	0.103047287
ENSG00000100325.15	5415	ASCC2	protein_coding	-1.725702036	10.22011417	18.03885675	0.000355656	0.081326674
ENSG00000165406.16	7516	MARCH8	protein_coding	-1.719369461	9.72298429	19.3361962	0.000248166	0.074207937
ENSG00000198876.13	4315	DCAF12	protein_coding	-1.715041409	10.99733602	14.53436386	0.001007743	0.122419737
ENSG00000197992.7	2857	CLEC9A	protein_coding	-1.696685937	1.426737693	9.531423924	0.005556782	0.231803695
ENSG00000136929.13	2192	HEMGN	protein_coding	-1.695121408	7.154726789	13.00384858	0.001647099	0.147171585
ENSG00000136732.16	3609	GYPC	protein_coding	-1.694088026	10.61571168	14.3469068	0.001068862	0.124797349
ENSG00000166046.11	6616	TCP11L2	protein_coding	-1.660178096	7.408561265	16.43303439	0.000565584	0.09160894
ENSG00000119950.21	5907	MXI1	protein_coding	-1.659207071	8.584891358	21.50323643	0.000139762	0.065041535
ENSG00000198892.6	1746	SHISA4	protein_coding	-1.649385717	3.568315197	10.84606396	0.003443113	0.190917048
ENSG00000060138.13	8621	YBX3	protein_coding	-1.64542595	8.912071455	19.73901919	0.000222494	0.070145587
ENSG00000104765.16	4149	BNIP3L	protein_coding	-1.641878437	11.13898142	18.25760932	0.000334415	0.080811282
ENSG00000029534.20	10797	ANK1	protein_coding	-1.623817441	7.39091899	13.70094907	0.001312867	0.131430185
ENSG00000137267.6	1748	TUBB2A	protein_coding	-1.623559507	5.354246216	11.21797463	0.00301988	0.178770775
ENSG00000103342.13	8054	GSPT1	protein_coding	-1.623483193	8.66109277	25.15696135	5.67799E-05	0.063559168
ENSG00000063854.13	5839	HAGH	protein_coding	-1.613656152	8.126906026	21.44443641	0.000141897	0.065041535
ENSG00000223609.11	1152	HBD	protein_coding	-1.607206114	8.673839777	13.42585277	0.001434898	0.139448215
ENSG00000076770.14	12199	MBNL3	protein_coding	-1.595879233	8.665831234	16.78585088	0.000509839	0.088431676
ENSG00000260592.1	633	AC130456.3	lncRNA	-1.594526296	2.152723778	8.620439989	0.007858665	0.266788783
ENSG00000065491.8	3462	TBC1D22B	protein_coding	-1.584306013	5.155991334	12.94314557	0.001680371	0.148458274
ENSG00000132819.17	2697	RBM38	protein_coding	-1.580579819	7.852540522	18.15170297	0.000344519	0.081167395
ENSG00000118689.15	7670	FOXO3	protein_coding	-1.566237353	9.461040672	20.73425288	0.000170718	0.065041535
ENSG00000167671.12	5247	UBXN6	protein_coding	-1.560375799	8.208270923	17.62785407	0.000399688	0.081592857
ENSG00000136842.14	3676	TMOD1	protein_coding	-1.544435458	6.372417046	9.041153807	0.006685486	0.247163348
ENSG00000147454.14	7996	SLC25A37	protein_coding	-1.525242894	12.71180227	17.61473672	0.000401189	0.081592857
ENSG00000186654.21	5241	PRR5	protein_coding	-1.523801455	4.892516089	15.35740079	0.000781319	0.108473153
ENSG00000250616.2	1650	AC012645.1	lncRNA	-1.520468717	4.035207452	11.29020176	0.002944536	0.177463481
ENSG00000169877.10	526	AHSP	protein_coding	-1.499926634	4.832588149	10.26704761	0.004238539	0.210788263
ENSG00000153574.9	1813	RPIA	protein_coding	-1.495983928	5.327492462	14.76396213	0.000938066	0.120216809
ENSG00000170315.13	1621	UBB	protein_coding	-1.49443889	10.42381022	17.79072007	0.000381561	0.081592857
ENSG00000182389.19	36268	CACNB4	protein_coding	-1.492433254	3.44640314	21.64858132	0.000134634	0.065041535
ENSG00000198336.9	1636	MYL4	protein_coding	-1.478102535	5.46792543	11.51704048	0.002721079	0.172933077
ENSG00000110693.18	19640	SOX6	protein_coding	-1.462383733	4.415755323	9.60357725	0.005409251	0.229770887
ENSG00000057757.10	2173	PITHD1	protein_coding	-1.457003553	7.255932887	12.65900327	0.001846282	0.15097794
ENSG00000154114.12	7020	TBCEL	protein_coding	-1.452110255	3.834299574	11.59794667	0.002645961	0.170012088
ENSG00000164398.14	16996	ACSL6	protein_coding	-1.449462704	3.368706446	14.56689338	0.000997533	0.122419737
ENSG00000125818.18	10361	PSMF1	protein_coding	-1.444721528	9.554569581	18.85632485	0.00028308	0.079548781
ENSG00000162722.9	5170	TRIM58	protein_coding	-1.439074741	9.251175669	15.47586885	0.000753615	0.107178706
ENSG00000141084.11	6456	RANBP10	protein_coding	-1.439023214	6.968403287	14.04455376	0.001176222	0.129462221
ENSG00000204673.10	5737	AKT1S1	protein_coding	-1.438061392	2.440078338	8.752156428	0.007468494	0.261779838
ENSG00000159023.21	19993	EPB41	protein_coding	-1.438032199	8.359746832	18.09678709	0.00034989	0.081326674
ENSG00000158856.18	4473	DMTN	protein_coding	-1.4324535	9.133269404	12.76591996	0.001781825	0.150810809
ENSG00000133606.11	5843	MKRN1	protein_coding	-1.426578586	9.928071453	20.54615939	0.000179388	0.065041535
ENSG00000137216.19	4425	TMEM63B	protein_coding	-1.42331919	4.695624432	9.430008813	0.005771727	0.233309816
ENSG00000211785.1	340	TRAV12-1	TR_V_gene	-1.421378439	2.769727632	8.077338414	0.009724363	0.284224372
ENSG00000263563.6	1815	UBBP4	transcribed_unprocessed_pseudogene	-1.419538801	3.803148299	14.69262934	0.000959129	0.122244368
ENSG00000143776.18	14055	CDC42BPA	protein_coding	-1.417317891	4.178104018	18.91640776	0.000278426	0.079195322
ENSG00000112146.16	8164	FBXO9	protein_coding	-1.41406279	8.126530145	17.67010615	0.000394896	0.081592857
ENSG00000136819.15	5099	C9orf78	protein_coding	-1.407684161	9.947176954	20.39730586	0.000186592	0.065734645
ENSG00000123240.17	5138	OPTN	protein_coding	-1.386668257	8.973720677	16.36355389	0.000577334	0.091657831
ENSG00000246548.3	2924	LINC02288	lncRNA	-1.381099384	3.732829057	10.58791266	0.003775311	0.201892848
ENSG00000180089.5	2626	TMEM86B	protein_coding	-1.373616147	3.797732505	11.67427407	0.002577187	0.167668059
ENSG00000099804.9	1894	CDC34	protein_coding	-1.367593545	4.676219128	8.459916387	0.008365046	0.272163583
ENSG00000133816.15	20191	MICAL2	protein_coding	-1.363991132	7.454238845	17.56939575	0.000406424	0.081592857
ENSG00000166169.17	5420	POLL	protein_coding	-1.358622721	6.307340703	14.49476308	0.001020327	0.122670606
ENSG00000175931.13	6413	UBE2O	protein_coding	-1.348315948	6.843494066	11.48702223	0.002749545	0.173795108
ENSG00000136908.17	3133	DPM2	protein_coding	-1.345617763	7.821105353	16.26384904	0.000594666	0.092733724
ENSG00000155749.12	2635	FLACC1	protein_coding	-1.343185087	2.831418887	9.296749334	0.006068285	0.237477652
ENSG00000183508.5	5654	TENT5C	protein_coding	-1.33021487	9.666048046	14.67171693	0.000965402	0.122375173
ENSG00000253982.2	2264	AC100810.1	lncRNA	-1.32928591	4.133659605	15.15895851	0.000830271	0.113246973
ENSG00000005187.12	7510	ACSM3	protein_coding	-1.328958344	3.220021315	13.16548159	0.001561991	0.14488155
ENSG00000182108.11	6692	DEXI	protein_coding	-1.322794181	2.568882919	13.96035331	0.001208195	0.130531104
ENSG00000187097.12	10379	ENTPD5	protein_coding	-1.320972104	4.133149559	8.27543798	0.00899219	0.277604571
ENSG00000196914.9	24906	ARHGEF12	protein_coding	-1.318589868	7.07997929	12.1587955	0.002184018	0.156738541
ENSG00000245552.7	7431	AP000787.1	lncRNA	-1.314879863	5.459724916	12.21880343	0.002140115	0.156703233
ENSG00000090013.10	1689	BLVRB	protein_coding	-1.301607455	8.319288723	17.18790973	0.000453563	0.084035778
ENSG00000196407.12	1161	THEM5	protein_coding	-1.294838315	2.381073728	12.92669021	0.001689518	0.148703089
ENSG00000101782.15	4856	RIOK3	protein_coding	-1.27995542	9.418724289	14.0656755	0.001168349	0.129462221
ENSG00000172270.19	3797	BSG	protein_coding	-1.276508478	10.23189528	15.52123474	0.000743293	0.106359316
ENSG00000149212.11	10573	SESN3	protein_coding	-1.270009689	7.224996394	25.14276977	5.69704E-05	0.063559168
ENSG00000106714.17	9521	CNTNAP3	protein_coding	-1.263113146	4.176736716	8.178864008	0.009341182	0.28078551
ENSG00000173868.11	2375	PHOSPHO1	protein_coding	-1.254974035	7.233026973	12.00074138	0.002304479	0.159849169
ENSG00000159346.13	3113	ADIPOR1	protein_coding	-1.249321262	11.90067472	9.837213361	0.004960493	0.22287172
ENSG00000007968.7	5462	E2F2	protein_coding	-1.238951544	5.940457414	11.30287335	0.002931533	0.177137514
ENSG00000181704.12	8608	YIPF6	protein_coding	-1.237698717	6.981263478	22.80866541	0.000100382	0.065041535
ENSG00000172331.12	2539	BPGM	protein_coding	-1.235595439	8.452192199	9.161262681	0.006387198	0.242630297
ENSG00000100104.13	4319	SRRD	protein_coding	-1.227685808	4.290272723	11.94539273	0.002348381	0.161350833
ENSG00000240445.4	6188	FOXO3B	protein_coding	-1.212467356	3.182789082	10.75777895	0.003552942	0.195907375
ENSG00000147324.11	6868	MFHAS1	protein_coding	-1.210012105	4.46532862	16.23384666	0.000599992	0.092733724
ENSG00000104973.18	7687	MED25	protein_coding	-1.198416816	5.598231858	10.29387842	0.00419749	0.21024027
ENSG00000088992.18	1926	TESC	protein_coding	-1.196865877	6.211584691	8.870856127	0.007135216	0.253691739
ENSG00000115993.13	7516	TRAK2	protein_coding	-1.19249501	7.271138666	11.53759907	0.00270177	0.172175118
ENSG00000203709.12	16745	MIR29B2CHG	lncRNA	-1.188382169	5.806564954	18.29653546	0.000330784	0.080811282
ENSG00000135924.15	5886	DNAJB2	protein_coding	-1.180068941	2.94245505	15.27885443	0.000800305	0.110451517
ENSG00000278330.1	3125	AC018529.2	lncRNA	-1.171027812	3.9555758	9.119622358	0.006488908	0.244108531
ENSG00000073792.15	4735	IGF2BP2	protein_coding	-1.16983782	5.11406031	12.37900419	0.002027603	0.152594033
ENSG00000105701.16	2819	FKBP8	protein_coding	-1.14545989	8.434120615	11.36609826	0.002867594	0.177002596
ENSG00000129472.15	3597	RAB2B	protein_coding	-1.142069535	5.931192493	17.48565678	0.00041629	0.081592857
ENSG00000227028.6	8475	SLC8A1-AS1	lncRNA	-1.142049987	3.721865468	12.64947509	0.00185215	0.15097794
ENSG00000022840.16	5900	RNF10	protein_coding	-1.127082513	10.40536362	13.04992667	0.001622327	0.146488379
ENSG00000164068.16	7419	RNF123	protein_coding	-1.110935841	6.209563827	13.46559045	0.001416524	0.138767901
ENSG00000205930.10	12896	C21orf62-AS1	lncRNA	-1.103568472	2.503615647	8.369175208	0.008667241	0.273839572
ENSG00000160445.11	4370	ZER1	protein_coding	-1.094694	7.661215292	12.57121662	0.001901131	0.152361738
ENSG00000223705.9	4822	NSUN5P1	transcribed_unprocessed_pseudogene	-1.083600979	4.159706351	23.80864716	7.84517E-05	0.065041535
ENSG00000198959.12	7948	TGM2	protein_coding	-1.08091174	5.444909252	8.295848907	0.008920299	0.276671604
ENSG00000179262.10	2547	RAD23A	protein_coding	-1.080659119	7.777043086	10.74119333	0.003574004	0.196603915
ENSG00000196998.19	4691	WDR45	protein_coding	-1.074258947	7.010978349	18.20342969	0.000339543	0.080811282
ENSG00000230426.4	12088	ERVMER61-1	lncRNA	-1.073583731	4.312702918	10.15299063	0.004418061	0.214235683
ENSG00000182183.15	2684	SHISAL2A	protein_coding	-1.064697546	2.234902084	8.549610617	0.008077784	0.269922964
ENSG00000205639.11	4522	MFSD2B	protein_coding	-1.063946366	4.950185276	10.08061111	0.004536335	0.215773794
ENSG00000123933.17	6873	MXD4	protein_coding	-1.063362866	4.580149556	13.79435455	0.001274069	0.130531104
ENSG00000065978.19	3395	YBX1	protein_coding	-1.062384005	8.22336376	15.73628775	0.000696435	0.102807861
ENSG00000228794.9	18974	LINC01128	lncRNA	-1.061903737	4.351173374	8.034242091	0.00989227	0.286133791
ENSG00000167815.12	2034	PRDX2	protein_coding	-1.061222931	6.956572349	11.94081479	0.002352053	0.161350833
ENSG00000198932.13	6789	GPRASP1	protein_coding	-1.05750236	3.409911094	8.643367851	0.007789154	0.265605594
ENSG00000103148.15	6609	NPRL3	protein_coding	-1.054499482	7.722658788	11.41207507	0.002822064	0.176796824
ENSG00000034152.18	3783	MAP2K3	protein_coding	-1.054387975	9.801134339	13.51007632	0.00139626	0.137993084
ENSG00000239665.8	15012	AL157392.3	lncRNA	-1.049371359	3.593497653	13.30943286	0.00149026	0.143631501

ENSG00000247982.6	3669	LINC00926	lncRNA	-1.04891495	5.283451625	13.07676006	0.001608091	0.146488379	
ENSG00000165775.18	10094	FUNDC2	protein_coding	-1.047776402	5.687255921	19.53730805	0.000234964	0.072109261	
ENSG00000137193.14	3030	PIM1	protein_coding	-1.038018536	9.234960469	8.8967531	0.007064715	0.252152279	
ENSG00000136840.19	2633	ST6GALNAC4	protein_coding	-1.035578911	7.177228049	12.11700966	0.002215175	0.157713304	
ENSG00000141098.13	8592	GFOD2	protein_coding	-1.035055949	5.966901937	16.59485598	0.000539226	0.090274686	
ENSG00000158828.8	4833	PINK1	protein_coding	-1.034973735	4.203072472	11.06705544	0.003184276	0.182931193	
ENSG00000185986.12	3075	SDHAP3	transcribed_unprocessed_pseudogene	-1.032058619	2.966541901	12.74862484	0.001792081	0.150810809	
ENSG00000253320.7	7435	AZIN1-AS1	lncRNA	-1.027382358	2.113233849	9.228032452	0.006227772	0.240490991	
ENSG00000205885.7	6094	C1RL-AS1	lncRNA	-1.025161309	2.696213649	12.51767959	0.00193546	0.152472606	
ENSG00000177688.6	1066	SUMO4	protein_coding	-1.016712281	2.585759173	9.479594233	0.005665504	0.231828468	
ENSG00000180667.10	6521	YOD1	protein_coding	-1.008350861	6.992407577	8.18901753	0.009303791	0.28078551	
ENSG00000157734.14	5767	SNX22	protein_coding	-1.00768686	3.14607721	10.07020705	0.004553622	0.215773794	
ENSG00000132704.16	5058	FCRL2	protein_coding	-1.00689124	4.238477874	9.847483507	0.004941734	0.22271498	
ENSG00000154122.14	12695	ANKH	protein_coding	-1.004818955	5.959574815	20.96412321	0.000160741	0.065041535	
ENSG00000166171.13	1691	DPCD	protein_coding	-1.001765681	3.754308358	8.495178586	0.008250782	0.271700571	
ENSG00000107882.11	6865	SUFU	protein_coding	1.000465221	3.579427728	12.96528382	0.001668152	0.147939086	Under-expressed in SARS-CoV-2 infection
ENSG00000100985.7	2336	MMP9	protein_coding	1.002197814	6.814514315	9.922423406	0.004807206	0.219849572	
ENSG00000141664.9	10235	ZCCHC2	protein_coding	1.005371764	5.51823007	16.83435807	0.000502659	0.088431676	
ENSG00000145685.14	7977	LHFPL2	protein_coding	1.015985539	3.951588063	17.07573992	0.000468543	0.08432891	
ENSG00000143995.20	10942	MEIS1	protein_coding	1.020417377	4.916598537	13.64528527	0.001336605	0.133226433	
ENSG00000221963.6	10065	APOL6	protein_coding	1.021023751	7.36536504	14.4262124	0.001042521	0.124059969	
ENSG00000117226.12	4284	GBP3	protein_coding	1.023750823	6.518889411	8.134860986	0.009505162	0.283359974	
ENSG00000138798.12	7616	EGF	protein_coding	1.023819173	4.487129796	8.516088434	0.008183844	0.270751741	
ENSG00000168389.17	4007	MFSD2A	protein_coding	1.031635857	3.238704199	8.173203114	0.0093621	0.28078551	
ENSG00000131979.19	2925	GCH1	protein_coding	1.041324519	4.621368964	23.4832545	8.49488E-05	0.065041535	
ENSG00000130303.13	1101	BST2	protein_coding	1.044311403	7.43485147	10.18801365	0.004362057	0.213947052	
ENSG00000105501.12	2360	SIGLEC5	protein_coding	1.047558315	3.404318734	12.75583712	0.001787796	0.150810809	
ENSG00000277075.2	509	HIST1H2AE	protein_coding	1.060861561	2.00036114	8.522323337	0.008164001	0.270478924	
ENSG00000166527.8	2050	CLEC4D	protein_coding	1.081006601	4.969783766	13.56466972	0.001371827	0.136155328	
ENSG00000111911.7	3325	HINT3	protein_coding	1.081827025	3.691504823	11.06840955	0.003182758	0.182931193	
ENSG00000180316.12	3034	PNPLA1	protein_coding	1.097000796	3.152050555	11.22120724	0.003016463	0.178770775	
ENSG00000143891.17	2616	GALM	protein_coding	1.097489059	6.226625991	11.58288571	0.002659769	0.170429817	
ENSG00000132465.11	1651	JCHAIN	protein_coding	1.10017711	7.879395657	8.048461133	0.009836518	0.285712268	
ENSG00000204103.4	3389	MAFB	protein_coding	1.110844988	5.116882024	10.11996984	0.004471591	0.214996632	
ENSG00000132357.14	4275	CARD6	protein_coding	1.116417778	5.088675908	13.81550194	0.001265461	0.130531104	
ENSG00000180596.7	834	HIST1H2BC	protein_coding	1.117411672	5.917645492	21.05596348	0.000156935	0.065041535	
ENSG00000132274.16	5944	TRIM22	protein_coding	1.126965804	8.971059932	14.32734946	0.00107547	0.124797349	
ENSG00000132256.18	4804	TRIM5	protein_coding	1.130481982	5.990893963	13.74893426	0.001292775	0.130531104	
ENSG00000162714.12	10531	ZNF496	protein_coding	1.133822927	4.129755493	12.37822773	0.002028132	0.152594033	
ENSG00000186818.12	6328	LILRB4	protein_coding	1.138091717	5.362528666	12.73045221	0.001802928	0.150810809	
ENSG00000139832.5	1507	RAB20	protein_coding	1.142861092	4.636238436	8.243549943	0.009105798	0.279390877	
ENSG00000138119.17	8490	MYOF	protein_coding	1.146785428	3.975905576	16.52516385	0.000550408	0.090274686	
ENSG00000138035.15	5291	PNPT1	protein_coding	1.162915859	5.409504975	10.13324138	0.004449991	0.214458329	
ENSG00000128383.13	2359	APOBEC3A	protein_coding	1.164026526	8.226427834	18.49165404	0.000313222	0.080341116	
ENSG00000120217.14	4108	CD274	protein_coding	1.169614944	4.947930658	11.03232156	0.003223495	0.183646793	
ENSG00000123610.5	1577	TNFAIP6	protein_coding	1.184359369	4.415665698	14.25462807	0.001100439	0.126436603	
ENSG00000163823.4	2646	CCR1	protein_coding	1.185658741	7.891928335	10.06278005	0.004566007	0.215773794	
ENSG00000185885.16	936	IFITM1	protein_coding	1.185970734	6.696340986	14.27166613	0.001094532	0.126380517	
ENSG00000068079.7	1973	IFI35	protein_coding	1.194710426	7.314035101	14.33229467	0.001073795	0.124797349	
ENSG00000158373.8	871	HIST1H2BD	protein_coding	1.198803485	4.263891595	24.52658107	6.59618E-05	0.065041535	
ENSG00000162614.18	4436	NEXN	protein_coding	1.20464795	4.79549449	26.08270774	4.57377E-05	0.063559168	
ENSG00000116663.11	1735	FBXO6	protein_coding	1.20946605	6.537482247	12.72868318	0.001803988	0.150810809	
ENSG00000115159.16	7165	GPD2	protein_coding	1.209712831	3.898480005	9.497414831	0.005627859	0.231803695	
ENSG00000185880.13	9265	TRIM69	protein_coding	1.218290852	6.518136212	21.61433007	0.000135824	0.065041535	
ENSG00000184678.10	2194	HIST2H2BE	protein_coding	1.223327134	8.370981346	21.34296184	0.000145666	0.065041535	
ENSG00000055332.18	10753	EIF2AK2	protein_coding	1.275857076	7.568463714	17.07728117	0.000468334	0.08432891	
ENSG00000223914.3	5992	LINC02471	lncRNA	1.277839402	1.841891376	8.907473816	0.007035756	0.251690141	
ENSG00000125148.7	914	MT2A	protein_coding	1.282294083	3.822132361	8.624505377	0.00784629	0.26677386	
ENSG00000117228.10	4862	GBP1	protein_coding	1.28953245	8.768459005	13.8642601	0.001245858	0.130531104	
ENSG00000132669.13	8273	RIN2	protein_coding	1.290343954	5.767233111	18.45554739	0.000316393	0.080341116	
ENSG00000152778.9	4029	IFIT5	protein_coding	1.293356935	6.489250889	12.08098826	0.002242428	0.157713304	
ENSG00000185909.15	2171	KLHDC8B	protein_coding	1.298057829	5.076949053	24.92733723	5.99512E-05	0.063559168	
ENSG00000177409.12	7150	SAMD9L	protein_coding	1.303833594	8.08090131	20.69199407	0.000172625	0.065041535	
ENSG00000124256.15	5405	ZBP1	protein_coding	1.30529875	6.6720384	14.10909031	0.001152347	0.129462221	
ENSG00000185507.21	2612	IRF7	protein_coding	1.333838411	8.36677893	8.613354062	0.007880287	0.266788783	
ENSG00000172123.12	3560	SLFN12	protein_coding	1.343893086	3.496047515	22.32664843	0.00011329	0.065041535	
ENSG00000225964.6	2749	NRIR	lncRNA	1.34684067	3.094643815	10.11490524	0.004479865	0.214996632	
ENSG00000173402.11	6923	DAG1	protein_coding	1.358510093	3.111011938	17.31124922	0.000437696	0.083493325	
ENSG00000211947.2	430	IGHV3-21	IG_V_gene	1.383122675	5.70616723	8.740067567	0.007503394	0.261971937	
ENSG00000175063.17	1286	UBE2C	protein_coding	1.385376447	2.560068721	11.25968175	0.002976117	0.178444624	
ENSG00000275713.2	2060	HIST1H2BH	protein_coding	1.387046311	5.035337948	29.04613805	2.35599E-05	0.06105687	
ENSG00000136689.18	3256	IL1RN	protein_coding	1.390431369	8.636648323	17.73186027	0.000388006	0.081592857	
ENSG00000272666.1	602	U62317.1	lncRNA	1.391853713	1.261364533	8.224550946	0.009174243	0.279390877	
ENSG00000138642.14	5793	HERC6	protein_coding	1.392591437	6.070843361	15.40153236	0.00077087	0.107678826	
ENSG00000203814.6	3196	HIST2H2BF	protein_coding	1.395805683	3.322985798	14.85890272	0.000910819	0.120022226	
ENSG00000260943.1	1951	LINC02555	lncRNA	1.395955043	2.618303233	9.48687833	0.005650083	0.231803695	
ENSG00000120279.6	3030	MYCT1	protein_coding	1.406325848	3.125195439	16.75534539	0.000514412	0.088431676	
ENSG00000119922.10	4074	IFIT2	protein_coding	1.407475399	11.23473681	9.511713613	0.005597853	0.231803695	
ENSG00000282988.2	2110	AL031777.3	protein_coding	1.407579401	3.983857619	20.50686594	0.00018126	0.065041535	
ENSG00000004468.13	6857	CD38	protein_coding	1.416414109	5.733248933	24.10707287	7.29699E-05	0.065041535	
ENSG00000130487.8	4821	KLHDC7B	protein_coding	1.421955562	3.476889347	8.726806031	0.007541886	0.261971937	
ENSG00000197122.11	6468	SRC	protein_coding	1.4454958	3.601553871	23.23184904	9.03731E-05	0.065041535	
ENSG00000010030.14	2374	ETV7	protein_coding	1.456122573	5.790260311	9.727267123	0.005166323	0.223976443	
ENSG00000274290.2	2585	HIST1H2BE	protein_coding	1.463075451	2.629420104	11.81499461	0.002455502	0.165049355	
ENSG00000115267.8	5094	IFIH1	protein_coding	1.468062742	7.08778823	14.57516491	0.000994956	0.122419737	
ENSG00000244437.1	442	IGKV3-15	IG_V_gene	1.473017788	6.478607057	12.99347705	0.001652732	0.147171585	
ENSG00000211943.2	437	IGHV3-15	IG_V_gene	1.477997832	4.750815177	9.020021092	0.006739549	0.247341607	
ENSG00000002549.12	3785	LAP3	protein_coding	1.486777613	7.593378959	18.55770353	0.000307511	0.080341116	
ENSG00000169679.15	6304	BUB1	protein_coding	1.506674734	3.763119331	8.321961626	0.008829252	0.274577975	
ENSG00000165029.16	11264	ABCA1	protein_coding	1.510091418	6.562774613	36.68745022	5.06296E-06	0.023617708	
ENSG00000264964.2	5142	AP001033.1	lncRNA	1.536057885	1.544609418	10.38027366	0.004068268	0.208000207	
ENSG00000188313.13	4332	PLSCR1	protein_coding	1.543761214	8.331011599	19.81898132	0.000217753	0.070145587	
ENSG00000178999.13	2241	AURKB	protein_coding	1.547084826	2.996062462	8.341746452	0.008760956	0.274282597	
ENSG00000231475.3	428	IGHV4-31	IG_V_gene	1.555612075	3.213957875	8.366672859	0.008675744	0.273839572	
ENSG00000142089.16	1151	IFITM3	protein_coding	1.567832287	9.085839954	22.14965382	0.000118478	0.065041535	
ENSG00000137628.17	6746	DDX60	protein_coding	1.567987594	6.846180663	19.57912631	0.000232317	0.072109261	
ENSG00000253998.3	372	IGKV2-29	IG_V_pseudogene	1.573115451	4.864335598	9.40938482	0.005816556	0.233482257	
ENSG00000173801.17	4942	JUP	protein_coding	1.583986942	5.937939962	11.12055797	0.003124892	0.182669133	
ENSG00000211892.4	2740	IGHG4	IG_C_gene	1.612640606	7.590567486	8.381544216	0.008625344	0.273839572	
ENSG00000157240.3	6963	FZD1	protein_coding	1.615029509	2.22466815	11.67029238	0.002580725	0.167668059	
ENSG00000105048.17	2119	TNNT1	protein_coding	1.621449444	2.257201626	8.958360812	0.006900079	0.250446853	
ENSG00000108771.13	3824	DHX58	protein_coding	1.626990646	6.652165018	13.05914099	0.001617423	0.146488379	
ENSG00000135114.12	3517	OASL	protein_coding	1.651963054	9.060016867	13.19811978	0.0015454	0.14488155	
ENSG00000138646.9	4764	HERC5	protein_coding	1.66112001	7.73622033	10.97151671	0.003293442	0.186043106	
ENSG00000179750.16	1896	APOBEC3B	protein_coding	1.67948354	3.188470735	20.17195108	0.000198111	0.066967231	
ENSG00000270882.2	1876	HIST2H4A	protein_coding	1.72579384	2.816051148	21.25268995	0.000149112	0.065041535	
ENSG00000173369.16	1492	C1QB	protein_coding	1.731217885	3.177727957	12.78808166	0.001768776	0.150649855	
ENSG00000110079.18	2946	MS4A4A	protein_coding	1.734638568	3.636439373	9.517802627	0.00558513	0.231803695	
ENSG00000211941.3	473	IGHV3-11	IG_V_gene	1.758602117	4.141298147	8.130735213	0.0095207	0.283359974	
ENSG00000119917.14	2537	IFIT3	protein_coding	1.775832505	11.12627966	11.77184882	0.002492124	0.166708468	
ENSG00000132530.17	6615	XAF1	protein_coding	1.788156122	6.569855506	17.18477054	0.000453975	0.084035778	
ENSG00000108679.13	3422	LGALS3BP	protein_coding	1.830837403	7.648747463	21.73323438	0.000131743	0.065041535	
ENSG00000157601.14	8616	MX1	protein_coding	1.837103232	9.85256908	11.74374367	0.002516303	0.167668059	
ENSG00000134326.11	4302	CMPK2	protein_coding	1.843908851	6.500397934	14.77080248	0.000936073	0.120216809	
ENSG00000038945.15	7812	MSR1	protein_coding	1.855793991	2.588169339	10.59941201	0.003759782	0.201892848	
ENSG00000185339.8	3122	TCN2	protein_coding	1.859839277	5.789411007	25.3701798	5.39993E-05	0.063559168	
ENSG00000133106.14	4924	EPSTI1	protein_coding	1.875876213	8.163164161	23.67550407	8.10418E-05	0.065041535	
ENSG00000171848.15	9155	RRM2	protein_coding	1.882825479	5.536647106	9.78634212	0.005054579	0.223309078	
ENSG00000111335.12	6264	OAS2	protein_coding	1.900462813	8.998647083	15.4010593	0.000770981	0.107678826	

ENSG00000089685.15	3815	BIRC5	protein_coding	1.91686079	3.134523197	15.02519467	0.000930422	0.120216809
ENSG00000251546.1	376	IGKV1D-39	IG_V_gene	1.922535226	2.668339279	8.269778168	0.009012239	0.277676975
ENSG00000111331.13	8161	OAS3	protein_coding	1.923545075	8.580372118	13.21584635	0.001536471	0.14488155
ENSG00000242076.2	421	IGKV1-33	IG_V_gene	1.942504698	3.788281887	10.23442951	0.004289042	0.212364862
ENSG00000158406.4	2163	HIST1H4H	protein_coding	1.964244711	6.190787942	46.91101328	8.73968E-07	0.006794811
ENSG00000234456.8	17783	MAGI2-AS3	lncRNA	1.967839956	1.859132838	12.5408394	0.001920526	0.152361738
ENSG00000134809.9	776	TIMM10	protein_coding	1.97794531	4.416510881	33.63040106	9.11717E-06	0.035441461
ENSG00000242371.1	398	IGKV1-39	IG_V_gene	1.978572994	5.825670536	12.49976856	0.001947097	0.152472606
ENSG00000130202.10	3742	NECTIN2	protein_coding	1.979370604	2.231631427	9.600538064	0.005415377	0.229770887
ENSG00000224373.3	495	IGHV4-59	IG_V_gene	1.979681811	5.842059898	12.26390774	0.002107757	0.155105376
ENSG00000089127.13	4941	AC004551.1	protein_coding	1.997248003	9.163718219	21.133498	0.0001538	0.065041535
ENSG00000126709.15	842	IFI6	protein_coding	2.007528041	7.720708908	18.41109892	0.000320345	0.080341116
ENSG00000187608.10	867	ISG15	protein_coding	2.009423711	8.115698716	12.45841892	0.001974258	0.152472606
ENSG00000224041.3	396	IGKV3D-15	IG_V_gene	2.011189005	1.360309325	10.5432552	0.003836294	0.203358467
ENSG00000211663.2	377	IGLV3-19	IG_V_gene	2.01314709	7.721178291	10.17042576	0.004390082	0.214235683
ENSG00000123689.6	876	G0S2	protein_coding	2.017572783	3.662083594	19.73806848	0.000222551	0.070145587
ENSG00000211956.2	400	IGHV4-34	IG_V_gene	2.022177501	6.742048869	8.861797051	0.007160062	0.254187648
ENSG00000185745.10	4614	IFIT1	protein_coding	2.08310589	9.261357841	15.73674237	0.000696339	0.102807861
ENSG00000130595.19	2717	TNNT3	protein_coding	2.086226572	1.100083474	11.43257596	0.00280202	0.176157157
ENSG00000211972.2	427	IGHV3-66	IG_V_gene	2.090841655	4.028948922	12.39699956	0.002015377	0.152594033
ENSG00000149131.15	3603	SERPING1	protein_coding	2.112553493	8.156318348	20.19637248	0.000196826	0.066967231
ENSG00000217275.2	499	AL031777.1	processed_pseudogene	2.119293599	2.207856895	12.41388624	0.002003979	0.152594033
ENSG00000211895.5	1422	IGHA1	IG_C_gene	2.124552091	11.3443279	8.888239617	0.007087807	0.252390838
ENSG00000168062.10	2815	BATF2	protein_coding	2.127270007	5.019954155	22.60148053	0.000105721	0.065041535
ENSG00000233030.2	3543	AC243772.2	lncRNA	2.151822147	2.941745797	26.66379002	4.0023E-05	0.063559168
ENSG00000211642.3	567	IGLV10-54	IG_V_gene	2.162849031	3.738894155	8.329113913	0.008804495	0.274540161
ENSG00000134321.12	4722	RSAD2	protein_coding	2.164412715	10.23770374	14.55259363	0.001002007	0.122419737
ENSG00000211669.3	382	IGLV3-10	IG_V_gene	2.17652644	4.86109098	18.28137585	0.000332193	0.080811282
ENSG00000160932.11	2638	LY6E	protein_coding	2.196149473	9.527086653	21.77141921	0.000130461	0.065041535
ENSG00000273133.1	556	AC116651.1	lncRNA	2.234947147	0.850097784	8.175824287	0.009652567	0.283700719
ENSG00000233822.4	5477	HIST1H2BN	protein_coding	2.264747163	2.843176593	12.39300527	0.002018084	0.152594033
ENSG00000122952.17	2377	ZWINT	protein_coding	2.271403095	3.276485605	12.63780405	0.001859366	0.15097794
ENSG00000282122.1	402	IGHV7-4-1	IG_V_gene	2.286092629	5.674696695	8.678605799	0.007683647	0.264326523
ENSG00000137965.11	2038	IFI44	protein_coding	2.323204983	7.532671739	30.67915212	1.66321E-05	0.04849101
ENSG00000211655.3	393	IGLV1-36	IG_V_gene	2.335017555	1.527135356	11.14688544	0.00309612	0.181899001
ENSG00000136514.3	1501	RTP4	protein_coding	2.351678364	5.234735718	25.5165712	5.21768E-05	0.063559168
ENSG00000117399.14	2012	CDC20	protein_coding	2.373722344	3.815517686	14.00998361	0.001270815	0.130531104
ENSG00000196141.14	10453	SPATS2L	protein_coding	2.389429187	5.404157401	11.68358489	0.002568933	0.167668059
ENSG00000137959.16	10240	IFI44L	protein_coding	2.504458271	8.633108872	17.54161275	0.000409668	0.081592857
ENSG00000260000.2	1517	AL133338.1	lncRNA	2.573843766	0.59651096	8.564716486	0.00830614	0.271700571
ENSG00000153208.16	4700	MERTK	protein_coding	2.577498067	2.276877975	10.96475861	0.003301319	0.186043106
ENSG00000161800.13	4487	RACGAP1	protein_coding	2.596461702	1.067019707	8.153408186	0.009737286	0.28424588
ENSG00000184979.9	2129	USP18	protein_coding	2.681097767	6.568931187	11.02816185	0.003228228	0.183646793
ENSG00000211673.2	398	IGLV3-1	IG_V_gene	2.714202102	6.225028559	16.38037344	0.000574465	0.091657831
ENSG00000211967.3	571	IGHV3-53	IG_V_gene	2.721686264	4.17533928	20.97864088	0.000160133	0.065041535
ENSG00000143344.15	5305	RGL1	protein_coding	2.77578243	3.12971193	12.63994285	0.001858041	0.15097794
ENSG00000257452.1	575	AC004551.2	lncRNA	2.806269003	1.184955851	12.99263957	0.001653188	0.147171585
ENSG00000133216.16	12516	EPHB2	protein_coding	2.842979012	2.481057106	13.04179016	0.001626672	0.146488379
ENSG00000094804.12	5311	CDC6	protein_coding	2.865156769	2.634394753	11.13007076	0.003446067	0.190917048
ENSG00000211659.2	380	IGLV3-25	IG_V_gene	2.898996513	7.414105867	21.50523963	0.00013969	0.065041535
ENSG00000167601.12	5134	AXL	protein_coding	2.902439724	2.220806004	9.219298106	0.006755128	0.247341607
ENSG00000078081.8	3785	LAMP3	protein_coding	2.95035247	4.710800997	9.192518287	0.006312013	0.241675201
ENSG00000186889.10	2985	TMEM17	protein_coding	2.979347996	1.389600414	8.805982381	0.007315273	0.258126231
ENSG00000211658.2	377	IGLV3-27	IG_V_gene	2.982752067	3.009585442	17.56953722	0.000406407	0.081592857
ENSG00000075702.18	9814	WDR62	protein_coding	3.048560371	1.15042893	8.441661311	0.008424889	0.272163583
ENSG00000129673.9	2190	AANAT	protein_coding	3.103245768	-0.588424534	9.898170581	0.005055188	0.223309078
ENSG00000186806.5	3771	VSIG10L	protein_coding	3.168710282	0.987133319	10.69051024	0.003639224	0.198125248
ENSG00000124657.1	942	OR2B6	protein_coding	3.234364552	3.779279599	66.77001138	5.59548E-08	0.000652544
ENSG00000246731.3	2621	AC100786.1	lncRNA	3.248684682	0.82005851	8.371636907	0.008946471	0.277114875
ENSG00000287299.1	8982	AC012459.1	lncRNA	3.287069433	1.5420625	20.35239444	0.000188828	0.065734645
ENSG00000216331.2	574	HIST1H1PS1	unprocessed_pseudogene	3.299028573	-1.746273104	9.992733223	0.004684665	0.218094049
ENSG00000274641.1	381	HIST1H2BO	protein_coding	3.303110385	4.161161747	70.81091499	3.45893E-08	0.000652544
ENSG00000211665.3	385	IGLV3-16	IG_V_gene	3.339667932	2.696252623	17.56406448	0.000407044	0.081592857
ENSG00000231686.1	988	Z97180.1	processed_pseudogene	3.339890891	-3.06135119	10.55263217	0.004200479	0.21024027
ENSG00000240518.2	316	AC116353.1	processed_pseudogene	3.343315871	-0.259727051	9.855290069	0.005134399	0.22384061
ENSG00000088827.12	7886	SIGLEC1	protein_coding	3.358248257	6.898946805	19.26871221	0.000252777	0.074629866
ENSG00000230266.1	784	XXYLT1-AS2	lncRNA	3.445156796	-0.041286869	8.445009451	0.008696875	0.274052374
ENSG00000145386.10	2798	CCNA2	protein_coding	3.463489427	2.367156962	21.9905422	0.000123365	0.065041535
ENSG00000237088.1	788	AL163193.1	processed_pseudogene	3.492149515	-0.947363423	8.560550086	0.009831958	0.285712268
ENSG00000171631.14	3976	P2RY6	protein_coding	3.504034486	2.267740907	13.16967817	0.001559847	0.14488155
ENSG00000253686.2	5414	LINC01484	lncRNA	3.512421452	-1.543844623	9.265639123	0.007680846	0.264326523
ENSG00000137474.22	10452	MYO7A	protein_coding	3.52299953	1.631002497	11.31314802	0.002921036	0.177137514
ENSG00000217159.2	292	LARP1P1	processed_pseudogene	3.609198261	-0.979814445	10.30586504	0.004366266	0.213947052
ENSG00000234232.7	3340	AC243772.3	transcribed_unprocessed_pseudogene	3.63083585	-0.45643893	11.86229738	0.002697812	0.172175118
ENSG00000170509.12	2377	HSD17B13	protein_coding	3.653093346	-0.059991569	9.304511709	0.006050555	0.237181767
ENSG00000254285.3	1449	KRT8P3	processed_pseudogene	3.676089832	-0.767649007	8.629462781	0.008752485	0.274282597
ENSG00000211974.3	434	IGHV2-70D	IG_V_gene	3.700537746	-1.560737171	8.191494091	0.009928968	0.286612934
ENSG00000204396.10	3638	VWA7	protein_coding	3.703996319	-0.271506418	9.356002045	0.006165695	0.238884846
ENSG00000183831.6	2911	ANKRD45	protein_coding	3.728131422	-2.698650539	10.96542273	0.004700356	0.218311104
ENSG00000165480.16	2998	SKA3	protein_coding	3.768616127	1.092746329	11.5876317	0.002954922	0.177630443
ENSG00000273007.2	2849	AC021205.3	lncRNA	3.827029715	-1.827210697	9.536976644	0.006999835	0.250789778
ENSG00000221955.10	5586	SLC12A8	protein_coding	3.900223807	0.133132885	12.77071966	0.002315958	0.159849169
ENSG00000261546.1	2105	AC135782.3	lncRNA	3.941466788	-0.984568585	8.260859633	0.009668766	0.283700719
ENSG00000259792.1	483	AC104758.2	lncRNA	3.941612671	-2.355455079	9.258708303	0.00743534	0.261571438
ENSG00000230013.2	3356	CT70	lncRNA	3.944918461	-2.131098286	10.44238413	0.005540471	0.231803695
ENSG00000257953.1	610	AC083805.2	lncRNA	3.965723056	-1.025454895	14.05954339	0.001251319	0.130531104
ENSG00000259268.2	620	AC007950.1	lncRNA	3.980581448	-0.158899645	9.445657196	0.005964693	0.23500085
ENSG00000054690.13	10817	PLEKHH1	protein_coding	4.023418781	-1.577748015	11.13944231	0.004829726	0.220447205
ENSG00000211961.3	411	IGHV1-45	IG_V_gene	4.029347609	-1.989711375	12.1677516	0.003007362	0.178770775
ENSG00000154611.14	1894	PSMA8	protein_coding	4.037832283	-0.967396931	9.226166128	0.006470263	0.243835909
ENSG00000180921.7	6220	FAM83H	protein_coding	4.053731113	-1.358999859	8.586567144	0.008892442	0.276174844
ENSG00000287938.1	512	AC026726.2	lncRNA	4.116530143	-0.74883889	9.309509769	0.006272885	0.241675201
ENSG00000214803.3	1195	AC090921.1	lncRNA	4.162243193	-1.420768021	8.719930713	0.00929141	0.28078551
ENSG00000286216.1	1100	AC104335.1	lncRNA	4.163211384	-2.165389711	10.05550111	0.005544266	0.231803695
ENSG00000165949.12	4147	IFI27	protein_coding	4.164945604	6.409654861	38.03491942	3.94657E-06	0.023012436
ENSG00000261889.1	748	AC108134.2	lncRNA	4.180804323	-1.965866016	10.13595637	0.005394979	0.229770887
ENSG00000213529.3	403	AL359073.1	processed_pseudogene	4.216383107	-1.90506539	10.44206499	0.004598697	0.215832975
ENSG00000288095.1	1508	AC005618.4	lncRNA	4.302691189	-1.733899189	8.784873875	0.009585449	0.283359974
ENSG00000247735.2	1762	AC120114.1	lncRNA	4.338433632	-1.509648769	8.544025824	0.009432681	0.281700196
ENSG00000140323.6	12752	DISP2	protein_coding	4.356803909	-1.612720606	9.805840372	0.007289636	0.257611314
ENSG00000135547.9	2689	HEY2	protein_coding	4.374213742	-1.875976826	9.343061441	0.00793102	0.267703473
ENSG00000108700.5	862	CCL8	protein_coding	4.457009449	0.961822413	9.774194906	0.006875087	0.250446853
ENSG00000162949.16	5191	CAPN13	protein_coding	4.474743857	-0.893073386	10.37817568	0.005654961	0.231803695
ENSG00000163092.19	13031	XIRP2	protein_coding	4.518578597	1.714385454	9.801482555	0.005235753	0.225727722
ENSG00000187987.9	3283	ZSCAN23	protein_coding	4.546846461	-0.852771255	15.66086963	0.001003982	0.122419737
ENSG00000261342.1	1585	AC006538.2	lncRNA	4.560510479	-0.891348205	13.39828125	0.001541236	0.14488155
ENSG00000177294.7	2926	FBXO39	protein_coding	4.580021326	0.038466191	10.48048266	0.004804931	0.219849572
ENSG00000226025.9	2570	AC005515.1	transcribed_unprocessed_pseudogene	4.597978761	-0.618160878	10.22966523	0.004946251	0.22271498
ENSG00000237372.3	3191	AL606807.1	lncRNA	4.606194158	-0.737482127	15.82632544	0.000732605	0.105477085
ENSG00000231528.3	6131	FAM225A	lncRNA	4.609018157	-0.025880414	10.50449322	0.004271248	0.211962973
ENSG00000287011.1	4132	AC003077.1	lncRNA	4.627244244	-0.708166539	9.608588873	0.005617785	0.231803695
ENSG00000164976.9	6447	MYORG	protein_coding	4.632439554	-0.239525869	10.3049222	0.005095976	0.223581476
ENSG00000114698.15	4231	PLSCR4	protein_coding	4.828092393	-0.152018147	11.35115073	0.003612055	0.197764266
ENSG00000115163.15	1949	CENPA	protein_coding	4.862482783	-0.541616364	12.3775911	0.002612917	0.169288001
ENSG00000239207.2	979	GAPDHP39	processed_pseudogene	4.869228796	-1.200913074	8.443434024	0.009377976	0.28078551
ENSG00000183160.9	3184	TMEM119	protein_coding	4.893370128	-0.739613826	11.19368857	0.003800499	0.202380887
ENSG00000132170.21	4545	PPARG	protein_coding	4.929648333	0.709953425	14.84171108	0.001261523	0.130531104
ENSG00000165891.16	6291	E2F7	protein_coding	5.045890504	-0.197757852	13.66671896	0.001516594	0.14488155
ENSG00000135409.11	3656	AMHR2	protein_coding	5.189634782	-1.058223461	15.87580712	0.001051384	0.124479567

ENSG00000278875.1	1802	AC025809.2	TEC	5.2466078	-0.47932121	10.22409279	0.004710844	0.218311104
ENSG00000283384.1	3376	AL138694.1	lncRNA	5.281720086	-0.994466553	10.11385888	0.004897294	0.222659797
ENSG00000162062.15	2848	TEDC2	protein_coding	5.310436248	-0.377134713	8.566506191	0.009355856	0.28078551
ENSG00000128965.13	2051	CHAC1	protein_coding	5.346045166	-0.0012451	11.60455763	0.003330456	0.187179665
ENSG00000227502.3	2755	LINC01268	lncRNA	5.357834031	-0.000272502	11.14204186	0.004450259	0.214458329
ENSG00000287181.1	1745	AC107067.2	lncRNA	5.491399264	-1.136050444	13.92543986	0.004570067	0.215773794
ENSG00000205837.7	2202	LINC00487	lncRNA	5.515300304	0.064881937	29.80943744	4.12661E-05	0.063559168
ENSG00000183722.9	4564	LHFPL6	protein_coding	5.5277603	-0.168637275	11.89025074	0.002844192	0.177002596
ENSG00000115884.11	3613	SDC1	protein_coding	5.568853488	1.910942087	26.14848851	0.00010162	0.065041535
ENSG00000227742.2	2704	CALR4P	transcribed_unitary_pseudogene	5.61580431	-1.373983182	20.89378783	0.000266502	0.077698735
ENSG00000104067.16	11265	TJP1	protein_coding	5.617993763	-0.846885133	15.9458766	0.001157383	0.129462221
ENSG00000259319.1	4059	AF111167.2	lncRNA	5.656386322	0.406713563	17.65701507	0.000433436	0.083493325
ENSG00000246375.3	2591	PPM1K-DT	lncRNA	5.695589778	-1.170099	24.15138111	9.45102E-05	0.065041535
ENSG00000185585.20	6771	OLFML2A	protein_coding	5.767212688	-0.714704927	21.8780796	0.000291228	0.080341116
ENSG00000115155.17	7885	OTOF	protein_coding	6.288569642	3.703149031	24.17855822	8.17335E-05	0.065041535
ENSG00000049130.16	6205	KITLG	protein_coding	6.339772383	-1.02651118	18.70698841	0.001856105	0.15097794
ENSG00000233975.1	441	LINC02574	lncRNA	6.497385309	-1.153826693	18.70004483	0.001858307	0.15097794
ENSG00000151364.17	1899	KCTD14	protein_coding	7.001026545	0.04032819	24.7517518	0.000162089	0.065041535

DEGs, differentially expressed genes; WT, wildtype; SARS-CoV-2, Severe Acute Respiratory Syndrome Coronavirus 2; FC, fold change.

Supplemental Table 6 List of DEGs between whole blood transcriptomes of WT or Δ382 SARS-CoV-2 infected patients during the acute phase of infection, with thresholds of *p*-value < 0.01 and |FC| > 2.

Gene ID	Length (bp)	Gene Name	Gene Type	logFC	logCPM	F	*p*-value	FDR	Remarks
ENSG00000248538.8	17427	AC022784.1	lncRNA	-8.570792028	-0.213414464	24.26154109	0.000128163	0.270495492	Over-expressed in Δ382 SARS-CoV-2 infected patients
ENSG00000116147.17	12806	TNR	protein_coding	-7.728316617	-0.337631061	23.15838911	3.9574E-05	0.224196384	
ENSG00000229981.8	6642	LINC01435	lncRNA	-7.538343155	-1.140063212	18.10968938	0.000533971	0.283914319	
ENSG00000112530.11	2612	PACRG	protein_coding	-6.833557122	-1.70456455	20.56825352	0.000293026	0.270495492	
ENSG00000181109.3	963	OR52P1P	unprocessed_pseudogene	-6.459947823	-0.577876581	24.34133313	2.81016E-05	0.218078169	
ENSG00000119508.18	6314	NR4A3	protein_coding	-6.420483583	-2.02082713	18.93797744	0.000434097	0.270495492	
ENSG00000227658.1	381	AC005160.1	lncRNA	-6.116273006	-2.211125853	17.62858018	0.000603687	0.283914319	
ENSG00000134138.20	10817	MEIS2	protein_coding	-5.992874275	-1.790281861	18.01895962	0.000194084	0.270495492	
ENSG00000135549.15	3432	PKIB	protein_coding	-5.945273426	-1.338158645	25.2061299	2.19899E-05	0.218078169	
ENSG00000166126.10	4646	AMN	protein_coding	-5.699372555	-2.448834467	18.00919785	0.000547752	0.283914319	
ENSG00000266903.2	2430	AC243964.2	lncRNA	-5.599778991	-1.300461956	17.2859643	0.000247223	0.270495492	
ENSG00000271414.1	445	AC093107.2	processed_pseudogene	-5.544331553	-2.556963991	16.26564467	0.000863496	0.319095956	
ENSG00000214548.18	23224	MEG3	lncRNA	-5.493936334	-0.227521344	11.39671096	0.002049598	0.368089868	
ENSG00000262488.1	1161	AC133065.2	processed_pseudogene	-5.376192075	-1.689735075	20.34151232	9.25471E-05	0.270495492	
ENSG00000285486.1	4061	AC003043.2	lncRNA	-5.346369402	-1.06087826	14.78066423	0.000584367	0.283914319	
ENSG00000255418.6	1348	AC090092.1	lncRNA	-5.315748186	-1.892924636	17.47995911	0.000231791	0.270495492	
ENSG00000234460.2	756	AL772337.3	transcribed_unprocessed_pseudogene	-5.266671458	-2.706207653	20.96343304	0.000267131	0.270495492	
ENSG00000287899.1	4878	AC055714.1	lncRNA	-5.26379979	-0.905227388	15.93759437	0.000390201	0.270495492	
ENSG00000236886.2	904	AC007563.2	lncRNA	-5.174111746	-2.770632783	16.88961802	0.000731561	0.304133252	
ENSG00000229521.2	428	MYCBP2-AS2	lncRNA	-5.120311955	-2.781782289	17.45425577	0.000631422	0.288238094	
ENSG00000169169.14	5864	CPT1C	protein_coding	-5.091538737	-1.5243336	14.83940037	0.000572343	0.283914319	
ENSG00000090932.10	2618	DLL3	protein_coding	-5.072456199	-2.483700693	18.261291	0.000179323	0.270495492	
ENSG00000185662.9	1647	SMIM23	protein_coding	-5.071835352	-1.990876782	17.14983809	0.000258707	0.270495492	
ENSG00000200269.1	108	RNU6-838P	snRNA	-5.062102047	-2.804141455	17.15719803	0.000682042	0.295967339	
ENSG00000285763.1	3467	AL358777.1	lncRNA	-5.048763642	-2.070365661	15.9615615	0.000386998	0.270495492	
ENSG00000152931.8	11636	PART1	lncRNA	-5.047649624	-0.650452937	12.14665389	0.001536131	0.3581358	
ENSG00000284664.1	2071	AL161756.3	lncRNA	-5.008004768	-1.784780325	16.17112215	0.00036016	0.270495492	
ENSG00000277297.1	1603	ATP5F1AP10	processed_pseudogene	-5.00470806	-2.860100934	14.85407085	0.00127282	0.357441589	
ENSG00000116774.12	2821	OLFML3	protein_coding	-4.988272664	-0.554058545	14.96902398	0.000546737	0.283914319	
ENSG00000249965.2	563	CDC42P4	processed_pseudogene	-4.97766952	-2.107497528	16.91490072	0.000279893	0.270495492	
ENSG00000138395.15	6165	CDK15	protein_coding	-4.952635647	-0.773982058	12.70875077	0.001242524	0.357441589	
ENSG00000173124.14	2807	ACSM6	protein_coding	-4.938957271	-1.40852176	12.2790551	0.001460821	0.3581358	
ENSG00000243478.9	3835	AOX2P	transcribed_unprocessed_pseudogene	-4.903850335	-1.854898693	12.06536057	0.001584432	0.359777137	
ENSG00000227189.2	2165	AC092535.1	lncRNA	-4.834776785	-1.941767206	14.73316612	0.000594289	0.283914319	
ENSG00000227036.8	27654	LINC00511	lncRNA	-4.794190962	0.507010923	11.61549517	0.001882995	0.365316646	
ENSG00000228407.2	340	AL139156.2	processed_pseudogene	-4.771029722	-2.399701001	13.78451822	0.000835484	0.31886722	
ENSG00000288054.1	3917	AL360178.2	lncRNA	-4.761699322	-1.510438188	12.29245151	0.001453425	0.3581358	
ENSG00000130561.17	11885	SAG	protein_coding	-4.759473479	-0.28966641	12.9129366	0.001151324	0.348103414	
ENSG00000235659.1	439	AL391987.4	lncRNA	-4.703824073	-2.954364683	16.16770131	0.000886537	0.322491829	
ENSG00000267218.2	939	AC005336.2	processed_pseudogene	-4.702976683	-2.98856639	14.51740836	0.001400138	0.3581358	
ENSG00000197889.10	1464	MEIG1	protein_coding	-4.670726667	-1.838728664	13.53561387	0.000914899	0.327210924	
ENSG00000036530.8	9352	CYP46A1	protein_coding	-4.606724907	-1.923742848	10.49936007	0.002918958	0.427397877	
ENSG00000229695.1	1158	AC011242.1	processed_pseudogene	-4.601754853	-1.924855308	13.72308738	0.000854371	0.319095956	
ENSG00000198963.11	9621	RORB	protein_coding	-4.600219567	-0.604262037	10.56756337	0.002840593	0.426986635	
ENSG00000261404.7	7473	AC138627.1	lncRNA	-4.581617347	-0.795217726	9.535316841	0.004315422	0.475142056	
ENSG00000228476.2	1136	CSTP1	transcribed_unprocessed_pseudogene	-4.526278378	-2.386359524	12.70878519	0.001242508	0.357441589	
ENSG00000248885.1	1271	AC118465.1	processed_pseudogene	-4.470818042	-2.390139074	13.04854201	0.001094745	0.346162446	
ENSG00000214917.3	388	AC011825.1	processed_pseudogene	-4.445474414	-2.557458799	14.73126651	0.000594689	0.283914319	
ENSG00000159708.18	4018	LRRC36	protein_coding	-4.402065729	-0.918432858	9.950489695	0.003641342	0.467897063	
ENSG00000251396.7	9858	LINC01301	lncRNA	-4.388569277	-1.075558517	9.346319896	0.004665869	0.477827571	
ENSG00000235258.1	366	NDUFB4P6	processed_pseudogene	-4.382733413	-2.744027093	19.19257043	0.000132853	0.270495492	
ENSG00000186453.13	2281	FAM228A	protein_coding	-4.380059624	-2.624106972	15.02499199	0.000536063	0.283914319	
ENSG00000203910.9	1086	C1orf146	protein_coding	-4.37927365	-2.848530543	21.18815534	7.13109E-05	0.270495492	
ENSG00000136546.16	9733	SCN7A	protein_coding	-4.359712315	-1.929060482	10.40011545	0.00303718	0.43194914	
ENSG00000164744.13	1732	SUN3	protein_coding	-4.34243708	-2.089420034	11.75309214	0.001785719	0.365293192	
ENSG00000187527.10	6228	ATP13A5	protein_coding	-4.31160639	-1.830383874	10.88334404	0.002506158	0.399629217	
ENSG00000261183.5	1239	SPINT1-AS1	lncRNA	-4.307392179	-1.957178415	13.86339407	0.000811888	0.315026083	
ENSG00000234335.1	772	RPS4XP11	processed_pseudogene	-4.290872103	-2.343822553	13.26353677	0.001011073	0.341142005	
ENSG00000287067.1	3018	AC106791.3	transcribed_processed_pseudogene	-4.282794842	-2.083104325	10.76719167	0.002623962	0.407256321	
ENSG00000254101.7	25498	LINC02055	lncRNA	-4.28044474	-0.52521253	8.123946936	0.007826234	0.562824908	
ENSG00000230370.1	478	RPL23AP52	processed_pseudogene	-4.271704585	-1.999658816	11.17196187	0.002237385	0.380208425	
ENSG00000176177.9	3068	ENTHD1	protein_coding	-4.243146957	-2.043834918	10.56562592	0.002842787	0.426986635	
ENSG00000204385.12	3372	SLC44A4	protein_coding	-4.241192062	-1.390182698	9.353267165	0.004652457	0.477827571	
ENSG00000104112.9	3171	SCG3	protein_coding	-4.239113711	-2.410024494	11.51800961	0.001955356	0.368089868	
ENSG00000177788.6	8197	AL162595.1	lncRNA	-4.232492605	-0.659420685	8.445510554	0.006816146	0.553070305	
ENSG00000224565.2	1216	LINC01754	lncRNA	-4.232468249	-1.81379939	11.08902171	0.002311293	0.384351563	
ENSG00000215386.13	42926	MIR99AHG	lncRNA	-4.222529654	0.181308087	9.801384115	0.003869359	0.47483434	
ENSG00000176601.13	8130	MAP3K19	protein_coding	-4.218384611	-1.719220729	9.482227979	0.004410899	0.475250539	
ENSG00000272699.1	497	AC007620.3	lncRNA	-4.21835912	-2.359871526	13.97514391	0.000779674	0.313377115	
ENSG00000163106.10	2111	HPGDS	protein_coding	-4.209921964	-2.013434894	11.95355954	0.001653544	0.359777137	
ENSG00000275343.1	324	AC010999.1	lncRNA	-4.209447209	-3.175506633	13.20116362	0.002055396	0.368089868	
ENSG00000272070.1	3148	AC005618.1	lncRNA	-4.205026157	-0.339074256	10.25580103	0.003218389	0.435623926	
ENSG00000278909.1	3365	AC007608.4	TEC	-4.19083043	-1.736615632	10.29213346	0.003171696	0.432446069	
ENSG00000139610.1	952	CELA1	protein_coding	-4.172011444	-1.678415911	11.67987223	0.001836791	0.365293192	
ENSG00000232490.6	2307	OSBPL10-AS1	lncRNA	-4.170575285	-1.788338151	9.738523655	0.003970061	0.47483434	
ENSG00000144452.15	9614	ABCA12	protein_coding	-4.092807448	-1.173278155	8.782577029	0.00590682	0.523190066	
ENSG00000222448.1	321	RN7SKP150	misc_RNA	-4.085215844	-2.583689089	12.14291453	0.001538318	0.3581358	
ENSG00000230337.1	536	EXOSC10-AS1	lncRNA	-4.074222552	-1.676965441	9.868940037	0.003764198	0.468632535	
ENSG00000274776.1	2599	AC090241.3	lncRNA	-4.072446436	-1.976848151	11.95903292	0.001650086	0.359777137	
ENSG00000174844.14	13275	DNAH12	protein_coding	-4.06168752	0.037769151	11.13163952	0.002272993	0.380701866	
ENSG00000228835.1	1661	AC012123.1	lncRNA	-4.055095147	-2.411441983	12.64130745	0.001274329	0.357441589	
ENSG00000273507.5	15932	AL354809.1	lncRNA	-4.043139729	-0.938711593	8.611770693	0.006350017	0.531779675	
ENSG00000230894.2	5550	AL133284.1	lncRNA	-4.042954712	-0.693887742	8.745442939	0.006000247	0.523190066	
ENSG00000249784.1	125	SCARNA22	scaRNA	-4.036116247	-2.682280533	13.38799152	0.000965798	0.33559317	
ENSG00000225632.1	1111	AL161644.1	lncRNA	-4.034482808	-1.898801327	11.9575236	0.001651039	0.359777137	
ENSG00000229593.1	1357	SUCLA2P3	processed_pseudogene	-4.026920629	-1.993842328	10.04159347	0.003509186	0.46156699	
ENSG00000176239.7	939	OR51B6	protein_coding	-3.998576862	-1.828045415	12.68094105	0.001255534	0.357441589	
ENSG00000230750.1	2028	SDAD1P2	processed_pseudogene	-3.993883132	-1.747947699	8.41021374	0.006919793	0.554367113	
ENSG00000165105.10	6644	RASEF	protein_coding	-3.982712035	-2.716366322	10.01902661	0.003541433	0.463191527	
ENSG00000231728.4	2767	TMSB15B-AS1	lncRNA	-3.945502851	-2.785310517	12.07311683	0.001579754	0.359777137	
ENSG00000261916.1	512	AC027796.1	lncRNA	-3.923272065	-2.411754562	10.50581744	0.00291144	0.427397877	
ENSG00000285807.1	555	AL353768.2	lncRNA	-3.908082552	-2.887701574	11.05931734	0.002338399	0.386036623	
ENSG00000172139.15	4208	SLC9C1	protein_coding	-3.87847336	-0.867733607	9.639463492	0.004134538	0.47483434	
ENSG00000053328.8	1774	METTL24	protein_coding	-3.856576858	-2.293027684	11.03704059	0.002358951	0.386036623	
ENSG00000287164.1	597	AC112254.1	lncRNA	-3.844111557	-2.666116871	11.55741915	0.001925749	0.367486642	
ENSG00000260765.2	1242	CES1P2	unprocessed_pseudogene	-3.832210032	-2.114134437	9.766673124	0.00392462	0.47483434	
ENSG00000236582.1	851	PRPF38AP2	processed_pseudogene	-3.800132747	-1.962955271	8.638348925	0.006278754	0.529049382	
ENSG00000236015.1	823	AC011290.2	processed_pseudogene	-3.792523132	-1.685294731	10.5953321	0.002809338	0.426986635	
ENSG00000287322.1	1551	AC068189.2	lncRNA	-3.766332988	-2.24797417	10.38149735	0.003059927	0.43194914	
ENSG00000249604.1	3147	AC096564.2	lncRNA	-3.766219856	-2.212307926	8.373987897	0.007027944	0.556519401	
ENSG00000117425.14	5334	PTCH2	protein_coding	-3.762371303	-0.620759074	10.54066927	0.00287122	0.427397877	
ENSG00000211611.2	406	IGKV6-21	IG_V_gene	-3.759410722	4.497344157	10.44501532	0.002983067	0.431358951	
ENSG00000286305.1	3000	AC004893.3	lncRNA	-3.738288233	-2.190209477	9.384746973	0.004592202	0.477827571	
ENSG00000215035.2	1057	FDPSP5	processed_pseudogene	-3.733460734	-2.014161227	8.545783822	0.006530756	0.541076649	
ENSG00000131126.18	1597	TEX101	protein_coding	-3.730337105	-1.544354488	8.208255011	0.007546609	0.558519023	
ENSG00000285881.1	1235	AC105206.3	lncRNA	-3.716255694	-1.675980334	8.193837475	0.00759365	0.558519023	
ENSG00000286482.1	3835	AL117344.2	lncRNA	-3.714901197	-1.550881225	8.033503796	0.008138734	0.567161075	
ENSG00000224437.2	505	PIGUP1	processed_pseudogene	-3.711266778	-2.379865904	7.728576579	0.009296071	0.585027465	
ENSG00000216365.2	287	RPL37P15	processed_pseudogene	-3.710116653	-2.426588362	8.964197388	0.005471976	0.511618749	
ENSG00000229740.3	1716	U91324.1	lncRNA	-3.674291783	-2.560335183	11.22370448	0.002192568	0.377876537	
ENSG00000159871.15	5329	LYPD5	protein_coding	-3.652057219	-0.283126543	10.21750765	0.003268405	0.439836651	
ENSG00000259617.1	500	AC020661.3	lncRNA	-3.65135236	-2.312434672	9.659225512	0.004101148	0.47483434	
ENSG00000114654.7	2945	EFCC1	protein_coding	-3.648086742	-2.478815596	8.833775737	0.005780576	0.523190066	
ENSG00000080618.16	1724	CPB2	protein_coding	-3.638345823	-2.711426962	9.421368701	0.004523164	0.477827571	
ENSG00000074527.12	4166	NTN4	protein_coding	-3.630900308	-0.203356055	9.57431896	0.004246702	0.475142056	
ENSG00000244538.1	255	AC098583.1	processed_pseudogene	-3.625454863	-1.201662523	9.294193943	0.004767843	0.477827571	
ENSG00000196301.3	343	HLA-DRB9	unprocessed_pseudogene	-3.621725807	-2.771998018	14.66079208	0.000609755	0.283914319	
ENSG00000227544.10	6724	AC018647.1	lncRNA	-3.612839807	-1.21274227	8.511950068	0.006625579	0.5469862	
ENSG00000224137.1	492	LINC01857	lncRNA	-3.607787366	-2.247512981	9.899201507	0.003718094	0.467897063	
ENSG00000257074.3	316	RPL29P33	processed_pseudogene	-3.605303282	-2.64164081	10.80752397	0.002582394	0.403494694	
ENSG00000197149.5	873	AC107956.1	processed_pseudogene	-3.589454501	-2.148686679	8.711203809	0.006087807	0.528844198	
ENSG00000236499.2	2128	LINC00896	lncRNA	-3.587089722	-2.440787749	11.95420067	0.001653138	0.359777137	
ENSG00000273143.2	3430	AL355512.1	lncRNA	-3.584878614	-0.68368765	7.63609704	0.009681449	0.601050191	
ENSG00000235109.7	4957	ZSCAN31	protein_coding	-3.576286376	-0.294786013	8.858773282	0.005720001	0.522224876	
ENSG00000263887.7	1500	AC053481.2	transcribed_unprocessed_pseudogene	-3.562624057	-2.233397646	8.305709868	0.007236791	0.556519401	
ENSG00000204248.10	6740	COL11A2	protein_coding	-3.551987491	-1.978528312	8.270739641	0.007346351	0.557102289	
ENSG00000224232.1	678	AC005104.2	processed_pseudogene	-3.551911223	-3.181175588	12.31960897	0.001438555	0.3581358	
ENSG00000261723.1	627	AC008870.4	lncRNA	-3.545507056	-2.709240994	9.501295261	0.004376348	0.475142056	
ENSG00000267345.1	572	AC010632.1	lncRNA	-3.545413566	-2.955075202	11.34944537	0.002087634	0.368198491	
ENSG00000158859.10	10338	ADAMTS4	protein_coding	-3.536757876	-0.944396842	8.171307419	0.007667798	0.558519023	
ENSG00000279762.3	3449	AC005899.8	lncRNA	-3.525197834	-1.846301842	7.741185965	0.009244828	0.585027465	
ENSG00000266365.1	843	AC053481.3	unprocessed_pseudogene	-3.52492869	-2.313231732	8.154605529	0.007723271	0.558519023	

ENSG00000232642.2	1190	AC008073.2	lncRNA	-3.50461094	-2.873226916	11.67966752	0.001836936	0.365293192	
ENSG00000223915.1	556	DPPA2P1	processed_pseudogene	-3.500814996	-2.744540181	9.514988645	0.004351715	0.475142056	
ENSG00000230863.3	2159	AL137803.1	transcribed_processed_pseudogene	-3.493684635	-2.637975698	9.764556667	0.003928017	0.47483434	
ENSG00000251990.1	101	RNA5SP180	rRNA_pseudogene	-3.485499692	-2.432372672	8.299459575	0.007256243	0.556519401	
ENSG00000266079.4	701	SNORA59B	snoRNA	-3.47090553	-2.626807184	8.412875748	0.006911917	0.554367113	
ENSG00000259144.2	2104	RANBP20P	processed_pseudogene	-3.457760974	-2.011445456	7.876642953	0.008713253	0.582086894	
ENSG00000148483.9	5515	TMEM236	protein_coding	-3.456746788	-1.587651867	8.329078933	0.007164564	0.556519401	
ENSG00000231310.3	855	TBL1XR1-AS1	lncRNA	-3.45485447	-2.050239078	9.901142007	0.003715159	0.467897063	
ENSG00000222741.1	99	RNA5SP229	rRNA_pseudogene	-3.450639816	-3.199365793	14.1935167	0.000720616	0.304133252	
ENSG00000265315.1	281	RN7SL199P	misc_RNA	-3.44625226	-3.199260077	18.01876185	0.000194096	0.270495492	
ENSG00000255524.7	2338	NPIPB8	protein_coding	-3.441695984	-2.805476681	9.265739015	0.004824523	0.477956278	
ENSG00000285416.1	763	AC137630.5	unitary_pseudogene	-3.424167672	-2.682835632	8.747029215	0.005996223	0.523190066	
ENSG00000286613.1	870	AC006213.7	lncRNA	-3.419983781	-2.356613324	8.444854759	0.006818057	0.553070305	
ENSG00000224142.1	561	AMMECR1-IT1	lncRNA	-3.419620719	-3.198141578	13.89124418	0.000803728	0.315026083	
ENSG00000253534.1	341	TRBV6-8	TR_V_gene	-3.407048411	-2.704732306	10.99177315	0.002401315	0.388229279	
ENSG00000278513.1	665	AC091046.2	unprocessed_pseudogene	-3.403385216	-2.225629809	7.77157247	0.009122594	0.585027465	
ENSG00000255867.1	1308	DENND5B-AS1	lncRNA	-3.381137297	-2.32029757	9.117226374	0.005132402	0.489493645	
ENSG00000162482.5	1215	AKR7A3	protein_coding	-3.380414522	-2.136965433	11.9963028	0.001626746	0.359777137	
ENSG00000220744.1	891	RPL5P18	processed_pseudogene	-3.365062231	-2.665915533	9.747379797	0.003955704	0.47483434	
ENSG00000181798.3	1313	LINC00471	lncRNA	-3.364662662	-2.170551529	7.589202959	0.009883458	0.60688206	
ENSG00000227081.5	255	AC005912.1	processed_pseudogene	-3.362017316	2.379059723	10.70955831	0.002684616	0.411187747	
ENSG00000243680.1	294	RPL37P23	processed_pseudogene	-3.355859125	-1.847161317	8.024706755	0.008169843	0.567161075	
ENSG00000287431.1	6784	AC027601.6	lncRNA	-3.30694095	-2.636633319	8.03173061	0.008144994	0.567161075	
ENSG00000265750.1	735	AC090772.3	lncRNA	-3.278760085	-2.421517968	7.811500082	0.008964636	0.585027465	
ENSG00000198723.11	2923	TEX45	protein_coding	-3.278581116	-2.532283233	9.286964489	0.004782175	0.477827571	
ENSG00000236885.2	1821	AC018731.1	lncRNA	-3.267835529	-3.410643928	10.7905782	0.004371372	0.475142056	
ENSG00000236456.1	585	AL035458.1	processed_pseudogene	-3.259477341	-3.413381397	13.61965949	0.001815606	0.365293192	
ENSG00000242088.1	252	AC090602.1	processed_pseudogene	-3.25777857	0.63377692	9.902321047	0.003713377	0.467897063	
ENSG00000238193.1	1415	AC112656.1	processed_pseudogene	-3.250802718	-1.987444709	9.624075834	0.004160741	0.47483434	
ENSG00000231475.3	428	IGHV4-31	IG_V_gene	-3.240488365	5.463191416	8.087241687	0.007951462	0.564384134	
ENSG00000167618.10	1044	LAIR2	protein_coding	-3.225301769	3.838688016	15.76117857	0.000414674	0.270495492	
ENSG00000241923.2	622	RPL14P3	processed_pseudogene	-3.191798621	-2.749617375	8.280764843	0.00731476	0.556519401	
ENSG00000273687.1	332	AC004223.4	lncRNA	-3.173690342	-2.210633256	7.932872887	0.008502404	0.577097596	
ENSG00000240231.1	252	RPS27P29	processed_pseudogene	-3.169668299	0.463643754	7.902775627	0.008614566	0.57813851	
ENSG00000234287.1	255	AC099560.2	processed_pseudogene	-3.166174337	0.977447135	9.643343445	0.00412796	0.47483434	
ENSG00000271153.1	454	RPL23AP88	processed_pseudogene	-3.146959569	-2.486053419	8.103422186	0.007895993	0.563885332	
ENSG00000286485.1	2118	Z80897.1	lncRNA	-3.128695881	-3.012368771	9.694562653	0.004042164	0.47483434	
ENSG00000259917.1	1570	HNRNPLP2	unprocessed_pseudogene	-3.12146937	-3.44060706	11.98278578	0.002983006	0.431358951	
ENSG00000166926.8	1057	MS4A6E	protein_coding	-3.108582517	-2.87108677	9.297440676	0.004761422	0.477827571	
ENSG00000177954.14	1028	RPS27	protein_coding	-3.09928875	8.584411977	12.51248875	0.001337535	0.3581358	
ENSG00000238832.1	145	RF01233	snoRNA	-3.078913015	-3.136531571	8.695151323	0.006129334	0.529049382	
ENSG00000187172.15	4080	BAGE2	transcribed_unprocessed_pseudogene	-3.070446496	-1.495618309	7.847090355	0.008826335	0.585027465	
ENSG00000236229.1	1541	VEZF1P1	processed_pseudogene	-3.069905916	-2.016428694	7.800013326	0.009009773	0.585027465	
ENSG00000241458.1	618	RPL7P19	processed_pseudogene	-3.064749938	-2.229822106	7.768619432	0.009134395	0.585027465	
ENSG00000225082.2	278	DAP3P1	unprocessed_pseudogene	-3.061706128	-2.687560984	7.796317641	0.009024347	0.585027465	
ENSG00000235605.1	438	AL355472.1	processed_pseudogene	-3.027141375	-3.126039156	10.90995711	0.002479977	0.398181627	
ENSG00000170954.11	3780	ZNF415	protein_coding	-3.022528278	0.199753004	12.39262158	0.001399382	0.3581358	
ENSG00000198918.8	2128	RPL39	protein_coding	-3.021184562	5.276816851	10.74232378	0.00264995	0.40856611	
ENSG00000224217.1	348	BX119904.1	processed_pseudogene	-3.018916522	-3.457838269	13.27251811	0.002012107	0.368089868	
ENSG00000223916.1	515	AC097638.1	processed_pseudogene	-3.016588626	-3.463328117	11.39834871	0.003589314	0.466831446	
ENSG00000225832.1	332	AL035090.1	processed_pseudogene	-2.992416289	-3.060567332	8.873464152	0.005684722	0.521073203	
ENSG00000259032.2	352	ENSAP2	processed_pseudogene	-2.985732752	-2.903676592	8.404815063	0.006935796	0.554367113	
ENSG00000227063.5	81	RPL41P1	processed_pseudogene	-2.972887499	2.748760763	9.96015031	0.003627077	0.467897063	
ENSG00000197748.12	7184	CFAP43	protein_coding	-2.972491574	-0.482956763	8.154120548	0.007724888	0.558519023	
ENSG00000206178.2	429	HBZP1	unprocessed_pseudogene	-2.91156802	-3.117101953	8.014957989	0.008204469	0.567161075	
ENSG00000240785.2	641	RPL36AP21	transcribed_processed_pseudogene	-2.890460413	-1.47999704	9.30498107	0.004746545	0.477827571	
ENSG00000256393.1	78	RPL41P5	processed_pseudogene	-2.852287834	2.291054699	9.625378608	0.004158515	0.47483434	
ENSG00000197061.4	435	HIST1H4C	protein_coding	-2.851932193	1.494286406	12.99874647	0.001115166	0.346162446	
ENSG00000279488.1	2127	AC004623.1	TEC	-2.817998882	-3.306752454	8.312553042	0.00721556	0.556519401	
ENSG00000279599.1	208	AC005514.1	TEC	-2.798850468	-3.25096458	8.950944331	0.005502507	0.512415437	
ENSG00000267673.6	1371	FDX2	protein_coding	-2.793230147	-3.244893977	9.367076015	0.004625921	0.477827571	
ENSG00000279483.2	75	AC090498.1	processed_pseudogene	-2.764421896	2.867832532	8.588506271	0.006413114	0.533227494	
ENSG00000271711.1	616	AC069236.1	processed_pseudogene	-2.754083769	-2.891314867	8.282030405	0.007310783	0.556519401	
ENSG00000143185.4	562	XCL2	protein_coding	-2.749000694	2.347074671	13.01306334	0.001109253	0.346162446	
ENSG00000213741.10	9845	RPS29	protein_coding	-2.734200854	5.987080967	12.4542895	0.001367189	0.3581358	
ENSG00000271662.1	1070	AC233280.2	processed_pseudogene	-2.730129539	-3.367054337	9.318657826	0.004719688	0.477827571	
ENSG00000199313.1	140	RNU4-82P	snRNA	-2.653825152	-3.397723108	9.932496095	0.003668073	0.467897063	
ENSG00000244398.1	321	AC116533.1	processed_pseudogene	-2.642676573	0.518409459	8.018647034	0.008191347	0.567161075	
ENSG00000200170.1	101	RF00019	misc_RNA	-2.629131222	-3.277836417	8.398991318	0.006953103	0.554367113	
ENSG00000219553.2	723	AL031133.1	processed_pseudogene	-2.623251077	-3.276166809	8.658694115	0.006224787	0.529049382	
ENSG00000196683.10	1751	TOMM7	protein_coding	-2.592673017	3.995639644	11.71893626	0.001809351	0.365293192	
ENSG00000211831.2	60	TRAJ61	TR_J_gene	-2.546638687	-3.076937107	8.116086163	0.007852872	0.562824908	
ENSG00000071082.11	5277	RPL31	protein_coding	-2.539806146	8.373814717	9.028433024	0.005326568	0.503707486	
ENSG00000229117.9	1108	RPL41	protein_coding	-2.485981008	6.855671249	8.873345919	0.005685005	0.521073203	
ENSG00000206177.7	912	HBM	protein_coding	-2.430193608	5.796961833	11.44260398	0.002013379	0.368089868	
ENSG00000163993.7	471	S100P	protein_coding	-2.341038861	4.706196379	8.043821558	0.00810241	0.567161075	
ENSG00000179766.19	6291	ATP8B5P	transcribed_unprocessed_pseudogene	-2.321610562	-0.6084867	8.305721145	0.007236756	0.556519401	
ENSG00000211713.3	365	TRBV6-4	TR_V_gene	-2.298449832	0.609716647	8.466589239	0.006755052	0.551804815	
ENSG00000260979.1	427	AC022167.3	lncRNA	-2.269981933	0.647226022	9.607412837	0.004189317	0.475142056	
ENSG00000182899.17	2899	RPL35A	protein_coding	-2.250961106	5.627302369	10.38320776	0.00305783	0.43194914	
ENSG00000171858.18	1036	RPS21	protein_coding	-2.187478123	4.129673123	7.919574658	0.008551765	0.577167759	
ENSG00000145592.14	8040	RPL37	protein_coding	-2.167759655	6.449836122	11.02389341	0.002371171	0.386036623	
ENSG00000127922.9	11490	SEM1	protein_coding	-2.166559786	4.472969008	9.705560129	0.004023995	0.47483434	
ENSG00000228474.6	600	OST4	protein_coding	-2.158403823	6.212700805	7.827094492	0.00890375	0.585027465	
ENSG00000149806.11	1548	FAU	protein_coding	-2.124008118	6.515434254	8.013449784	0.00820984	0.567161075	
ENSG00000235576.1	243	LINC01871	lncRNA	-2.098538712	2.195890625	9.494882255	0.004387936	0.475142056	
ENSG00000113088.5	1570	GZMK	protein_coding	-2.095750677	3.306555316	14.32862666	0.000686493	0.295967339	
ENSG00000150045.12	1260	KLRF1	protein_coding	-2.082772657	4.829753073	8.051849074	0.008074271	0.567161075	
ENSG00000086506.3	528	HBQ1	protein_coding	-1.956058789	3.6221037	7.814956944	0.008951101	0.585027465	
ENSG00000165502.6	746	RPL36AL	protein_coding	-1.945259061	5.870939263	9.242928014	0.004870486	0.478114763	
ENSG00000111796.3	1448	KLRB1	protein_coding	-1.885884513	4.023813107	7.981014777	0.008326274	0.569568429	
ENSG00000112306.8	639	RPS12	protein_coding	-1.851717938	6.421361783	7.659710545	0.009581427	0.599637657	
ENSG00000118181.11	1477	RPS25	protein_coding	-1.824367044	6.405484792	9.169436447	0.005021814	0.485115525	
ENSG00000142676.14	2659	RPL11	protein_coding	-1.81495983	7.328890971	10.32063473	0.003135579	0.43194914	
ENSG00000126264.9	613	HCST	protein_coding	-1.810315556	1.971861066	12.22159407	0.001493006	0.3581358	
ENSG00000144713.12	3720	RPL32	protein_coding	-1.809489442	6.725971213	8.303829735	0.007242637	0.556519401	
ENSG00000110700.7	2115	RPS13	protein_coding	-1.809393853	6.633008849	8.16555813	0.007686845	0.558519023	
ENSG00000197756.9	6841	RPL37A	protein_coding	-1.75147124	5.75141048	15.73041579	0.000419107	0.270495492	
ENSG00000243147.8	1532	MRPL33	protein_coding	-1.71377591	3.52838527	8.235328697	0.007459124	0.558519023	
ENSG00000185710.9	6290	SMG1P4	transcribed_unprocessed_pseudogene	-1.707265918	0.066003329	8.761515403	0.005959615	0.523190066	
ENSG00000198242.14	2232	RPL23A	protein_coding	-1.696802148	5.488233765	11.8386412	0.001727974	0.365293192	
ENSG00000008988.9	3002	RPS20	protein_coding	-1.694563944	5.830983493	7.763023316	0.009156806	0.585027465	
ENSG00000090266.13	4447	NDUFB2	protein_coding	-1.680065268	4.124129869	8.286669785	0.007296222	0.556519401	
ENSG00000169413.3	844	RNASE6	protein_coding	-1.666055787	6.552363627	12.93832666	0.001140496	0.348103414	
ENSG00000100079.7	833	LGALS2	protein_coding	-1.665359328	3.915731835	9.206052282	0.004945792	0.479762477	
ENSG00000150540.14	4756	HNMT	protein_coding	-1.646580074	3.738601892	8.599660947	0.006382776	0.532607225	
ENSG00000185808.13	7923	PIGP	protein_coding	-1.627659449	2.468409892	9.289932001	0.004776287	0.477827571	
ENSG00000221983.7	4306	UBA52	protein_coding	-1.588928916	8.086579908	9.834871753	0.003816839	0.472658705	
ENSG00000116675.16	6958	DNAJC6	protein_coding	-1.541601585	1.655977114	8.217215388	0.007517532	0.558519023	
ENSG00000177600.9	1637	RPLP2	protein_coding	-1.445926682	6.253812188	10.35103145	0.003097547	0.43194914	
ENSG00000140264.19	7347	SERF2	protein_coding	-1.422830149	6.375788571	8.093417287	0.007930242	0.564384134	
ENSG00000172809.13	3829	RPL38	protein_coding	-1.422300568	4.665251772	13.07777725	0.001082943	0.346162446	
ENSG00000164587.13	5082	RPS14	protein_coding	-1.409380318	7.244915844	8.643808029	0.006264224	0.529049382	
ENSG00000103363.14	1128	ELOB	protein_coding	-1.396590315	5.215607455	8.178293915	0.007644722	0.558519023	
ENSG00000172428.11	2037	COPS9	protein_coding	-1.359926848	1.761025973	7.733555811	0.009275799	0.585027465	
ENSG00000197956.10	971	S100A6	protein_coding	-1.309952236	7.758619357	7.653089069	0.009609361	0.599773544	
ENSG00000197728.11	2053	RPS26	protein_coding	-1.307711528	4.660336114	9.28737219	0.004781366	0.477827571	
ENSG00000218175.2	345	AC016739.1	processed_pseudogene	-1.282559866	0.989850273	8.204658055	0.007558316	0.558519023	
ENSG00000130255.13	2271	RPL36	protein_coding	-1.27538182	5.403015823	10.10968746	0.003413788	0.451570404	
ENSG00000168060.16	4048	NAALADL1	protein_coding	-1.228197862	1.952905392	8.283788729	0.007305261	0.556519401	
ENSG00000169251.12	4869	NMD3	protein_coding	-1.171341336	3.99288364	8.764646355	0.005951734	0.523190066	
ENSG00000137818.12	3252	RPLP1	protein_coding	-1.118772039	7.69811701	8.825032995	0.005801926	0.523190066	
ENSG00000197958.13	2453	RPL12	protein_coding	-1.113098459	6.300113169	7.601584513	0.009829681	0.60688206	
ENSG00000166441.13	6816	RPL27A	protein_coding	-1.107297501	7.012697597	10.11033382	0.003412896	0.451570404	
ENSG00000128513.16	8717	POT1	protein_coding	-1.048719402	3.473002349	7.999365655	0.008260181	0.568950485	
ENSG00000169740.14	1460	ZNF32	protein_coding	-1.032731061	3.141845673	7.97652089	0.008342547	0.569568429	
ENSG00000146066.3	654	HIGD2A	protein_coding	-1.002728821	3.992084732	9.501404538	0.004376151	0.475142056	
ENSG00000232125.4	2356	DYTN	protein_coding	1.062200495	2.913868104	7.81857888	0.008936942	0.585027465	Under-expressed in Δ382 SARS-CoV-2 infected patients
ENSG00000131462.8	2408	TUBG1	protein_coding	1.116728917	4.711631935	9.344853634	0.004668705	0.477827571	
ENSG00000135069.14	2206	PSAT1	protein_coding	1.189800172	3.433935864	7.728172667	0.009297718	0.585027465	
ENSG00000148773.14	12845	MKI67	protein_coding	1.199007714	4.85293722	8.920435431	0.005573492	0.514906589	
ENSG00000212916.4	3516	MAP10	protein_coding	1.211033944	2.084738601	7.572755812	0.009955388	0.60688206	
ENSG00000124657.1	942	OR2B6	protein_coding	1.40691662	4.0814731	8.346868633	0.007110104	0.556519401	
ENSG00000169679.15	6304	BUB1	protein_coding	1.554544117	3.805521242	8.037012963	0.00812636	0.567161075	

ENSG00000260920.2	2449	AL031985.3	lncRNA	2.029149004	1.33702506	9.238499794	0.004879464	0.478114763
ENSG00000171241.9	6175	SHCBP1	protein_coding	2.135254387	3.255265531	8.993808666	0.00540442	0.507339925
ENSG00000211649.3	385	IGLV7-46	IG_V_gene	2.243142941	3.322432067	9.140760927	0.00508223	0.488277743
ENSG00000239951.1	400	IGKV3-20	IG_V_gene	2.265962393	7.624277371	8.63350929	0.006291665	0.529049382
ENSG00000244437.1	442	IGKV3-15	IG_V_gene	2.319624664	6.626368575	13.97144964	0.000780717	0.313377115
ENSG00000211652.2	385	IGLV7-43	IG_V_gene	2.320727311	2.220255543	7.756409932	0.009183367	0.585027465
ENSG00000225972.1	372	MTND1P23	unprocessed_pseudogene	2.437491117	1.815625885	7.919237556	0.008553021	0.577167759
ENSG00000172232.10	1782	AZU1	protein_coding	2.444971733	1.84805671	8.254760764	0.007397009	0.558519023
ENSG00000253755.1	1178	IGHGP	IG_C_pseudogene	2.486718819	4.002280416	7.902107798	0.008617072	0.57813851
ENSG00000211664.3	367	IGLV2-18	IG_V_gene	2.520242278	2.86955957	8.408672129	0.006924359	0.554367113
ENSG00000211900.2	61	IGHJ6	IG_J_gene	2.604600943	2.351713787	11.46229445	0.001998051	0.368089868
ENSG00000223350.2	409	IGLV9-49	IG_V_gene	2.609082076	2.649782014	9.540072781	0.004306979	0.475142056
ENSG00000235700.1	310	CYCSP52	processed_pseudogene	2.660345723	-0.470434103	9.659119691	0.004101326	0.47483434
ENSG00000243238.1	390	IGKV2-30	IG_V_gene	2.693528466	5.486181748	12.24754855	0.001478375	0.3581358
ENSG00000278196.3	517	IGLV2-8	IG_V_gene	2.746143696	5.591268265	8.825476527	0.005800841	0.523190066
ENSG00000211669.3	382	IGLV3-10	IG_V_gene	2.779015087	4.946940158	10.33768232	0.003114187	0.43194914
ENSG00000163958.14	2429	ZDHHC19	protein_coding	2.809627426	2.369941751	8.673895306	0.006184793	0.529049382
ENSG00000242076.2	421	IGKV1-33	IG_V_gene	2.809771932	3.857197185	13.18460174	0.001040967	0.341334435
ENSG00000211962.2	655	IGHV1-46	IG_V_gene	2.876941619	4.76685564	10.11960042	0.003400135	0.451570404
ENSG00000253998.3	372	IGKV2-29	IG_V_pseudogene	2.912870932	4.945726798	9.471884444	0.004429765	0.475250539
ENSG00000211897.9	2894	IGHG3	IG_C_gene	2.916868647	8.531943472	11.31024795	0.002119753	0.369491588
ENSG00000211947.2	430	IGHV3-21	IG_V_gene	2.93343086	5.746748746	16.00863867	0.000380788	0.270495492
ENSG00000282600.2	350	IGHV3-69-1	IG_V_pseudogene	2.957199346	1.574564942	8.655543508	0.006233112	0.529049382
ENSG00000211941.3	473	IGHV3-11	IG_V_gene	2.963222839	4.188430303	11.62230649	0.001878048	0.365316646
ENSG00000211955.2	431	IGHV3-33	IG_V_gene	2.97238225	5.305289707	11.15179824	0.002255116	0.380444538
ENSG00000226949.1	945	OR6K5P	unprocessed_pseudogene	2.973120545	-3.402644029	10.75528835	0.003749128	0.468632535
ENSG00000267533.1	1148	AP002414.4	processed_pseudogene	3.029174533	-3.388899267	11.23398878	0.003176336	0.432446069
ENSG00000267340.1	198	AC060780.2	processed_pseudogene	3.102717267	-3.375626423	9.977185235	0.004941328	0.479762477
ENSG00000211890.4	1430	IGHA2	IG_C_gene	3.116901911	7.451557015	16.22413022	0.000353692	0.270495492
ENSG00000211945.2	410	IGHV1-18	IG_V_gene	3.14657691	5.658739752	12.72734214	0.001233907	0.357441589
ENSG00000260571.1	498	BNIP3P5	processed_pseudogene	3.170373816	-2.5681254	7.741719542	0.009242666	0.585027465
ENSG00000279544.1	3109	AL133243.4	TEC	3.184334608	-0.419299165	9.134044394	0.005096495	0.488277743
ENSG00000280408.1	186	AC032044.2	TEC	3.187363697	-3.352676409	10.35814923	0.004311977	0.475142056
ENSG00000241294.1	390	IGKV2-24	IG_V_gene	3.195173044	2.380112445	10.33187455	0.003121457	0.43194914
ENSG00000211950.2	411	IGHV1-24	IG_V_gene	3.223601311	4.644299339	8.7646235	0.005951792	0.523190066
ENSG00000211956.2	400	IGHV4-34	IG_V_gene	3.251518927	6.823588826	15.62014981	0.00043542	0.270495492
ENSG00000211637.2	419	IGLV4-69	IG_V_gene	3.258094589	5.495952462	12.42683402	0.001381422	0.3581358
ENSG00000211967.3	571	IGHV3-53	IG_V_gene	3.292658544	4.250632089	14.76869714	0.00058685	0.283914319
ENSG00000232216.1	434	IGHV3-43	IG_V_gene	3.295154644	4.408869202	10.82894883	0.002560601	0.402792996
ENSG00000272264.1	577	AC009686.2	lncRNA	3.302130391	-2.858001792	7.753690454	0.009194313	0.585027465
ENSG00000199872.1	107	RNU6-942P	snRNA	3.320896771	-2.647470215	10.34023728	0.003110995	0.43194914
ENSG00000211666.2	402	IGLV2-14	IG_V_gene	3.342892311	7.868147978	16.45626182	0.0003268	0.270495492
ENSG00000286546.1	2297	AL079338.1	lncRNA	3.370028796	-2.010548306	7.808629374	0.008975893	0.585027465
ENSG00000238446.1	60	RNU7-38P	snRNA	3.389115204	-3.2957105	9.00641774	0.007060023	0.556519401
ENSG00000238886.1	82	SNORD121A	snoRNA	3.416426924	-3.293590917	10.62066702	0.003930224	0.47483434
ENSG00000211660.3	502	IGLV2-23	IG_V_gene	3.420998567	6.280531381	12.60081737	0.001293843	0.3581358
ENSG00000211946.3	415	IGHV3-20	IG_V_gene	3.425603624	4.250939322	13.30816482	0.000994581	0.340512476
ENSG00000271482.1	260	AC005070.2	processed_pseudogene	3.465072639	-2.788258517	8.114879603	0.007856969	0.562824908
ENSG00000253132.1	340	IGHV3-62	IG_V_pseudogene	3.467590114	-1.780294025	7.572446489	0.009956746	0.60688206
ENSG00000271778.1	578	AC080013.3	lncRNA	3.480626696	-2.781950985	9.437965552	0.004492246	0.477552375
ENSG00000211943.2	437	IGHV3-15	IG_V_gene	3.489451574	4.751140499	19.74462372	0.000111541	0.270495492
ENSG00000251039.2	336	IGKV2D-40	IG_V_gene	3.530378513	1.441583829	7.988381054	0.008299675	0.569568429
ENSG00000211625.2	445	IGKV3D-20	IG_V_gene	3.538727509	4.111913949	9.410755599	0.004543055	0.477827571
ENSG00000279716.1	1661	AC006128.1	TEC	3.584711308	-3.255926148	10.26839268	0.004451807	0.475424414
ENSG00000278473.1	348	IGHV3-41	IG_V_pseudogene	3.640672635	-2.148218247	7.849374877	0.008817537	0.585027465
ENSG00000211973.2	412	IGHV1-69	IG_V_gene	3.65101213	4.059490158	11.67331347	0.001841442	0.365293192
ENSG00000211938.2	430	IGHV3-7	IG_V_gene	3.683943188	5.668379845	17.5713638	0.000224881	0.270495492
ENSG00000273962.1	320	IGKV2-40	IG_V_gene	3.689821878	1.227448293	9.020592946	0.00534409	0.503707486
ENSG00000282639.1	353	IGHV3-64D	IG_V_gene	3.717931881	2.740964227	8.633722194	0.006291097	0.529049382
ENSG00000184925.12	2862	LCN12	protein_coding	3.72710608	-2.452566715	7.942379597	0.008467306	0.576395769
ENSG00000211642.3	567	IGLV10-54	IG_V_gene	3.799477899	3.704585425	15.52770975	0.000449621	0.270495492
ENSG00000211905.1	52	IGHJ1	IG_J_gene	3.844867033	-3.157774153	11.901691	0.002532903	0.401146361
ENSG00000211924.1	31	IGHD3-9	IG_D_gene	3.84640741	-2.385706383	8.632377142	0.00629469	0.529049382
ENSG00000211942.3	430	IGHV3-13	IG_V_gene	3.861879636	3.992317769	11.35362577	0.002084239	0.368198491
ENSG00000238933.1	112	RF00019	misc_RNA	3.870313614	-3.161149416	13.0612961	0.001731407	0.365293192
ENSG00000251307.1	770	AC026704.1	lncRNA	3.89161086	-2.292904212	12.43168245	0.001378897	0.3581358
ENSG00000233040.2	701	FAM204BP	processed_pseudogene	4.03493958	-2.492554644	11.86106926	0.001713169	0.365293192
ENSG00000130950.13	2561	NUTM2F	protein_coding	4.073078802	-1.942227903	11.65920121	0.001851492	0.365293192
ENSG00000271670.1	625	AC010998.2	lncRNA	4.080141007	-3.069885929	12.55093649	0.002043071	0.368089868
ENSG00000241269.1	583	AC093620.1	lncRNA	4.111041589	-3.069309479	13.51405501	0.001498569	0.3581358
ENSG00000256019.1	925	TAS2R63P	unprocessed_pseudogene	4.171282936	-2.325625318	9.266310848	0.004823377	0.477956278
ENSG00000134873.10	3385	CLDN10	protein_coding	4.216200355	-2.556879962	9.627146279	0.004155498	0.47483434
ENSG00000270001.1	491	AL121894.2	lncRNA	4.219037621	-3.016760573	12.99860053	0.001766695	0.365293192
ENSG00000211650.2	397	IGLV5-45	IG_V_gene	4.25057937	4.798524993	15.5053484	0.00045313	0.270495492
ENSG00000261449.1	992	AC103724.3	lncRNA	4.275661739	-2.047838979	11.59217917	0.001900036	0.365576263
ENSG00000213416.4	1092	KRTAP4-12	protein_coding	4.279216156	-2.99201461	11.5086563	0.002891966	0.427397877
ENSG00000207053.1	106	RNU6-937P	snRNA	4.343890566	-2.962583465	12.42858748	0.002126707	0.369491588
ENSG00000211659.2	380	IGLV3-25	IG_V_gene	4.434314672	7.463288856	22.49314237	4.81501E-05	0.224196384
ENSG00000227692.1	531	MED28P3	processed_pseudogene	4.596905236	-1.920473334	13.19446738	0.001037179	0.341334435
ENSG00000211665.3	385	IGLV3-16	IG_V_gene	4.618770932	2.68316428	19.01653557	0.000140535	0.270495492
ENSG00000226180.3	2611	AC010536.1	lncRNA	4.631126336	-1.12733832	14.47681189	0.000651051	0.291482844
ENSG00000271079.1	2587	CTAGE15	protein_coding	4.721031396	-2.79392446	13.47360056	0.001517896	0.3581358
ENSG00000267560.1	637	AC027514.2	lncRNA	4.836446962	-2.314106546	13.49790626	0.00092762	0.327210924
ENSG00000211662.2	602	IGLV3-21	IG_V_gene	4.85904419	7.622066516	16.85556607	0.000285532	0.270495492
ENSG00000278486.1	643	YRDCP1	processed_pseudogene	5.185383125	-2.561517817	13.81536286	0.001362901	0.3581358
ENSG00000225215.1	1186	SMARCE1P1	processed_pseudogene	5.760079142	-2.198891691	12.62973575	0.001991136	0.368089868
ENSG00000211976.2	437	IGHV3-73	IG_V_gene	5.888821666	5.214229038	15.64850314	0.000431162	0.270495492
ENSG00000285665.1	661	AL031274.1	processed_pseudogene	5.891471487	-2.105875324	18.38240444	0.000359377	0.270495492
ENSG00000211670.2	461	IGLV3-9	IG_V_gene	6.06387896	5.693388547	17.78212341	0.000209774	0.270495492
ENSG00000224515.1	462	AL590385.1	lncRNA	6.560596862	0.027441223	17.66113323	0.000218308	0.270495492
ENSG00000225698.3	440	IGHV3-72	IG_V_gene	6.842168346	6.543424479	16.17861364	0.000359238	0.270495492
ENSG00000281990.1	353	IGHV1-69-2	IG_V_gene	7.747357338	1.510998857	25.67621784	1.92785E-05	0.218078169

DEGs, differentially expressed genes; WT, wildtype; Δ382, 382-nt deletion in SARS-CoV-2 ORF8 region; SARS-CoV-2, Severe Acute Respiratory Syndrome Coronavirus 2; FC, fold change.

Supplemental Table 7 Plasma immune mediators of COVID-19 patients at the acute phase of infection (median 8 days PIO; n=25).

	Log_{10} concentration (pg/ml)			
**Cytokine**	**WT (n=14)**	**Δ382 (n=11)**	**Difference (WT vs. Δ382)**	**Healthy Controls (n=23)**
BDNF	34.92	21.85	-13.07	11.10
EGF	0.17	3.63	3.46	0.40
Eotaxin	16.84	14.25	-2.59	26.90
FGF-2	0.18	0.18	0.00	0.20
GM-CSF	0.82	0.82	0.00	1.10
GRO-α	0.05	0.05	0.00	2.50
HGF*	190.00	49.13	-140.90	55.50
IFN-α	0.02	0.02	0.00	0.10
IFN-γ***	6.84	36.14	29.30	17.20
IL-1α	0.19	0.01	-0.18	0.30
IL-1ß	1.90	2.21	0.31	0.80
IL-1RA*	497.60	116.40	-381.30	5.00
IL-2**	12.36	24.20	11.85	12.10
IL-4	0.21	0.21	0.00	5.30
IL-5*	0.04	2.25	2.21	0.10
IL-6	5.99	1.54	-4.45	0.40
IL-7	0.66	0.41	-0.25	0.30
IL-8	0.15	0.15	0.00	0.20
IL-9	2.35	2.35	0.00	2.35
IL-10	0.04	0.04	0.00	0.10
IL-12p70*	0.22	1.06	0.84	0.10
IL-13	0.20	0.20	0.00	0.60
^IL-15**	9.14	1.07	-8.07	1.90
^IL-17A*	0.08	0.08	0.00	0.20
IL-18	34.35	66.66	32.31	29.20
IL-21	0.38	0.38	0.00	1.20
IL-22	0.36	0.36	0.00	9.60
IL-23	0.32	0.32	0.00	0.40
IL-27	0.99	0.99	0.00	2.00
IL-31	2.69	2.69	0.00	2.70
IP-10	26.52	19.34	-7.18	29.00
LIF	5.61	3.48	-2.13	3.10
MCP-1	55.24	44.96	-10.28	43.60
MIP-1α	5.38	0.47	-4.91	2.00
MIP-1ß*	35.81	4.38	-31.43	2.80
PDGF-BB	38.96	45.43	6.48	91.40
PlGF-1	0.76	8.90	8.15	4.70
RANTES	42.51	66.21	23.70	100.90
SCF	4.39	4.53	0.14	4.00
SDF-1α	551.90	586.50	34.60	645.90
TNF-α	6.91	9.23	2.32	1.30
TNF-ß	2.98	2.98	0.00	2.98
VEGF-A*	94.71	37.23	-57.48	1.90
VEGF-D	0.10	0.10	0.00	1.40
β-NGF	0.01	0.58	0.57	2.52

Data presented as median. COVID-19, Coronavirus Disease-19; WT, wild-type; PIO, post-illness onset; BDNF, brain-derived neurotropic factor; EGF, epidermal growth factor; FGF-2, basic fibroblast growth factor; GM-CSF, granulocyte-macrophage colony-stimulating factor; GRO-α, chemokine (C-X-C motif) ligand (CXCL) 1; HGF, hepatocyte growth factor; IFN, interferon; IL, interleukin, IL-1RA, IL-1 receptor antagonist; IP-10, IFN-γ-induced protein 10; LIF, leukemia inhibitory factor; MCP-1, monocyte chemoattractant protein 1; MIP-1α, macrophage inflammatory protein 1α; PDGF-BB, platelet-derived growth factor; PlGF-1, placental growth factor; RANTES, regulated on activation, normal T cell expressed and secreted; SCF, stem cell factor; SDF-1α, stromal cell-derived factor 1α; TNF, tumor necrosis factor; VEGF, vascular endothelial growth factor; β-NGF, beta-nerve growth factor. (*$p < 0.05$; **$p < 0.01$; ***$p < 0.001$). ^Majority of the samples have readings below limit of quantitation (LOQ).

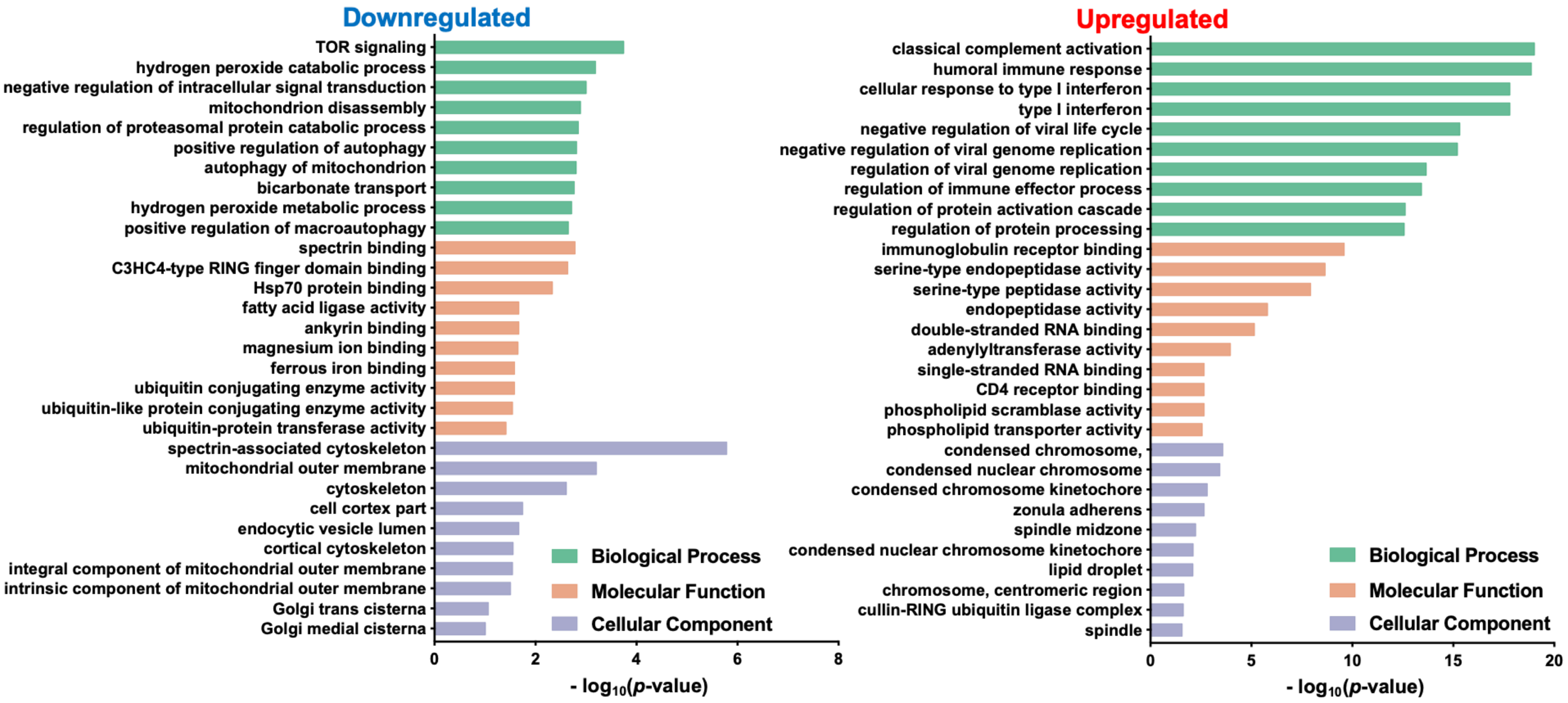

Supplemental Fig. 1

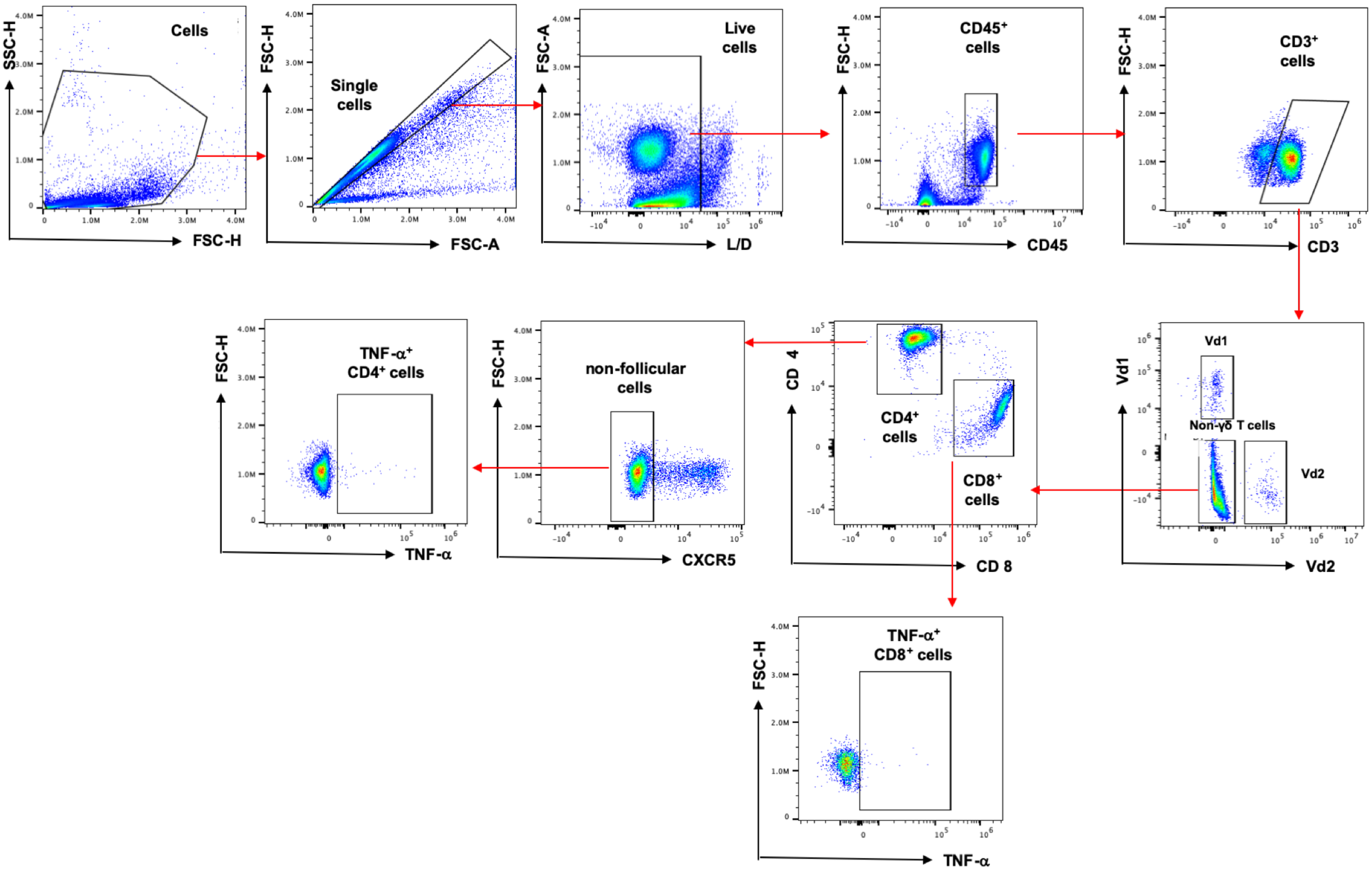

Supplemental Fig. 2

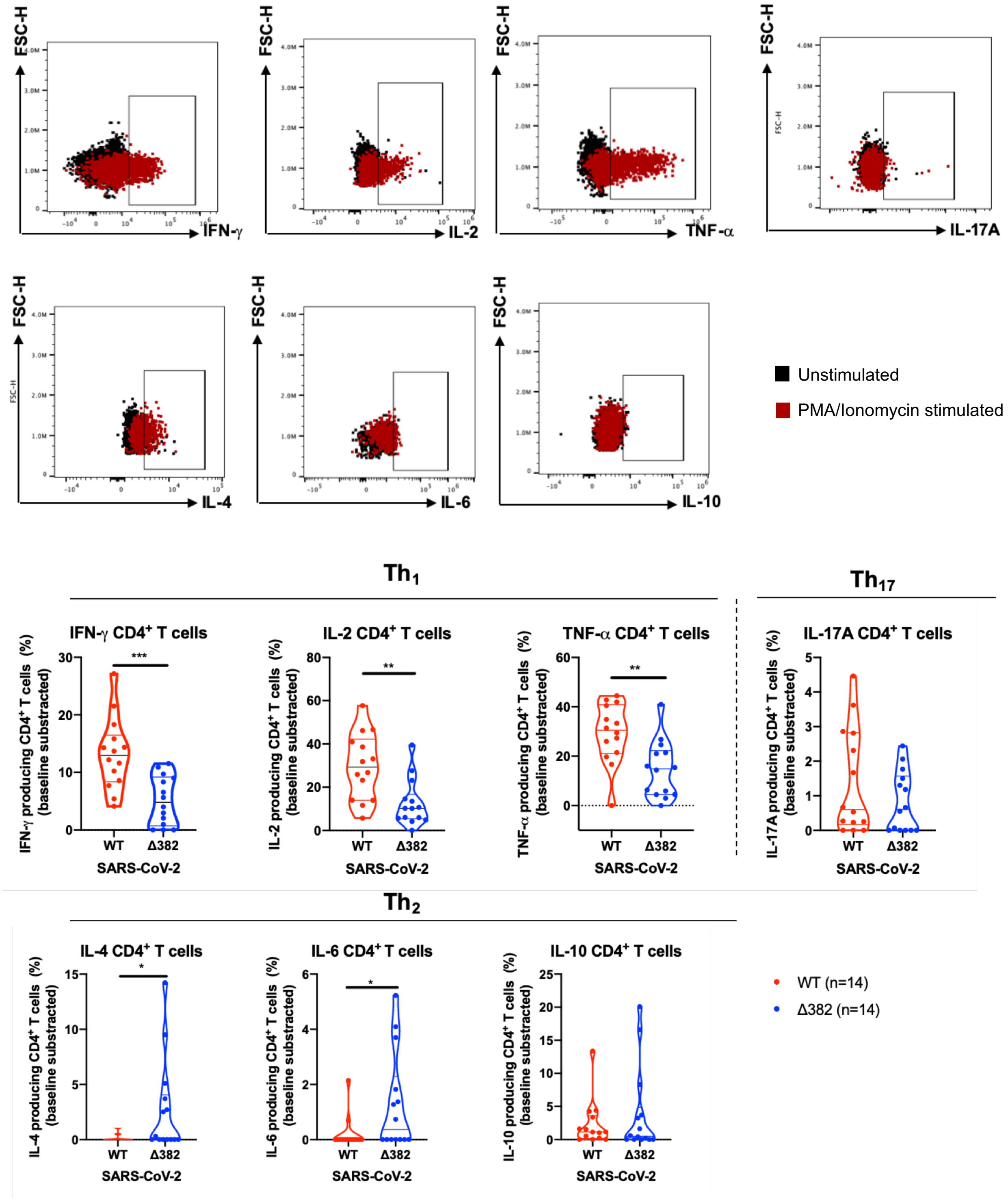

Supplemental Fig. 3
